# Dynamic choice HIV prevention with cabotegravir long-acting injectable in rural Uganda and Kenya: a randomised trial extension

**DOI:** 10.1016/S2352-3018(24)00235-2

**Published:** 2024-10-09

**Authors:** Moses R Kamya, Laura B Balzer, James Ayieko, Jane Kabami, Elijah Kakande, Gabriel Chamie, Nicole Sutter, Helen Sunday, Janice Litunya, Joshua Schwab, John Schrom, Melanie Bacon, Catherine A Koss, Alex R Rinehart, Maya Petersen, Diane V Havlir

**Affiliations:** School of Medicine, Makerere University, Kampala, Uganda; Division of Biostatistics, School of Public Health, University of California, Berkeley, CA, USA; Kenya Medical Research Institute, Nairobi, Kenya; Infectious Diseases Research Collaboration, Kampala, Uganda; Infectious Diseases Research Collaboration, Kampala, Uganda; Division of HIV, Infectious Diseases and Global Medicine, Department of Medicine, University of California, San Francisco, CA, USA; Division of HIV, Infectious Diseases and Global Medicine, Department of Medicine, University of California, San Francisco, CA, USA; Infectious Diseases Research Collaboration, Kampala, Uganda; Kenya Medical Research Institute, Nairobi, Kenya; Division of Biostatistics, School of Public Health, University of California, Berkeley, CA, USA; Division of HIV, Infectious Diseases and Global Medicine, Department of Medicine, University of California, San Francisco, CA, USA; Division of HIV, Infectious Diseases and Global Medicine, Department of Medicine, University of California, San Francisco, CA, USA; National Institute of Allergy and Infectious Diseases, National Institutes of Health, Bethesda, MD, USA; Division of HIV, Infectious Diseases and Global Medicine, Department of Medicine, University of California, San Francisco, CA, USA; ViiV Healthcare, Brentford, UK; Division of Biostatistics, School of Public Health, University of California, Berkeley, CA, USA; Division of HIV, Infectious Diseases and Global Medicine, Department of Medicine, University of California, San Francisco, CA, USA

## Abstract

**Background:**

HIV infections are ongoing globally despite efficacious biomedical prevention options. We sought to determine whether an HIV prevention package providing choice of daily pills or long-acting injectable cabotegravir and opportunities to change prevention options could increase biomedical prevention coverage and reduce new HIV infections.

**Methods:**

This study was an extension of three randomised trials that used SEARCH dynamic choice HIV prevention to recruit adults (aged ≥15 years) at risk for HIV from antenatal, outpatient, and community settings in rural Uganda and Kenya. In this 48-week open-label extension, participants maintained their original (1:1) randomisation group; the option to choose cabotegravir long-acting injectable was added for intervention participants. Inclusion criteria for the extension were previous enrolment in a SEARCH dynamic choice HIV prevention trial, negative HIV rapid test, and residence in study region. The intervention provided person-centred choice of oral pre-exposure prophylaxis (PrEP) or post-exposure HIV prophylaxis (PEP) or cabotegravir long-acting injectable, with the option to switch according to participant preference. The control provided standard-of-care access to oral PrEP and PEP, but not cabotegravir long-acting injectable. Biomedical prevention coverage (proportion of follow-up covered by oral PrEP, PEP, or cabotegravir long-acting injectable; primary outcome) and HIV incidence (secondary outcome) were compared between groups using targeted minimum loss-based estimation. The trial (NCT05549726) is closed to recruitment.

**Findings:**

Of 1534 participants initially randomly assigned (from April 15, 2021 to Sept 29, 2022), 984 (487 in the intervention group and 497 in the standard-of-care group) reconsented to the extension (from Jan 2 to March 3, 2023). The mean proportion of follow-up covered by biomedical HIV prevention was 69·7% (95% CI 64·9–74·5) in the intervention group versus 13·3% (10·2–16·3) in the standard-of-care group, corresponding to an absolute difference of 56·4 percentage points (95% CI 50·8–62·1; p<0·0001). The intervention significantly improved coverage across prespecified subgroups (sex and age groups). During the study, 274 (56%) of 485 intervention participants used cabotegravir long-acting injectable, 255 (53%) used oral PrEP, and ten (2%) used PEP. Among cabotegravir longacting injectable initiators, 118 (43%) of 274 were not previously using oral PrEP or PEP. There were seven incident HIV infections in 390 person-years of follow-up in the standard-of-care group and no infections in 400 person-years of follow-up in the intervention group (incidence rate difference per 100 person-years 1·8, 95% CI 0·4–3·2; p=0·014).

**Interpretation:**

Offering people the choice of HIV biomedical prevention options including cabotegravir long-acting injectable in a flexible model can increase prevention coverage and reduce incident HIV infections. HIV programmes should support dynamic choice HIV prevention programmes that include effective oral and injectable long-acting products.

**Funding:**

National Institutes of Health.

## Introduction

There were 1·3 million new HIV infections in 2023 despite expanded global access to antiretroviral therapy and oral pre-exposure prophylaxis (PrEP).^1^ Until an effective HIV vaccine is discovered, biomedical prevention interventions are crucial for epidemic control. There are now several highly efficacious biomedical HIV prevention options.^2–4^ However, improved approaches for delivering these options effectively at scale are urgently needed.

The number of people estimated globally to have ever used oral PrEP increased from 200 000 in 2017 to 2·5 million in 2023, but overall coverage of PrEP remains well below UNAIDS estimated targets for HIV pandemic control, including in sub-Saharan Africa.^1^ Studies around the world, including those done in sub-Saharan Africa, demonstrate a range of barriers to continuation of oral PrEP during periods of risk, including adherence challenges and stigma.^5,6^ Further, post-exposure prophylaxis (PEP) remains underutilised.^7^

Injectable long-acting cabotegravir (cabotegravir longacting injectable), which requires two injections 1 month apart, followed by injections every 8 weeks on a continual basis, is the latest addition to HIV prevention tools. In head-to-head comparisons, people randomly assigned to cabotegravir long-acting injectable had lower rates of HIV infection than those receiving once per day oral tenofovir disoproxil fumarate plus emtricitabine.^3,4^ Tenofovir disoproxil fumarate plus emtricitabine is highly efficacious for HIV prevention, but cabotegravir long-acting injectable was more effective at reducing incident HIV infections, largely because of product adherence. However, much less is known about the feasibility, acceptability, and adherence to cabotegravir long-acting injectable outside of phase 3 clinical trial settings, including among men and in generalised epidemic settings in rural sub-Saharan Africa.

Offering a choice of biomedical HIV prevention products could improve HIV prevention coverage in these and other settings. Studies have found that clients often switch between products over time on the basis of varying prevention needs and preferences. In several European studies offering a choice of oral or event-driven PrEP among people assigned male at birth, about a third of participants switched between daily and event-driven PrEP.^8,9^ Given that HIV prevention needs and product preferences are dynamic, strategies that offer flexible choices and the option to switch products over time could improve coverage and reduce HIV incidence; however, research evaluating such strategies remains sparse.^10^

In particular, little is known about how to integrate cabotegravir long-acting injectable in such flexible choice-based models, nor the effects of doing so. Qualitative studies and discrete choice experiments have demonstrated strong theoretical interest in longacting prevention products.^11–13^ Trials in which participants were randomly assigned to receive once per day oral PrEP or a long-acting product and subsequently offered a choice between products found that more than two-thirds of participants selected the longacting product over oral PrEP.^14–16^ However, gaps remain in understanding actual choices made by participants in real-world settings, and the extent to which access to cabotegravir long-acting injectable will expand the number of people using biomedical HIV prevention (vs replacing use of alternative prevention options) and decrease HIV incidence in these settings.

In summary, implementation strategies are needed that can effectively deliver biomedical HIV prevention options to populations with heterogeneous and dynamic needs, in rural sub-Saharan Africa and beyond. In response to this evidence gap, we previously did trials of the SEARCH dynamic choice HIV prevention intervention, a person-centred delivery model that offered choice of biomedical prevention product and the ability to switch products over time to men and women in rural Kenya and Uganda (HIV incidence of 0·046 per 100 person-years in Kenya and 0·12 per 100 person-years in Uganda).^17–21^ We showed that when oral PrEP and PEP were the only available biomedical prevention options, this person-centred choice-based strategy resulted in significantly higher self-reported biomedical prevention coverage than standard of care. However, the biomedical prevention coverage achieved by the intervention remained suboptimal; on average only 48·5% of follow-up time was covered by oral PrEP or PEP. Given the potential of cabotegravir long-acting injectable to increase prevention coverage, we evaluated the effect of offering cabotegravir long-acting injectable, daily oral tenofovir disoproxil fumarate plus emtricitabine (oral PrEP), and PEP using our dynamic choice HIV prevention intervention compared with country standard of care on prevention coverage and HIV incident infection in a 48-week extension study.

## Methods

### Study design and participants

This study was an extension of three randomised trials designed to evaluate the effect of a dynamic choice HIV prevention intervention without cabotegravir long-acting injectable on biomedical prevention coverage (appendix p 60). The study participants resided in rural western Uganda and western Kenya and were recruited from Ministry of Health antenatal clinics (two in Uganda, two in Kenya), outpatient departments (two in Uganda, two in Kenya), and from the community (eight villages in Uganda, eight villages in Kenya). Participants were aged at least 15 years, had a negative country-approved HIV rapid test, and self-reported current or anticipated HIV acquisition risk at time of enrolment.

In this 48-week extension, participants continued with their initially assigned group; cabotegravir long-acting injectable was added as an additional biomedical prevention option to the intervention group. We tested the hypothesis that dynamic choice HIV prevention, a person-centred approach to offering participants the choice between cabotegravir long-acting injectable, oral PrEP, and PEP with the ability to switch between these products over time, would achieve higher biomedical HIV prevention coverage for people at risk for HIV infection than country standard of care.

Inclusion criteria for the extension were previous enrolment in a SEARCH dynamic choice HIV prevention trial, negative HIV rapid test, and residence in study region.

The Makerere University School of Medicine Research and Ethics Committee, the Uganda National Council for Science and Technology, the Uganda National Drug Authority, the Kenya Scientific and Ethics Review Unit, the Kenya Pharmacy and Poisons Board, the National Commission for Science, Technology and Innovation, and the University of California San Francisco Human Research Protection Programme reviewed and approved the study protocol. All participants provided written informed consent (in English, Runyankore, Kiswahili, or Dholuo) before study participation.

### Randomisation and masking

At initial enrolment, unmasked randomisation was done in a 1:1 ratio (individual or village level, depending on the setting) to dynamic choice HIV prevention or standard of care, as previously described.^17–20^ Briefly, for the antenatal and outpatient settings, participants who had consented to take part were randomly assigned by selecting a sequentially numbered scratch card; randomisation used a stratified blocked design, with the computer-generated randomisation sequence provided by an independent statistician. For the community setting, study villages were pair-matched within the community and randomisation was done at a meeting of community leaders, in which representatives from each matched pair selected and opened sealed envelopes to reveal the trial group. In this 48-week extension, participants continued in their original randomly-assigned group, and cabotegravir long-acting injectable was added as an option for intervention participants (appendix p 60). Participants, health-care workers, and study staff were not masked to the randomisation group, but the study statistician (LBB) was masked until extension completion and analytic unmasking.

### Procedures

In this study extension, the dynamic choice HIV prevention intervention offered integrated oral PrEP, PEP, and cabotegravir long-acting injectable services at government outpatient clinics, antenatal clinics, and via existing health workers in the community (appendix p 52). The intervention included provider training in offering choices between biomedical prevention products, prevention counselling, and structured assessment of barriers to product use and personalised actions to overcome them.^20^

Participants were not locked into their initial biomedical prevention choice. Instead, they could change products over time on the basis of self-assessed preferences and needs. To facilitate dynamic choice, the intervention included structured visits every 12 weeks (and as needed, for example for PEP). Participants choosing oral PrEP or PEP had the option of HIV self-testing or country-approved rapid test (Kenya, Determine and First Response; Uganda, Determine and Statpak), and the option of medication delivery by a community health worker. Participants who were taking no biomedical prevention had the option of HIV self-testing or country-approved rapid test, done by either a community health worker or provider. Participants were provided a clinical officer or nurse mobile telephone number to ask questions and for notification of PEP start.

Medical personnel administered cabotegravir long-acting injectable as a single 3 mL injection of 600 mg cabotegravir. No cabotegravir oral lead-in was done. The first two injections were separated by 4 weeks; subsequently, injections were every 8 weeks. Participants more than 8 weeks late for an injection restarted with two injections separated by 4 weeks before resuming the 8-week injection schedule. Eligibility criteria to start cabotegravir long-acting injectable included not being pregnant at the time of initial cabotegravir long-acting injectable injection, having a weight higher than 35 kg, having a negative HIV RNA measure (Cepheid; Sunnyvale, CA, USA), and having had a country-approved rapid HIV test. Before subsequent injections, a country-approved HIV rapid test was done to exclude HIV infection. Additional exclusion criteria for cabotegravir long-acting injectable access included alanine transaminase five or more times the upper limit of normal and known cirrhosis or severe liver disease (appendix p 4). Participants with planned travel that would disrupt injection visits were provided bridging oral PrEP (tenofovir disoproxil fumarate plus emtricitabine). Women receiving cabotegravir long-acting injectable who became pregnant during the course of the study were offered the option of continuing cabotegravir long-acting injectable under informed consent that included post-partum monitoring of maternal and infant outcomes.

Control participants had access to oral PrEP or PEP according to country guidelines, which permitted, but did not facilitate, switch between prevention modalities. At the start of the extension, the participants in the standard-of-care group were referred to HIV PrEP and PEP services at the nearest government health centre. Participants in the standard-of-care group were not provided options for HIV self-testing, nor access to a medical provider’s mobile telephone number (appendix p 52).

In both groups, participant evaluations were done at 24 weeks and 48 weeks (allowing for visits up to 8 weeks early or late for the final 48-week visit), which included a retrospective 6-month survey of HIV prevention product use and of self-perceived risk of HIV acquisition. Specifically, for each of the preceding 6 months under follow-up, participants were asked if they had swallowed any PrEP or PEP pills and if they felt they were at risk of HIV. For detection of incident HIV infections, country-approved HIV rapid tests and HIV RNA measures were done at 24 weeks and 48 weeks in both groups. HIV testing was also available throughout follow-up to all participants at government health centres. All participants with detectable HIV RNA and negative rapid antibody tests had repeat antibody testing for confirmation of HIV diagnosis. For participants who started cabotegravir long-acting injectable, grade 3 and grade 4 adverse events and adverse drug reactions leading to product discontinuation were recorded using the National Institute of Allergy and Infectious Diseases Adverse Event Grading Scale.^22^

### Outcomes

The primary outcome was biomedical HIV prevention coverage, defined as the proportion of follow-up time covered by any of oral PrEP, PEP, or cabotegravir long-acting injectable. Follow-up started at reconsent for the extension and ended at the earliest of HIV diagnosis, death, withdrawal, or on Dec 31, 2023. Follow-up time was censored during periods without data on use of biomedical HIV prevention. For each follow-up month, participants were classified as covered for a given month if they reported any pill ingestion of oral PrEP or PEP during that month (assessed during retrospective 24-week and 48-week surveys), or if they were covered by cabotegravir long-acting injectable injections during that month. For cabotegravir long-acting injectable, coverage was defined through injection logs; coverage began 3 days after injection and continued for 67 days.

Incident HIV infection was a secondary endpoint. Confirmed HIV infection required at least two different HIV rapid antibody tests or HIV RNA plus subsequent HIV antibody confirmation. A committee of HIV clinical experts adjudicated HIV incident infections masked to study group. Prevention coverage during periods of self-perceived HIV risk (retrospectively assessed using 24-week and 48-week surveys) was assessed as an additional secondary endpoint.

### Statistical analysis

Biomedical HIV prevention coverage was compared between groups with targeted minimum loss-based estimation, with adaptive adjustment for baseline covariates to maximise precision.^23^ The primary analysis pooled participants across the trials, adjusted for recruitment setting (antenatal clinic, outpatient department, and community), and used cross-validation to select additional adjustment variables (sex, age, alcohol use, and mobility). Sex and age group (15–24 years and ≥25 years) were prespecified subgroups; Bonferroni adjustment controlled for multiple testing. The primary analysis excluded participants whose endpoint was not ascertained. In prespecified sensitivity analysis, we assessed the robustness of our findings with targeted minimum loss-based estimation adjusting for fixed and time-varying differences between participants who did versus those who did not continue in the extension and who did versus those who did not have their endpoint ascertained (see statistical analysis plan, appendix p 45). In the sensitivity analysis, the adjustment set included trial groups, country, recruitment site, sex, age, alcohol use, mobility, and biomedical prevention coverage and HIV risk prior to the extension start.

Analogous analyses compared prevention coverage during periods of self-reported risk. HIV incidence rates were calculated using person time at risk and compared between groups without adjustment. For all endpoints, statistical inference was based on the estimated influence-curve, accounting for clustering (see statistical analysis plan, appendix p 45). All analyses were done in R version 4.3.3. This trial is registered with ClinicalTrials.gov, NCT05549726. The study was done in accordance with the Declaration of Helsinki with oversight by the Data Safety and Monitoring Board.

### Role of the funding source

This was an investigator-initiated and designed study of the SEARCH collaboration supported by the Division of AIDS of the National Institute of Allergy and Infectious Diseases. ViiV Healthcare provided cabotegravir long-acting injectable.

## Results

**Figure 1:**
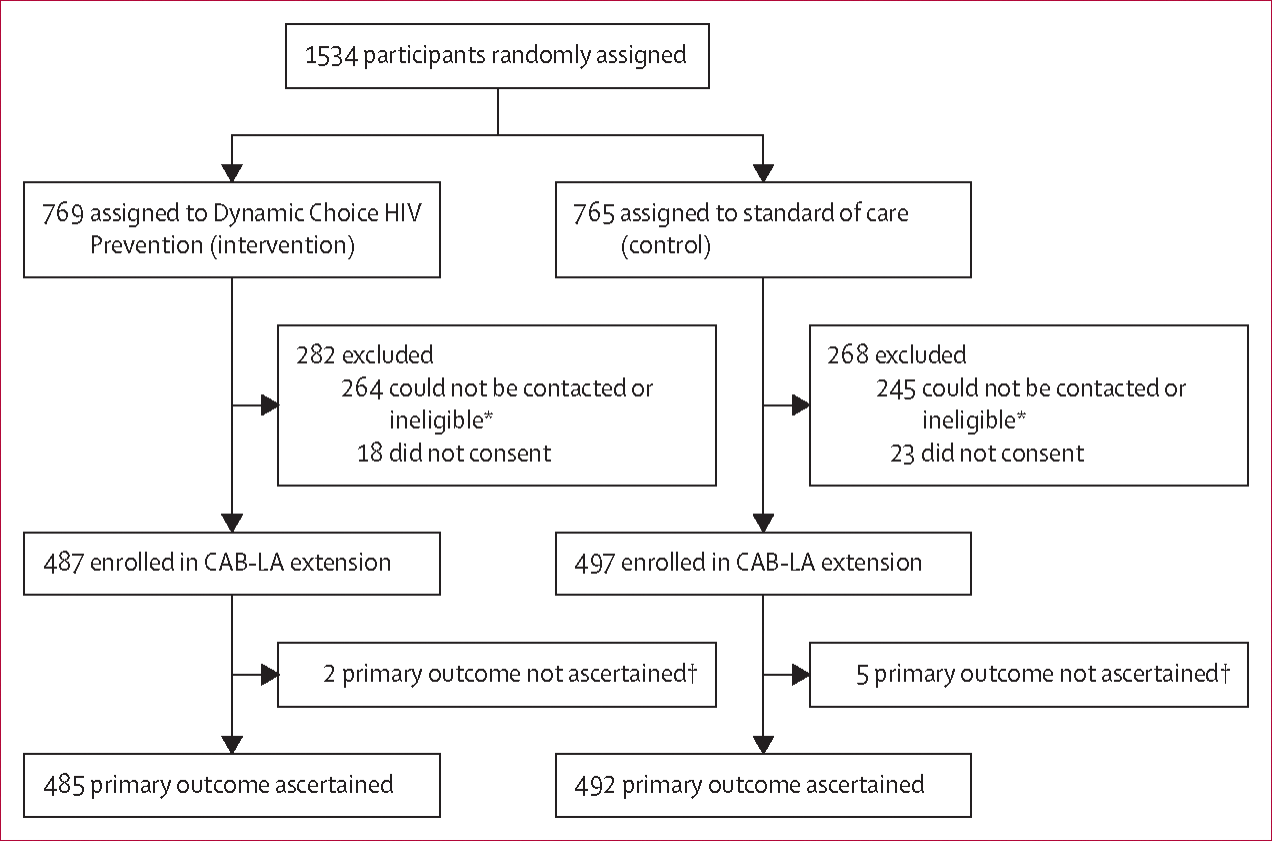
Trial profile *Ineligible if moved out of region, acquired HIV, or withdrew before the extension. †No data on biomedical HIV prevention coverage.

Between Jan 2, 2023 and March 3, 2023, 984 (64%) of the 1534 participants (487 in the intervention group and 497 in the control group) originally enrolled (between April 15, 2021 and Sept 29, 2022) in the SEARCH dynamic choice prevention trials reconsented to participate in the extension (figure 1); follow-up ended on Dec 31, 2023. The main reasons participants did not reconsent were that they had moved out of the region, had acquired HIV, withdrew from follow-up, or were unable to be contacted before the extension; reconsenting was balanced by group. The primary endpoint was ascertained in 977 (99%) of 984 participants: 485 (100%) of 487 participants in the intervention group and 492 (99%) of 497 participants in the standard-of-care group (figure 1). During the 48-week extension, there were 822·1 person-years of follow-up overall: 414·4 person-years of follow-up in the dynamic choice HIV prevention intervention group and 407·7 person-years of follow-up in the standard-of-care group. The median (first and third quartiles) follow-up time was 0·86 person-years (95% CI 0·83–0·91) in the intervention group and 0·84 person-years (0·82–0·89) in the standard-of-care group.

At the extension start, participant characteristics were similar between groups (table 1). 314 (32%) of 984 participants were recruited from the antenatal clinic, 295 (30%) from outpatient departments, and 375 (38%) from the community. 716 (73%) of 984 participants were women, among whom 59 (8%) were pregnant at reconsent, and 268 (27%) were men.

**Figure 2:**
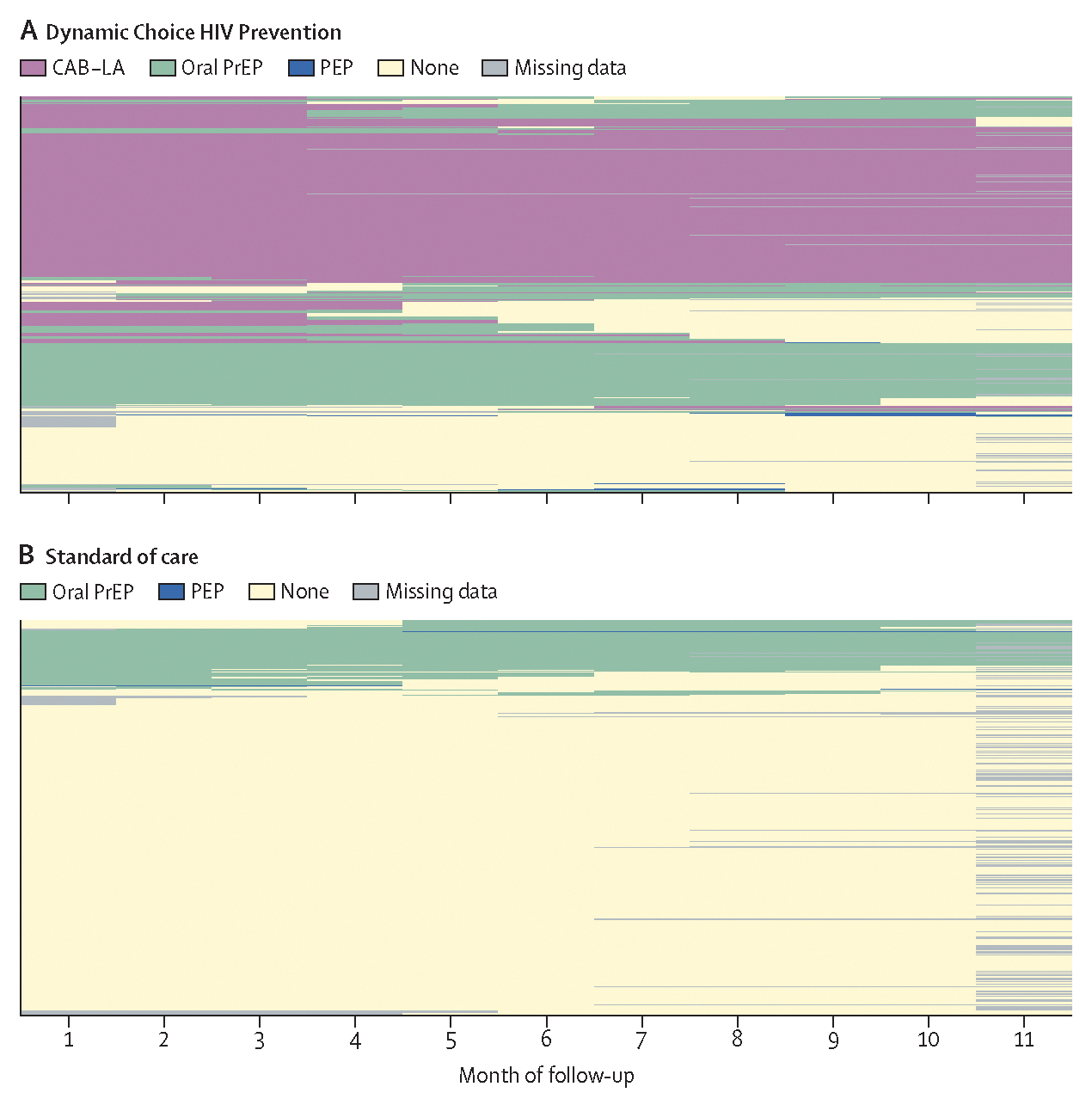
Biomedical HIV prevention product use over time Biomedical HIV prevention product use over time in the dynamic choice HIV prevention group (A) and in the standard-of-care group (B). Each row corresponds to a participant and each column a month of follow-up. CAB-LA=long-acting cabotegravir injectable (intervention group only). Oral PrEP=oral pre-exposure prophylaxis (daily oral tenofovir disoproxil fumarate–emtricitabine). PEP=post-exposure prophylaxis.

We used heatmaps to visualise prevention product use (retrospectively assessed via self-reported pill ingestion for oral PrEP and PEP, and via injection log for cabotegravir long-acting injectable) over time by group (figure 2). In the first month of the extension study in the standard-of-care group, 74 (15%) of 481 participants used oral PrEP, one (<1%) used PEP, and 406 (84%) used no biomedical prevention product. In the first month in the dynamic choice HIV prevention group, 250 (52%) of 481 participants used cabotegravir long-acting injectable, 116 (24%) used oral PrEP, two (<1%) used PEP, and 113 (23%) used no biomedical prevention product.

Among intervention participants who initially used no biomedical prevention product, 32 (28%) of 113 participants subsequently used at least one biomedical prevention product during follow-up; 11 (10%) used cabotegravir long-acting injectable, 18 (16%) used oral PrEP, and six (5%) used PEP. During the extension period, there were 19 total courses of PEP, dispensed to ten participants; among PEP users, seven participants used several PEP courses. During follow-up two of ten participants initially using PEP subsequently transitioned to PrEP.

Overall in the intervention group, 82 (64%) of 129 men and 168 (147%) of 358 women initiated cabotegravir long-acting injectable at study start (appendix p 53). Among participants who initiated cabotegravir long-acting injectable at study start, 142 (57%) of 250 switched from oral PrEP, three (1%) switched from PEP, and 105 (42%) were not on any biomedical prevention product in the previous month (appendix p 54).

During the 48-week follow-up period of the extension study, 274 (56%) of 485 intervention participants had ever used cabotegravir long-acting injectable, 255 (53%) used oral PrEP, and ten (2%) used PEP; median for duration of use among participants that used each of these modalities was 11 months (first and third quartile 6, 11), 7 months (6, 11), and 3 months (2, 4). Among standard-of-care participants, 92 (19%) of 492 had ever used oral PrEP and three (1%) used PEP. 134 (28%) of 485 participants in the intervention groups and two (0%) of 492 participants in the standard-of-care groups used at least two different products during the study.

**Figure 3:**
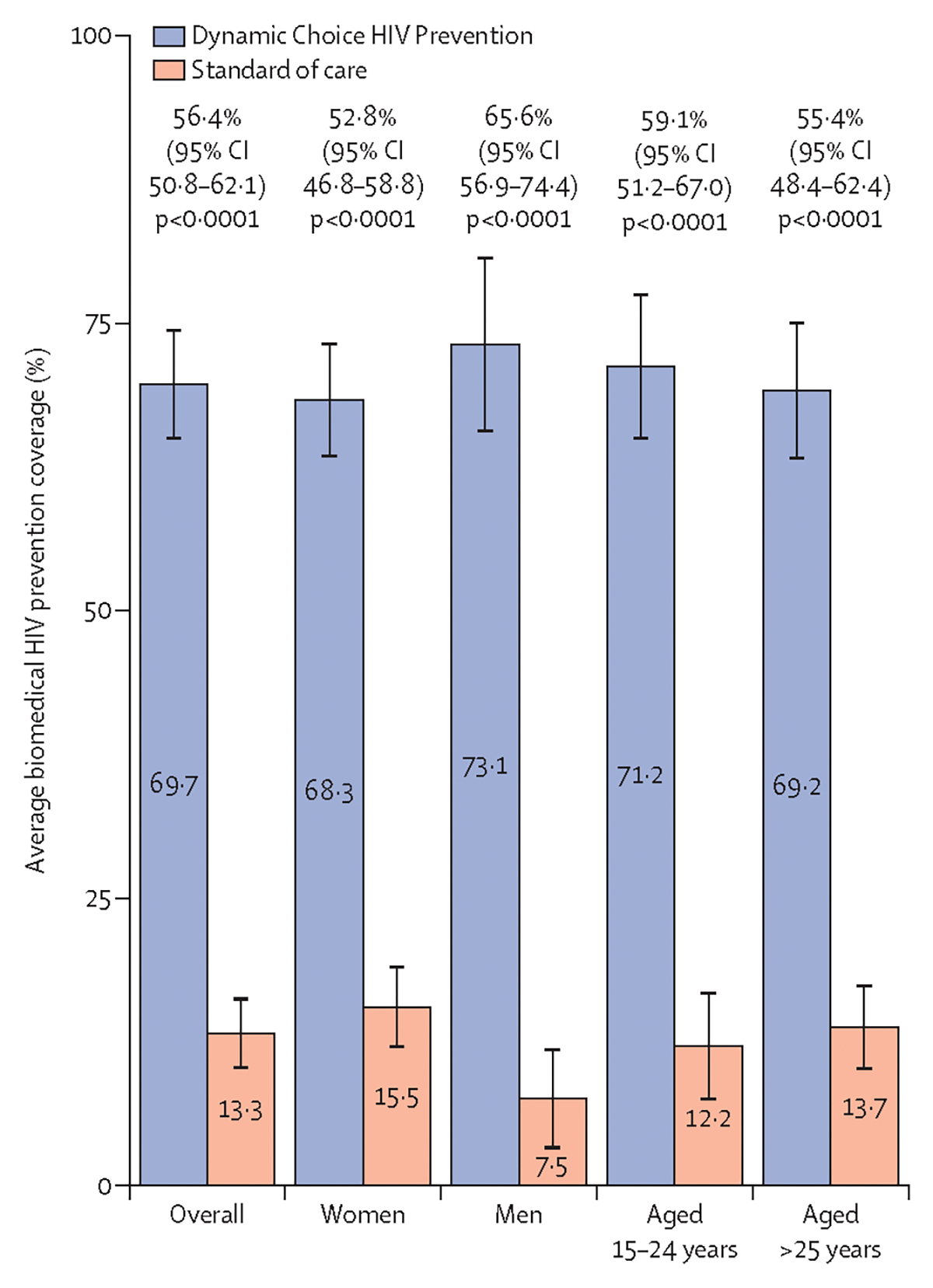
Effect of dynamic choice HIV prevention versus standard of care on Biomedical HIV prevention coverage Biomedical HIV prevention coverage is the proportion of follow-up time during which a participant used cabotegravir long-acting injectable, oral PrEP, or PEP. Group-specific means are reported overall and by prespecified subgroups. The absolute difference in mean biomedical HIV prevention coverage between groups is presented with 95% CI shown in parentheses. p values are adjusted for multiple testing using the Bonferonni method. CAB-LA=long-acting cabotegravir injectable. Oral PrEP=oral pre-exposure prophylaxis. PEP=post-exposure prophylaxis.

The mean proportion of follow-up time covered by biomedical HIV prevention was 69·7% (95% CI 64·9–74·5) among dynamic choice HIV prevention participants and 13·3% (10·2–16·3) among participants in the standard-of-care group, corresponding to an absolute effect size of 56·4 percentage points (95% CI 50·8–62·1; p<0·0001; figure 3). Dynamic choice HIV prevention with cabotegravir long-acting injectable improved coverage versus standard of care across key subgroups, including among women (52·8 percentage points, 95% CI 46·8–58·8), men (65·6 percentage points, 56·9–74·4), younger participants aged 15–24 years (59·1 percentage points, 51·2–67·0), and older participants aged at least 25 years (55·4 percentage points, 48·4–62·4). Effect estimates were robust to a range of sensitivity analyses adjusting for overall differences and differences by group in characteristics of participants who did versus those who did not continue in the extension (appendix p 55). Unadjusted estimates of the overall effect were also similar, 56·8 percentage points (95% CI 50·4–63·1; p<0·0001).

There were seven incident HIV infections in 390 person-years of follow-up in the standard-of-care group and no infections in 400 person-years of follow-up in the intervention group (incidence rate difference per 100 person-years 1·8, 95% CI 0·4–3·2; p=0·014). Among participants in the standard-of-care group who acquired HIV infection, five of seven were women; their ages ranged from 23 years to 43 years (table 2; appendix p 57). Notably, an additional incident HIV infection in an infant aged 7 months resulted from HIV acquisition by a female participant in the standard-of-care group.

Average proportion of follow-up time covered with HIV biomedical prevention during periods of self-reported HIV risk was 76·5% (95% CI 71·1–81·8) among participants in the dynamic choice HIV prevention group and 16·2% (12·7–19·7) among participants in the standard-of-care group, an absolute effect size of 60·2 percentage points (95% CI 53·8–66·6; p<0·0001). The intervention improved at-risk coverage across key subgroups, including among women (57·2 percentage points, 50·5–63·9), men (68·3 percentage points, 58·2–78·5), younger participants aged 15–24 years (63·3 percentage points, 54·6–72·1), and older participants aged at least 25 years (59·1 percentage points, 51·5–66·8; appendix p 61). As before, results were robust to sensitivity analyses accounting for differences between participants who did versus those who did not continue in the extension (appendix p 58).

Study participants who received at least one injection of cabotegravir long-acting injectable were followed up for grade 3 and grade 4 adverse events. There were two cases of trauma (ocular injury and physical altercation) that led to hospital admission, one miscarriage following serious blunt trauma, and the death of premature twins 6 months after the last injection of cabotegravir long-acting injectable. Grade 2 rashes were observed within 15 weeks following initial cabotegravir long-acting injectable injections in seven (3%) of 274 participants, with unknown relation to cabotegravir long-acting injectable; rashes were noted among participants at two of the four sites in Uganda and one of the four sites in Kenya. No hypersensitivity reactions were observed. Cabo tegravir long-acting injectable was discontinued in these seven participants out of caution in this rural study setting.

## Discussion

Our study demonstrated that a dynamic choice HIV prevention intervention that provided participant choice between cabotegravir long-acting injectable, oral PrEP, and PEP and the ability to change products over time increased HIV biomedical prevention coverage by five times compared with standard of care and resulted in no incident HIV infections among men and women in rural settings in Uganda and Kenya.

The global burden of HIV remains highest in sub-Saharan Africa. Although efficacious biomedical prevention products, including oral PrEP and PEP, are available, coverage remains suboptimal; in many regions, progress towards ending the epidemic has stalled.^1^ Effective approaches for delivering biomedical HIV prevention to meet the diverse and dynamic needs of both women and men, including in rural settings, are urgently needed. Further evidence is also needed on choice-based models for delivering HIV prevention in real-world settings, and whether incorporating cabotegravir long-acting injectable as an option in these models will enhance the effects.^10^ Our study helped to fill these gaps.

The SEARCH dynamic choice HIV prevention intervention was anchored in person-centred care and was offered in government clinics and the community.^20^ The intervention included provider training on how to offer choices in a way that maximised client agency, in the context of warm and respectful interactions and accessibility to address questions or concerns. Our study thus provides an opportunity to understand the HIV biomedical prevention coverage reached when cabotegravir long-acting injectable is included on the prevention menu with oral PrEP and PEP, in a manner that enables client-driven (*vs* provider-driven) choice. Our findings demonstrate that when choice was offered in this manner, participants chose different products, and, importantly, modified their choices over time.

In particular, our study is among the first to document the choices actually made (revealed preferences over time) by adults when offered structured choices inclusive of cabotegravir long-acting injectable for HIV prevention. Discrete choice experiments for HIV prevention inclusive of injectable PrEP reveal an overall preference for an injectable long-acting option, with heterogeneity across subpopulations.^11–13^ In addition, when the landmark phase 2b–3 randomised studies of cabotegravir longacting injectable, which assigned participants to a fixed product—either cabotegravir long-acting injectable or oral PrEP, were extended to allow participants a choice of either product, the majority of participants chose injectable cabotegravir long-acting injectable.^14,16^ In our study, more than half of participants initially chose cabotegravir long-acting injectable, underscoring high demand for this product. However, many participants chose an oral product instead, and 28% of participants used at least two products during 48 weeks of follow-up.

Motivations for participant product choice varied. As reported by others,^5–6^ for some participants in our study, addition of cabotegravir long-acting injectable as an option helped overcome barriers to oral PrEP, including adherence challenges and stigma (often exacerbated by resemblance of PrEP to HIV treatment). However, cabotegravir long-acting injectable requires injections, which commonly elicit initial local site reactions and require travel to a health facility; for some participants, oral PrEP or PEP were preferred alternatives. PEP, in particular, could have an important role in the context of shifting risk perceptions and unpredictable risk.^7^ In particular, although all participants initially deemed themselves as at risk for HIV acquisition, they might not have perceived themselves as continually at risk. Product choice that included a PEP option for unanticipated risk allowed participants to engage actively in a self-directed preventive health approach for HIV and might have contributed to ongoing engagement in prevention services and as a bridge to PrEP for some participants. In summary, these observations suggest that any one-size-fits-all option is likely to fall short of prevention goals and highlights the key role of effectively supporting choice, offered in a person-centred and dynamic model, to optimise biomedical prevention coverage and reduce incident HIV infections.

The importance of choice is likely to persist when other biomedical prevention products are offered. In MTN-034/REACH,^15^ in which young women were randomly assigned to receive the dapivirine vaginal ring or oral PrEP for 6 months each, followed by 6 months of product choice, two-thirds chose the dapivirine ring. Importantly, a third of participants preferred oral PrEP, with high PrEP adherence in the randomised phase predictive of oral PrEP choice. Notably, in this trial, and in the HPTN 083/084 open-label extensions,^14,16^ participants were initially randomly assigned to receive a product; more data are needed on product choice, including the ring and cabotegravir long-acting injectable, when offered in roll-out settings. Indeed, several other studies are planned or have begun to offer a choice of products, including cabotegravir long-acting injectable, in sub-Saharan Africa.

A key question in HIV biomedical prevention implementation is whether offering choice of cabotegravir long-acting injectable will expand overall prevention coverage or instead substitute a preferred injectable option for people already taking oral PrEP. We found that 42% of people who started cabotegravir long-acting injectable when given the option were on neither oral PrEP nor PEP at the time, despite having access to these oral options delivered using a person-centred model. This finding suggests that offering cabotegravir longacting injectable will expand overall prevention coverage. A switch to cabotegravir long-acting injectable by people already on oral PrEP or PEP could further improve prevention efficacy if adherence to oral regimens is suboptimal. Thus, there is a dual benefit of adding cabotegravir long-acting injectable in a dynamic choice HIV prevention model.

Men lag behind women globally on both HIV care and prevention cascades.^24,25^ In sub-Saharan Africa, biomedical prevention interventions have been studied less extensively among men than women; however, in studies of oral PrEP that included men and women in Kenya, uptake and retention challenges were observed in both sexes.^26^ Cabotegravir long-acting injectable for prevention has only been studied in women and a small number of men who have sex with men in Africa.^3,4^ Surveys among South African heterosexual men showed demand for cabotegravir long-acting injectable and high acceptability.^27^ Our study enrolled more women than men because the initial study recruitment sites included antenatal clinics; however, more than 25% of participants were men. Our study is the first in Africa to show that men are willing and able to take cabotegravir long-acting injectable and that they benefited from a dynamic choice intervention that included cabotegravir long-acting injectable as an option.

Our study was implemented in a real-world setting without cabotegravir long-acting injectable oral lead-in, with accommodations for travel (bridging oral PrEP), and with an option to continue cabotegravir long-acting injectable for women who became pregnant. Oral lead-in before cabotegravir long-acting injectable initiation is sometimes given to assess drug tolerability; however, so-called direct to inject, with no oral lead-in, is considered safe and supported by guidelines.^28^ Similarly, offering choice of oral PrEP when travel interferes with scheduled cabotegravir long-acting injectable injections can ensure continuity of protection.

Our study has limitations. First, in this extension study, participants maintained their original randomisation group; therefore, balance between groups was not guaranteed at extension start. Similar effect sizes were observed in a range of sensitivity analyses adjusting for differences between groups at extension start, including between people who did and did not enrol in the extension; however, unmeasured differences could have persisted. Second, our primary outcome of biomedical prevention coverage used self-reported pill ingestion for oral PrEP and PEP (a measure that does not equate to protection from HIV acquisition and is potentially subject to recall bias); cabotegravir injections were documented via logs. We previously used hair biomarkers of drugs to validate self-reported product use and found no evidence of difference by group.^17^ Importantly, the significant difference in HIV incidence observed between groups validates that the intervention meaningfully improved protection from HIV acquisition during periods at risk. Third, we relied on self-reported risk and did not collect information on or use classifications such as sex worker, men who have sex with men, or transgender women or men. Finally, we evaluated the effect of a combination intervention in which facilitated dynamic product choice was combined with additional intervention components (including increased access to clinicians, and HIV testing modality and visit location choice for participants not choosing cabotegravir long-acting injectable), complicating attribution of intervention effects; additional mixed-methods analyses to understand the role of intervention components are ongoing.^17–20^

The feasibility, effect, and cost-effectiveness at scale of the dynamic choice prevention intervention incorporating cabotegravir long-acting injectable also remain to be determined. Although some previous modelling analyses have supported the cost-effectiveness of cabotegravir long-acting injectable at scale in sub-Saharan Africa, others have questioned the financial feasibility of such a strategy.^29,30^ Previous analyses have been limited, however, by the paucity of data available to inform the distribution of choices between products and the overall prevention coverage and incidence reductions likely to be achieved using a combined choice-driven delivery strategy. Our study provides novel data to improve the robustness of these key model inputs. Additional modelling and cost-effectiveness analyses are ongoing to inform ongoing country and global policy decisions surrounding the roll-out of long-acting injectable prevention options including cabotegravir long-acting injectable in the context of dynamic choice.

In conclusion, the SEARCH person-centred model for delivering choice in HIV biomedical products, including cabotegravir long-acting injectable, oral PrEP, and PEP, with the option to change products over time, expanded biomedical HIV prevention coverage for women and men, and was highly effective in reducing incident HIV infections in a real-world setting. Efforts to expand access to cabotegravir long-acting injectable globally must be accelerated and should be offered in a dynamic choice HIV prevention model.

## Supplementary Material

supplement

## Figures and Tables

**Table 1:** Demographic characteristics of the participants at extension start

	Dynamic choice HIV prevention (n=487)	Standard of care (n=497)	p value

Age group			0·27
15–24 years	139 (29%)	159 (32%)	
≥25 years	348 (71%)	338 (68%)	
Sex			0·65
Female	358 (74%)	358 (72%)	
Male	129 (26%)	139 (28%)	
Country			0·66
Kenya	245 (50%)	258 (52%)	
Uganda	242 (50%)	239 (48%)	
Recruitment setting			0·42
Community	180 (37%)	195 (39%)	
Antenatal clinic	165 (34%)	149 (30%)	
Outpatient department	142 (29%)	153 (31%)	
Marital status			0·49
Married or cohabitating	394 (81%)	389 (78%)	
Single (never married)	65 (13%)	71 (14%)	
Divorced, separated, or widowed	28 (6%)	37 (7%)	
Occupation			0·023
Farmer	201 (41%)	183 (37%)	
Shopkeeper or market vendor	52 (11%)	28 (6%)	
Student	23 (5%)	41 (8%)	
Manual labour or construction	23 (5%)	44 (9%)	
Transportation	13 (3%)	13 (3%)	
Bar, hotel, or restaurant worker	13 (3%)	15 (3%)	
Fisher or fishmonger	9 (2%)	8 (2%)	
Alcohol use*	84 (17%)	76 (15%)	0·46
Circumcised†	76 (59%)	79 (57%)	0·83
Pregnant‡	32 (9%)	27 (8%)	0·59

Data are n (%). Percentage may not total 100 because of rounding.

* Alcohol use was defined as reporting drinking one or more alcoholic beverages per week.

† Summary statistics are for male participants.

‡ Summary statistics are for female participants.

**Table 2:** HIV incident infections

	Number of incident infections/persons-years at risk (HIV incidence per 100 person-years)		Difference in incidence rates per 100 person-years (95% CI)
	Dynamic Choice HIV Prevention	Standard of care	

Overall	0/400 (0·0/100)	7/390 (1·8/100)	−1·8 (−3·2 to −0·4)
Sex			
Female	0/293 (0·0/100)	5/283 (1·8/100)	−1·8 (−3·3 to −0·2)
Male	0/107 (0·0/100)	2/106 (1·9/100)	−1·9 (−5·5 to 1·7)
Age			
15·24 years	0/113 (0·0/100)	1/122 (0·8/100)	−0·8 (−2·4 to 0·8)
≥25 years	0/287 (0·0/100)	6/268 (2·2/100)	−2·2 (−4·2 to −0·3)

## Data Availability

Deidentified, participant-level data will be made available approximately 1 year after completion of the ongoing trial (NCT05549726), following approval of a concept sheet summarising the analyses to be done. Further inquiries can be directed to the SEARCH Scientific Committee at douglas.black@ucsf.edu. The study protocol and statistical analysis plan are available in the appendix (pp 4, 45).

## Research in context

### Evidence before this study

Oral pre-exposure prophylaxis (PrEP) programmes exist in many sub-Saharan countries, but in many settings declines in HIV incidence have plateaued. Two flagship, randomised trials (HIV Prevention Trials Network 083 and 084) provided evidence for efficacy of long-acting cabotegravir injections for PrEP. Further, discrete choice experiments for HIV prevention reveal an overall preference for an injectable long-acting option. However, in most of sub-Saharan Africa, cabotegravir long-acting injectable has yet not been offered in real-world settings as an additional prevention choice, in the context of other biomedical prevention options, for people at risk of HIV. We searched PubMed for articles published in English from database inception to April 8, 2024 using the terms “HIV prevention” AND “cabotegravir” AND “choice”. We identified 13 articles. These articles emphasised the importance of and potential for cabotegravir long-acting injectable to provide an effective option for HIV prevention, but there were no completed trials offering choice of the biomedical prevention products oral PrEP, oral post-exposure HIV prophylaxis (PEP), and long-acting cabotegravir injectable as part f oa patient-centred dynamic prevention model. One article described an ongoing study looking at cabotegravir long-acting injectable as a choice added to the standard of care in Brazil. To our knowledge, our study is the first to evaluate a dynamic choice HIV prevention model that includes long-acting cabotegravir injectable alongside oral PrEP and PEP on prevention coverage and incident HIV infection.

### Added value of this study

This study was designed to assess the effect of the SEARCH dynamic choice model of HIV prevention on biomedical prevention coverage and incident HIV infections compared with standard of care among men and women with self-assessed risk of HIV acquisition in rural settings in Uganda and Kenya. The dynamic choice model offered person-centred and integrated oral PrEP, post-exposure prophylaxis, and cabotegravir long-acting injectable services for both men and women with self-assessed risk for HIV acquisition at government outpatient clinics, antenatal clinics, and via existing health workers in the community. The intervention included provider training in offering choices between biomedical prevention products, prevention counselling, and structured assessment of barriers to product use and personalised actions to overcome them. Participants could change products over time on the basis of their preferences and needs. Participants used different options during the study; more than half of participants used cabotegravir long-acting injectable. Our intervention increased biomedical HIV prevention coverage by five times compared with standard care and resulted in no incident HIV infections compared with seven infections in the standard-of-care group at 48 weeks. Our study was implemented in a real-world setting without cabotegravir long-acting injectable oral lead-in, with accommodations for travel (bridging oral PrEP), and with an option to continue cabotegravir long-acting injectable for women who became pregnant. Thus, our approach allows generalisability to the current HIV care and prevention environment in rural sub-Saharan Africa.

### Implications of all the available evidence

Our study adds to growing evidence of the key role for client choices in optimising HIV prevention coverage for people at elevated risk of HIV infection, and for the provision of cabotegravir long-acting injectable as one of several prevention options for both women and men with perceived risk of acquisition of HIV infection. Previous work has demonstrated that offering women choices in product and delivery can improve contraception coverage; our study demonstrates this for biomedical HIV prevention. Interventions such as the SEARCH dynamic choice HIV prevention model, which supports client choice of product to people at risk of HIV, should be used as one of the approaches to achieve the ultimate goal of elimination of new HIV infections by 2030.

## SEARCH Consortium

Diane Havlir, Moses Kamya, Maya Peterson, Asiphas Owaraganise, Bishop Opira, Cecilia Akatukawasa, Edith Biira, Elijah Kakande, Florence Mwangwa, Geoff Lavoy, Helen Sunday, Jane Kabami, Anjeline Onyango, Collete Inviolata Aoko, Elizabeth Bukusi, Erick Wafula, Greshon Rota, James Ayieko, Janice Litunya, Lawrence Owino, Marilyn Nyabuti, Norton Sang, Sabina Ogachi, Carol Camlin, Catherine Koss, Craig Cohen, Douglas Black, Edwin Charlebois, Eric Goosby, Gabriel Chamie, Jason Johnson Peretz, Jennifer Temple, John Schrom, Judith Hahn, Matthew Hickey, Nicole Sutter, Priscilla Hsue, Starley Shade, Tamara Clark.

## SEARCH Consortium affiliations

University of California, San Francisco, CA, USA (D Havlir MD, C Camlin PhD, C Koss MD, C Cohen MD, D Black BA, E Charlebois PhD, E Goosby MD, G Chamie MD, J Johnson Peretz MAOM, J Temple MSE, J Schrom MPH, J Hahn PhD, M Hickey MD, N Sutter MPH, P Hsue MD, S Shade PhD, T Clark MHS); Makerere University, Makerere, Uganda (M Kamya MBChB PhD); University of California, Berkeley, CA, USA (M Peterson MD PhD); Infectious Diseases Research Collaboration, Uganda (A Owaraganise MBChB, B Opira MPH, C Akatukwasa MPH, E Biira MPH, E Kakande MBChB, F Mwangwa MBChB, G Lavoy, H Sunday MBChB, J Kabami PhD); Kenya Medical Research Institute, Kenya (A Onyango BA, C I Aoko MBChB, E Bukusi MBChB, E Wafula MSc, G Rota BPharm, J Ayieko MBChB PhD, J Litunya MBChB, L Owino BSc, M Nyabuti MBChB, N Sang MA, S Ogachi BSc); University College London, London, UK (A Phillips PhD); University of Pittsburgh, Pittsburgh, PA, USA (U Parikh PhD).
