# Supplementary material for: Dynamic choice HIV prevention with cabotegravir long-acting injectable in rural Uganda and Kenya: a randomised trial extension
Source: Lancet HIV. Author manuscript; Available in PMC 2025 Jan 22. (PMC11753513; doi:10.1016/S2352-3018(24)00235-2)
Supplement: supplement [file NIHMS2043381-supplement-supplement.pdf]

THE LANCET
HIV

Supplementary appendix

This appendix formed part of the original submission and has been peer reviewed. We post it as supplied by the authors.

Supplement to: Kamya MR, Balzer LB, Ayieko J, et al. Dynamic choice HIV prevention with cabotegravir long-acting injectable in rural Uganda and Kenya: a randomised trial extension. *Lancet HIV* 2024; published online Oct 9. https://doi.org/10.1016/S2352-3018(24)00235-2.

SUPPLEMENTARY MATERIALS

TABLE OF CONTENTS

APPENDIX A: PROTOCOL **4**

1. List of Investigators 4

2. List of Abbreviations 7

3. Protocol Summary 8

4. Introduction 9

5. Population 10

6. Hypotheses and Objectives 12

7. Intervention 13

8. Study Procedures 16

9. Informed Consent 20

10. Safety Assesment Overview 21

11. Human Subjects Protections 23

12. Risks 24

13. Social Impact Events 26

14. Benefits 26

15. Compensation 26

16. Participant Privacy and Confidentiality 27

17. Study Discontinuation 27

18. Community Advisory Board, Stakeholders, and Communication of Results 27

19. Administrative Procedures 27

20. Toxicity Management 30

21. Analyses 35

22. Data Handling and Record Keeping 37

23. Publication Policy 38

24. References 38

APPENDIX B: CONSORT 2010 CHECKLIST OF INFORMATION TO INCLUDE WHEN REPORTING A RANDOMISED TRIAL* 43

APPENDIX C: STATISTICAL ANALYSIS PLAN 45

1. Overview of Tthe Search CAB-LA Extension Study 45

2. Population and Characteristics 45

3. Endpoint Definition and Measurement 46

4. Evaluation of the Effect of the Search Dynamic Choice HIV Prevention Intervention with CAB-LA 47

5. Additional Descriptive Analyses 48

6. Sensitivity Analyses 48

7. Power Calculations 49

8. History of Changes 50

9. References 50

APPENDIX D: TABLES AND FIGURES 52

Table S1. Study Dynamic Choice HIV Prevention Intervention and Standard of Care 52

Table S2. Number and Proportion who used CAB-LA at Study Start, Overall and by Baseline Characteristics* 53

Table S3. Prior Product Use on Among Persons Using CAB-LA* 54

Table S4. Sensitivity Analyses for Biomedical HIV Prevention Coverage 55

Table S4a. Results from the primary analysis and the main sensitivity analysis for biomedical HIV prevention coverage, including arm-specific estimates and effect estimates. 55

Table S4b. Additional sensitivity analyses for biomedical HIV prevention coverage, shown as point estimate of the effect with 95% confidence intervals and resulting p-value. 55

Table S5. Characteristics of Study Participants with Incident HIV Infections 57

Table S6. Sensitivity Analyses for Biomedical HIV Prevention Coverage during Periods of Self-Reported HIV Risk 58

Table S6a. Results from the secondary endpoint analysis and the main sensitivity analysis for biomedical HIV prevention coverage during periods of self-reported HIV risk, including arm-specific estimates and effect estimates. 58

Table S4b. Additional sensitivity analyses for biomedical HIV prevention coverage during periods of self-reported HIV risk, shown as point estimate of the effect with 95% confidence intervals and resulting p-value. 58

Figure S1. Study Design 60

Figure S2. Effect of Dynamic Choice HIV Prevention versus Standard of Care on HIV Biomedical Prevention Coverage during Periods of Self-Reported HIV risk* 61

APPENDIX A: PROTOCOL

Extension of SEARCH SAPPHIRE Dynamic Choice Prevention Study

Sustainable East Africa Research in Community Health (SEARCH) Consortium

Clinical Trial Phase: IV

Funded by: National Institutes of Health

Protocol Chair: Diane Havlir, MD

Protocol Co-Chairs: Moses Kamya, MBChB, MPH, PhD
Maya Petersen, MD, PhD

Protocol Vice-Chair: Gabriel Chamie, MD, MPH

Version Number: 3.0

Version Date: 26-July-2023

1. LIST OF INVESTIGATORS

Diane Havlir, MD
Chair, University of California, San Francisco
Address: UCSF Box 0874, San Francisco, CA 94143-0874, U.S.A.
Phone Number: +1-415-476-4082 ext. 424
Email: diane.havlir@ucsf.edu

Moses R. Kamya, MBChB, MMed, PhD
Co-Chair, Makerere University
Address: Department of Medicine, P.O. Box 7072, Kampala, Uganda
Phone Number: +256-414-533200
Email: mkamya@infocom.co.ug

Maya L. Petersen, MD, PhD
Co-Chair, University of California, Berkeley
Address: School of Public Health, 5402 Berkeley Way West, Berkeley, CA 94720-7358, U.S.A.
Phone Number: +1-510-642-0563

Gabriel Chamie, MD, MPH
Vice-Chair, University of California, San Francisco
Address: UCSF Box 0874, San Francisco, CA 94143-0874, U.S.A.
Phone Number: +1-415-476-4082 ext. 445
Email: gabriel.chamie@ucsf.edu

Laura Balzer, PhD
Investigator and Protocol Statistician, University of California, Berkeley
Address: School of Public Health, 5317 Berkeley Way West, Berkeley, CA 94720-7358, U.S.A.
Phone Number: +1-203-558-3804
Email: laura.balzer@berkeley.edu

Andrew Phillips, MSc, PhD
Investigator, Modeling, University College London, London
Address: Institute for Global Health, Glower Street, WC1 6BT, U.K.
Phone Number: +20-7830-2886
Email: andrew.phillips@ucl.ac.uk

Florence Mwangwa, MBChB, MPH
Investigator, Adult and Adolescents Studies, Infectious Diseases Research Collaboration
Address: P.O. Box 4745, Kampala, Uganda
Phone Number: +256-779-386616
Email: fmwangwa@idrc-uganda.org

Jane Kabami, MPH
Investigator, Infectious Diseases Research Collaboration
Address: P.O. Box 7475, Kampala, Uganda
Phone Number: +256-706-315810
Email: jkabami@idrc-uganda.org

Elijah Kakande, MBChB
Investigator, Infectious Diseases Research Collaboration
Address: P.O. Box 4745, Kampala, Uganda
Phone Number: +256-779-386616
Email: rkakande@idrc-uganda.org

Asiphas Owaraganise, MBChB
Investigator, Infectious Diseases Research Collaboration
Address: P.O. Box 4745, Kampala, Uganda
Email: asiphas@gmail.com

Elizabeth Bukusi, MBChB, M.Med, MPH, PhD
Investigator, Youth and Integration of HIV and Reproductive Health Services, Kenya Medical Research Institute
Address: Box 54840, Nairobi, Kenya
Phone Number: +254-733-617503
Email: ebukusi@rctp.or.ke

James Ayieko, MBChB, MPH, PhD
Investigator, Adult and Adolescent Studies, Kenya Medical Research Institute
Address: P.O. Box 517-20107, Njoro, Kenya
Phone Number: +254-720-925262
Email: jimayieko@gmail.com

Norton Sang, MA
Investigator, Kenya Medical Research Institute
Address: P.O. Box 35-4132, Mega City, Kisumu, Kenya
Phone Number: +254-715-778930
Email: nortonsang@gmail.com

Marilyn Nyabuti, MBChB
Investigator, Kenya Medical Research Institute and the University of Washington, Seattle
Address: P.O. Box 35-4132, Mega City, Kisumu, Kenya
Phone Number: +254-715-778930
Email: mnyanduko@gmail.com

Judith Hahn, PhD, MA
Investigator, Alcohol Use Studies, University of California, San Francisco

Address: UCSF Box 1224, San Francisco, CA 94158
Phone: +1-415-476-5815
Email: judy.hahn@ucsf.edu

Carol Camlin, PhD, MPH
Investigator, Social Sciences/Qualitative Studies, University of California, San Francisco
Address: Center for AIDS Prevention Studies, San Francisco, CA 94143, U.S.A.
Phone Number: +1-415-597-4998
Email: carol.camlin@ucsf.edu

Starley Shade, PhD
Investigator, Cost Effectiveness, University of California, San Francisco
Address: Box 0886, San Francisco, CA 94143-0886, U.S.A.
Phone Number: +1-415-476-6362
Email: starley.shade@ucsf.edu

Harsha Thirumurthy, PhD
Investigator, Behavioral Economics Studies, University of Pennsylvania
Address: Department of Medical Ethics and Health Policy, Philadelphia, PA 19104
Phone Number: +1-215-746-0410
Email: hthirumu@upenn.edu

Edwin Charlebois, PhD, MPH
Investigator, University of California, San Francisco
Address: UCSF Box 0874, San Francisco, CA 94143-0874, U.S.A
Phone Number: +1-415-476-6241
Email: edwin.charlebois@ucsf.edu

Priscilla Hsue
Investigator, Cardiovascular Studies, University of California, San Francisco
Address: UCSF Box 0846, San Francisco, CA 94110
Phone: +1+415-206-8257
Email: Priscilla.hsue@ucsf.edu

Theodore Ruel, MD
Investigator, HIV Adolescent and Young Adult Studies, University of California, San Francisco
Address: UCSF Box 0434, San Francisco, CA 94143-0136, U.S.A.
Phone Number: +1-415-476-9197
Email: theodore.ruel@ucsf.edu

Craig Cohen, MD, MPH
Investigator, University of California, San Francisco
Address: Reproductive Sciences, Box 1224., San Francisco, CA 94158 - youth and integration of HIV and reproductive health services
Phone Number: +1-415-476-5874
Email: craig.cohen@ucsf.edu

Eric Goosby, MD
Investigator, University of California, San Francisco
Address: UCSF Box 0874, San Francisco, CA 94143-0874, U.S.A.
Phone Number: +1-415-476-5483
Email: eric.goosby@ucsf.edu

Catherine Koss, MD
Investigator, University of California, San Francisco
Address: UCSF Box 0874, San Francisco, CA 94143-0874, U.S.A.

Phone Number: +1-415-476-4082
Email: catherine.koss@ucsf.edu

Urvi M Parikh, PhD
Virologist, University of Pittsburgh, Pittsburgh
Address: S830 Scaife Hall, 3550 Terrace Street, Pittsburgh, PA 15261
Phone Number: 412-648-3103
Email: ump3@pitt.edu

Bishop Opira, Bpharm, MPH
Pharmacist, Infectious Diseases Research Collaboration, Kampala, Uganda
Address: Plot 2C Nakasero Hill Road,P.O. Box 7475 Kampala, Uganda
Phone: +256 (0)312 281 479
Email: opibish@idrc-uganda.org

Greshon Rota, Bpharm
Pharmacist, Kenya Medical Research Institute
Address:P.O. Box 54840 00200 Off Raila Odinga Way. Nairobi, Kenya.
Phone: +254 722205901
Email: director@kemri.go.ke

2. LIST OF ABBREVIATIONS

AIDS: Acquired immunodeficiency syndrome
ART: Antiretroviral therapy
CAB-LA: Long-Acting Cabotegravir
CE: Cost-effectiveness
CHR: Committee on Human Research
CHV: Community health volunteer
CRF: Case report form
CVD: Cardiovascular disease
DALYs: Disability-adjusted life years
DM: Diabetes mellitus
DSMB: Date Safety Monitoring Board
ELA: Empowerment and Livelihood for Adolescents
FTE: Full-time equivalent
FGD: Focus group discussion
GPS: Global positioning system
HIV: Human immunodeficiency virus
HSV: Herpes simplex virus
HTN: Hypertension
IDI: In-depth interview
IPV: Intimate partner violence
IRB: Institutional Review Board
KEMRI: Kenya Medical Research Institute
MoH: Ministry of Health
MTCT: Mother-to-child HIV Transmission
NASCOP: National AIDS and STI Control Programme
PEP: Post-exposure prophylaxis
PEth: Phosphatidylethanol
PRECEDE: Predisposing, Reinforcing, and Enabling Constructs in Educational Diagnosis and Evaluation
PrEP: Pre-exposure prophylaxis
SAP: Statistical analysis plan
RE-AIM: Reach, Effectiveness, Adoption, Implementation and Maintenance
SEARCH: Sustainable East Africa Research in Community Health

SOMREC: Makerere University School of Medicine - Research and Ethics Committee
SOPs: Standard Operating Procedures
SSA: Sub-Saharan Africa
STI: Sexually-transmitted infections
TB: Tuberculosis
TDF/FTC: Tenofovir disoproxil fumarate/Emtricitabine
TMLE: Targeted maximum likelihood estimation
TFV: Tenofovir analyte
UCSF: University of California, San Francisco
UNCST: Uganda National Council for Science and Technology
VMMC: Voluntary medical male circumcision
VHT: Village health team. This term (Uganda) used in protocol refers to equivalent CHV (community health volunteer)

3. PROTOCOL SUMMARY

Title: Extension of SEARCH SAPPHIRE Dynamic Choice Prevention Study

The Extension of SEARCH SAPPHIRE Dynamic Choice Prevention Study is a continuation of the 3 ongoing SEARCH SAPPHIRE Dynamic Choice Prevention randomized trials. The parent SEARCH SAPPHIRE Dynamic Prevention intervention studies were initiated in 2021, and were testing the hypothesis that a patient centered HIV Dynamic choice intervention would increase HIV biomedical prevention time in persons at risk for HIV infection. The trial is being conducted in 3 settings; outpatient clinics, antenatal/family planning clinics, and via VHT workers in community households. The dynamic choice intervention includes counseling on and services of PrEP/PEP, choice of service location (e.g. clinic, household, community, phone), HIV testing options (including rapid or self-test provision), option for longer (e.g. 3 month) supply of PrEP, provision of a clinical officer's or nurse's mobile telephone number for immediate PEP starts any day of the week, routine assessment of barriers to initiation or adherence to PrEP/PEP, including the offer of personalized potential solutions such as choice of in-clinic or offsite service delivery, psychologic supports for traumatic experiences, and offer of concurrent, additional health or prevention related services. These services are integrated into Uganda and Kenya Ministry of Health (MoH) guidelines driven services for PrEP/PEP, augmenting local standard care procedures for the prevention of HIV. In both arms, participants have access to standard prevention offerings and will have study visits every 24 weeks for up to 3 years for HIV testing and outcomes measurement (including PrEP/PEP adherence assessment via small hair sample).

This extension protocol is a continuation of the 3 Dynamic Prevention Randomized studies for a 48 week follow up period following re-consent, with the addition of Cabotegravir (CAB-LA) as one of the dynamic choice biomedical options for intervention participants. CAB-LA is a new prevention intervention that requires injections every 8-weeks, rather than daily pill intake. CAB-LA offers an option for HIV prevention that does not require daily pills. In two head-to-head comparison studies that randomized persons to either CAB-LA or Tenofovir daily, HIV infections were reduced by over 50% in the CAB-LA arm. CAB-LA was approved in the United States in December 2021; while it is not yet approved in Uganda or Kenya, applications have been submitted and approval is expected in the coming year.

In this DCP CAB-LA Extension, persons who are currently enrolled in the DCP trials and were randomized to the intervention arm will be eligible to receive CAB-LA as one of the options in the "Dynamic prevention" model. Persons in the control arm will be eligible to receive CAB-LA if and when it is rolled out through in-country programs. Upon study approval, all participants will be offered re-consenting to continue on this study.

If CAB-LA is not available in country at the 48-week endpoint, all participants in the intervention arm will have the opportunity to continue in the study until Week 96, and participants who are currently on CAB-LA may continue through the extension. The study will end at Week 96, and by that time participants will be transitioned to in country prevention care. At 96 weeks, persons on CAB-LA will be transitioned to in country CAB-LA or to other prevention options if CAB-LA is not available. Prior to this transition, available in country options will be assessed by the study team. HIV prevention options will be discussed with each study participant by their Week 88 visit, with a plan established for the transition to the participant's preferred prevention method in coordination with local health facilities to avoid interruption after their 96 weeks of study participation is completed.

Participants in the control arm can access biomedical HIV prevention interventions (oral PrEP, PEP) through the local health clinics. They will have access to CAB LA when it becomes available through the Ministry of Health.

Participants in the intervention arm with have access to PrEP, PEP + CAB LA with the additional structured dynamic choice support described above. Intervention participants that choose not to use CAB-LA will continue on a quarterly study visit schedule, per the standard DCP Intervention study schedule. Intervention participants that choose CAB-LA will be seen once every 4 weeks for the first two injections, then every 8-weeks for injections and study visits. Should participants stop CAB-LA, they will revert to the quarterly DCP visit schedule. All participants will be seen for mid-point and end-point visits every 24 weeks.

A subset of participants that use CAB-LA (up to 40) will be asked to complete an in-depth-interview and all participants will take a survey at Weeks 0, 24, and 48 (or at CAB-LA discontinuation) for researchers to better understand participant's experience with CAB-LA and to improve delivery as it becomes more widely available. A subset of 10 providers will also be asked to complete an in-depth interview and all providers will complete surveys at their Weeks 0, 24, and 48 of administering CAB-LA. These interviews and surveys will help researchers understand better ways to deliver CAB-LA, as it becomes more widely available. Participants that seroconvert (acquire HIV during the trial) will also be asked to participate in an in-depth interview, as it will help researchers understand transmission and ways to improve DCP delivery in future implementation.

There is limited safety information available about CAB-LA use during pregnancy or breastfeeding. In the HPTN 084 trial (Phase 3 safety and efficacy trial of injectable cabotegravir compared with TDF-FTC for HIV prevention in HIV-uninfected women), the 63 women who became pregnant while taking CAB-LA had no birth defects. Given this precedent, participants will not be allowed to begin CAB-LA while pregnant, but may continue CAB-LA if they become pregnant or are breastfeeding. Participants will be explained the risks and benefits of CAB-LA during pregnancy or breastfeeding prior to enrolling in the study. Participants that become pregnant will be provided detailed information on the risks and benefits of continuing CAB LA and asked to sign a separate consent form, asking that the study team follow her, for informational purposes only, for up to 8 weeks post-partum to ascertain the outcome of the pregnancy The pregnancy follow-up is for health monitoring of the mother and infant, as well as contributing to the global knowledge base of pregnancy and CAB-LA use.

The overall purpose of this extension is to 1) Determine if adding the option of CAB-LA as a prevention choice using a patient-centered HIV prevention delivery model increases prevention coverage (i.e. proportion of time that is covered by a biomedical prevention option, such as PrEP or PEP) compared to the standard-of-care in 3 ongoing randomized trials of Dynamic Choice Prevention in rural Uganda and Kenya; and, 2) Conduct a hybrid implementation study (Primary Objective Clinical and Secondary Objective Implementation) focusing on initial implementation of a patient-centered model for CAB-LA using the RE-AIM evaluation framework among persons randomized to the intervention arm

4. INTRODUCTION

4.1. Background

SEARCH SAPPHIRE is an ongoing, NIH-funded study that is testing multi-disease and multi-sector interventions aimed at reducing HIV burden and improving health in rural Uganda and Kenya. Phase A is a portfolio of randomized prevention and treatment implementation studies that will inform a population-level study in Phase B. **HIV incidence in Sub-Saharan Africa (SSA) remains well above global elimination targets.[1]** Despite an increasingly large, evidence-based toolkit to prevent HIV transmission, there were over 1 million new HIV infections in SSA in 2018.[1] We know from SEARCH[2] and three other recent SSA population-level studies[3-5] that reaching (and exceeding) *the UNAIDS 2020 population-level viral suppression target of 73%[6] is insufficient to achieve epidemic control* (**Table 1**). We also know that key sub-populations, in particular youth,[4, 7-10] men,[2, 4, 10-12] mobile populations,[13-15] and persons with heavy alcohol use[16, 17] continue to fall short of viral suppression targets, putting them at higher risk for HIV complications, mortality, and onward HIV transmission. Finally, pre-exposure prophylaxis (PrEP), an intervention that has reduced incidence in affected populations in some areas where high rates of viral suppression were already present,[18, 19] has not been effectively deployed in high risk populations (such as young women) in SSA.[20, 21] To achieve control of the HIV epidemic and mitigate its consequences, we need **new and scalable** interventions to improve delivery of prevention and treatment to **vital segments of the population that contribute to persistence of new infections.** We describe below why previous approaches that were effective for the majority of people in the ART roll-out do not work for subsets of these "persistent-driver" populations, our proposed solutions, and how a multi-component intervention trial can effectively inform stakeholders on programming and resource allocation to accelerate reductions in HIV incidence.

HIV prevention services that offer choice of delivery options (PrEP, CAB-LA, & PEP) and flexibility in their use and location of delivery over time allow for a dynamic, patient-centered model of prevention. This approach is responsive to the reality of an individual's changing needs, thereby facilitating continued engagement. In SEARCH, PrEP use declined over time in spite of initial enthusiasm and uptake by early adopters[22]. In multiple settings, poor adherence to PrEP remains its Achilles' heel.[23,24] Rather than presenting PrEP in a similar framework to ART (i.e. lifelong use with 100% adherence for efficacy with delivery at HIV clinic), approaches that incorporate flexibility in choice and delivery location, akin to contraception options for women in developed countries, may prove more successful. Indeed, over the past four decades, increased choice in contraception and flexibility in access have been directly associated with increased use.[25,26] Framing prevention in the context of healthy choices, rather than an assessment of risk, may also reduce the association of PrEP with "risky" behavior or promiscuity. Offering PEP – an event-driven option that does not require accurate prediction of risk before a potential HIV exposure (akin to a "morning after" pill), and which additionally provides protection for those with less sexual agency – is a critical innovation in flexible access to prevention services. PEP can also act as a "gateway" to PrEP or CAB-LA and other prevention modalities, providing infrastructure to add new prevention options as they become available (e.g. the PrEP vaginal ring[27,28]).

In the HPTN 084 superiority trial there was an 88% reduction in HIV infections in the CAB-LA arm compared to TDF/FTC, with a study population (3,224 participants) of cisgender women, aged 18-45, in Sub-Saharan Africa, including Uganda. Amongst participants that became pregnant, no congenital abnormalities were reported, with more data on pregnancy and infant outcomes expected from the HPTN 084 open-label study.

This extension describes a 48-week extension of 3 ongoing randomized trials in SEARCH Phase A studying a patient-centered "Dynamic Choice Prevention" (DCP) implementation strategy for delivering existing evidence-based biomedical prevention interventions vs. standard of care (SOC). The Dynamic prevention model offers participants choices on prevention modality on an ongoing basis: oral PrEP, or oral PEP, and the option to switch between products. The trials are being conducted in 3 settings: antenatal (ANC) clinics, the outpatient department (primary care clinics), and in rural community settings in Western Uganda and Kenya. All clinic and village health team staff (i.e., Clinical Officers, nurses, coordinators, health workers) are trained and equipped for HIV prevention care in the clinical setting, appropriate to their role.

In this extension study, participants in the intervention and control arms are reconsented and remain in their initial randomized arm. Persons in the intervention arms who remain HIV negative will be offered CAB-LA as an additional biomedical prevention option, if eligible. CAB-LA will be delivered at health clinics. The primary 48-week endpoint for this extension is proportion of time covered by a biomedical prevention product. As of May 9, 2022, a total of 1213 HIV-negative persons aged 15 years and older and at risk for HIV acquisition were enrolled in the studies. (Table 1). New participants are currently being enrolled in the original SEARCH SAPPHIRE DCP trials to replace those participants who have moved or have been unable to continue in the DCP study. The total number of participants in the study eligible for CAB-LA will not exceed the 593 listed in Table 1.

Table 1. Enrollment in 3 SEARCH Sapphire Dynamic Prevention Trials as of May 9, 2022

Dynamic Prevention Study	Intervention and Control, (N) Uganda	Intervention (N) Uganda	Intervention and Control, (N) Kenya	Intervention (N) Kenya	Total Enrolled (N), Intervention and Control	Total Enrolled Intervention (N)
Antenatal/SRH Clinic	200	100	201	103	401	203
Outpatient Department Clinics	201	100	201	97	402	197
Household Study Community Village Health Team (VHT)	200	83	210	110	410	193
All Trials Together	601	283	612	310	1213	593

5. POPULATION

5.1. Recruitment

The persons eligible for participation in the extension are those who were enrolled in the 3 ongoing DCP trials. Persons for the ANC study are recruited and enrolled through offering study participation at ANC clinics at government sponsored health facilities. Persons for the Outpatient department are recruited and enrolled through

offering study participation at Outpatient department clinics at government sponsored health facilities. Persons for the community study are recruited via home visits by village health teams/community health workers.

5.2. Inclusion and Exclusion Criteria

Inclusion criteria for the Extension include:
1) Enrollment in a SEARCH Sapphire Dynamic Choice Prevention study
2) HIV negative at start of extension
3) Residing in study region

Additional inclusion criteria to access CAB-LA as a prevention option:
1) Not pregnant at time of initial CAB-LA injection
2) Participant weighs at least 35 kg

Additional Exclusion Criteria to access CAB-LA as a prevention option:
1) Participant has Hepatitis B or chronic Hepatitis C Diagnosis
2) Participant has ALT >=5x ULN
3) Participant has clinical history of liver cirrhosis or current clinical evidence of cirrhosis or severe liver disease
4) Previous hypersensitivity reaction to cabotegravir
5) Receiving the following co-administered drugs for which significant decreases in cabotegravir plasma concentrations may occur due to uridine diphosphate glucuronosyltransferase:
   i. Anticonvulsants: Carbamazepine, oxcarbazepine, phenobarbital, phenytoin
   ii. Antimycobacterials: Rifampin, rifapentine
6) Participants with a current or anticipated need for chronic systemic anticoagulation or a history of known or suspected bleeding disorder, including a history of prolonged bleeding, except for the use of anticoagulation for deep vein thrombosis (DVT) prophylaxis (e.g., postoperative DVT prophylaxis) or the use of low dose acetylsalicylic acid (≤325 mg).

Study Intervention Clinic Locations:

All participating ANC and OPD clinics in the DCP trial will offer CAB-LA. For VHT/CHV communities, the nearest health facility will offer CAB-LA for all participants:
1) In Uganda, the sites for each of the trials:
   a. OPD: Itojo Hospital, Kitagata Hospital
   b. ANC: Bwizibwera HCIV, Bushenyi HCIV
   c. VHT: Ndeija Health Center III
2) In Kenya, the sites for each of the trials:
   a. OPD: Magunga Level IV Hospital, Oyani Hospital
   b. ANC: Oyani Hospital, Sena Health Centre
   c. CHV: Sibouche Health Centre, Ogongo Sub-District Hospital

The demographic characteristics of the persons enrolled in the intervention arm of the DCP Trials as of May 9, 2022 currently who will be eligible for CAB-LA patient-centered intervention are shown in Table 2.

Table 2. Demographics of persons in intervention arm eligible for CAB-LA in extension phase of trial as of May 9, 2022

	ANC Clinic	Outpatient Department	Community
Total Enrolled	203	197	193
Female Participants	203 (100%)	119 (60.4%)	111 (57.2%)
Age years number and %			
15-25 years	120 (56.9%)	90 (44.1%)	75 (38.9%)
26- 30	47 (22.3%)	33 (25.0%)	29 (25.4%)
31-40	35 (16.6%)	51 (16.2%)	49 (18.7%)

41-60	9 (4.3%)	26 (12.7%)	36 (15.0%)
61+	0 (0%)	4 (2.0%)	4 (2.1%)

Rationale: HIV prevention services that offer choice of prevention products (PrEP & PEP) and flexibility in their use and location of delivery over time allow for a dynamic, patient-centered model of prevention. This approach is responsive to the reality of an individual's changing needs, thereby facilitating continued engagement. CAB-LA is highly efficacious for HIV prevention that was recently approved by the US FDA based on two large, randomized trials HPTN 083 and HPTN 084. As CAB-LA is rolled out across rural sub-Saharan Africa, it is important to determine **how the addition of this option impacts prevention coverage in real-world settings** for women and men at risk for HIV. In addition, it is critical to design, evaluate, and improve **patient-centered CAB-LA delivery** strategies that maximize uptake and impact of this new product.

Ethical Considerations: The HPTN 084 study that showed efficacy of CAB LA for prevention included sites in both Uganda and Kenya. Applications for approval of CAB LA are under review in each of these countries.
Thus, the information generated in this study on evaluating a dynamic prevention implementation strategy will be directly applicable. *During this proposed study extension, CAB LA is expected to become publicly available through Ministry of Health ("MoH") sponsored programs in both Kenya and Uganda. UCSF is developing an additional extension to 96 weeks, which will examine longer-term safety and implementation data of CAB-LA. Should there be delay in rollout of the MoH programs, participants could continue in this additional extension phase. After the endpoint of the 96-week extension is reached, participants will be transitioned to a standard of care regimen, via local healthcare system, based on the investigator's clinical judgment and local country guidelines.*

6. HYPOTHESES AND OBJECTIVES

6.1. Hypotheses

A dynamic choice prevention model that includes a patient-entered CAB-LA delivery option will have higher levels of biomedical prevention coverage (assessed over 48 weeks following re-consent for the CAB-LA extension study) compared to the standard of care.
This hypothesis will be tested by comparing covered time between the intervention and control arms in the 3 ongoing randomized trials during the extension phase.

The secondary hypothesis: Addition of patient-centered CAB-LA into an ongoing dynamic choice prevention will improve biomedical prevention coverage (compared to dynamic choice prevention without CAB-LA).
These hypotheses will be tested by comparing covered time during the extension with the CAB-LA option to covered time prior to the extension among intervention arm participants.

We expect that incorporating the option of CAB-LA with patient-centered delivery for persons who self-identify at risk for HIV living in rural Uganda and Kenya will increase overall biomedical covered prevention time compared to the standard of care. In a population that has been offered -- and some of whom have taken up -- oral PrEP and PEP, we also expect to learn characteristics of who is most interested in CAB-LA, how our patient-centered CAB-LA delivery models work in different settings, and the cost of the delivery models in different settings.

6.2. Objectives

Primary Objective: To compare biomedical prevention coverage achieved using a Dynamic prevention model that includes a patient-centered CAB-LA delivery intervention to biomedical prevention coverage under the standard of care over 48 weeks.
Secondary Objectives: To determine the reach, effectiveness, adoption, implementation and maintenance of a patient-centered CAB-LA program embedded in 3 ongoing trials in the setting of antenatal clinic, outpatient clinic, and community.
Tertiary Objectives: To evaluate change in knowledge, awareness and acceptability/satisfaction at the staff and provider level with CAB-LA before and after provider and staff training and education in CAB-LA with patient-centered delivery model.

7. INTERVENTION

7.1. Study Design and Schema

Study Design: This proposal is an **extension of 3 ongoing randomized trials comparing a patient-centered "Dynamic Choice" implementation strategy for delivering biomedical prevention options compared to standard of care** (Figure 1). Each trial is enrolling persons 15 years of age and older who are HIV uninfected and at risk of acquiring HIV. The Dynamic Choice Prevention intervention allows choice of PEP/PrEP, refill schedule, service delivery location (e.g. clinic, community, home), and HIV testing (self or staff). Persons can move from PEP to PrEP as they prefer.

Figure 1. Dynamic Choice Implementation Strategy.

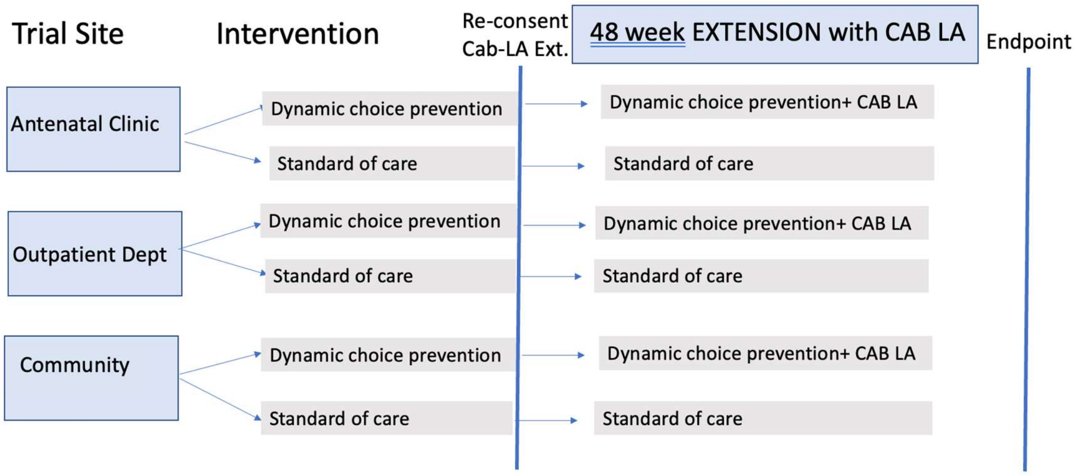

Intervention: In the extension phase, participants will remain in their initial randomization arm. For persons in the intervention arm, CAB-LA will be integrated into the dynamic choice delivery model as an additional biomedical prevention option in a patient-centered delivery model based on the precede framework.

7.2. Dynamic Choice Delivery Model

The Dynamic Choice Delivery Model includes integrated PrEP and PEP services at outpatient clinics, antenatal clinics, and via VHT workers in community households. The procedures for each trial include PrEP/PEP counseling and services, choice of service location, HIV testing options, option for longer PrEP refills, provision of a clinical officer's or nurse's mobile telephone number for immediate PEP starts any day of the week, assessment of PrEP/PEP barriers and personalized actions, psychologic supports for traumatic experiences, and offer of concurrent, additional health or prevention related services. For participants that chose CAB-LA as the biomedical prevention option, the patient-centered delivery model (Table 3) will be implemented. The standard of care differs according to country but does not routinely offer PEP or PrEP to clients seeking services, does not offer choice of service location, HIV testing option or access to medical provider mobile phone number. Clients are required to return after one month for a refill for PrEP, whereas in our model, there is an option from the start for 3 month medication supply.

Table 3. Patient-Centered Delivery Model

Intervention	Population and frequency	Purpose
**Education, case studies and discussion** on concept of dynamic prevention and on the profile of each prevention option product	Health center leadership and staff, provider, participants; initial and ongoing	Predisposing

**Integration** into antenatal, OPD clinics, **IPV/GBV support services, travel packs, 24 hour hotline for participants**<br>For oral PrEP/PEP only- HIV testing choice (self or staff) and home or clinic delivery	Participants; ongoing	Enabling
**Provider text/check-in to participant** one week after starting new option, **data on biomedical prevention covered time** (reinforcing)	Participants and staff; ongoing data feedback	Reinforcing

7.3. Measurement

Outcomes: The primary outcome for the extension is the proportion of time covered by a biomedical prevention option during the 48 week follow-up period of the CAB-LA Extension; follow-up time will be censored at HIV diagnosis, death, or withdrawal. Biomedical covered time is defined as the proportion of follow-up time that each participant is covered by either PrEP (TDF/3TC or CAB-LA) or PEP. For TDF/3TC, covered time is defined using self-reported use; in a subset of participants who report PrEP use we will measure tenofovir drug levels using small hair samples to allow for calibration of self-reported measures. For CAB-LA, coverage begins 3 days after initial injection continues 2 months + 7 days after last injection. For PEP, covered time is defined using self-reported use; in a subset of participants who report PEP use we will measure drug levels using hair samples to allow for calibration of self-reported measures. A secondary outcome for the extension is "at-risk covered time", defined as the proportion of time that a participant reports being at risk of HIV infection during which they are covered by a biomedical prevention option. Incident HIV infections, defined by a prespecified testing algorithm, will be an additional secondary outcome.
Secondary outcomes of reach, effectiveness, adoption, implementation and maintenance will be evaluated using a mixed methods approach that includes surveys of participants, and health center providers and staff. Tertiary outcomes of knowledge, awareness and acceptability/satisfaction at the staff and provider level will be measured by surveys at the baseline, midpoint and end of the study. Measurement details are delineated in Table 4 .

7.3.1. BEHAVIORAL MEASURES

In addition to these surveys, trained staff will conduct in depth interviews (IDI) to provide insights into the intervention implementation and attitudes of participants and staff. These qualitative in-depth interviews will be conducted among purposively-selected samples of study participants and providers, to ascertain the perceptions of and experiences with CAB-LA use and delivery. Participants who are clients will be asked about 1) Experiences with counseling, method/service preferences and decision-making to go on CAB-LA; 2) Method/services satisfaction, barriers and facilitators to uptake and maintenance, and social networks and support as a facilitator or hindrance; and 3) Recommendations to improve CAB-LA Delivery. Health care providers engaged in CAB-LA service delivery will be asked about their 1) Professional identity and motivation, as a potential factor influencing implementation; 2) First impressions and knowledge of CAB-LA; 3) Clinical training experiences relevant to CAB-LA provision; 4) Experiences with and acceptability of individual elements of CAB-LA; and 5) Recommendations for improving CAB-LA delivery.

For all research activities related to qualitative evaluation of SEARCH SAPPHIRE, written informed consent will be obtained from all selected for recruitment to the study.

Qualitative IDIs for CAB-LA will be conducted between 2-6 months from enrolment. The specific data collection activities to be conducted during the phase are listed below. For Seroconverts, IDIs are will continue until the maximum number of participants is reached or the study intervention ends, whichever comes first.

i. CAB-LA Patient IDI cohort: We will systematically select ~n=30 patients per each region (South West Uganda and Kenya) (total 40 < ~n < 72), with samples within region to be balanced by original trials (Out- patient, ANC/PNC, VHT); further balance to be observed by gender and community, proportional to overall intervention enrolment.
ii. CAB-LA Provider IDI cohort: We will select a sample of care providers who administer CAB-LA from the CAB-LA trial health facilities or who inform the community about that option (VHTs / CHVs per each region. I.e. n=2-3 OPD providers, n=2-3 ANC/PNC providers, n-1-2 VHTs per region (total 8 < ~n < 12 providers per region).

iii. Seroconverts Patient IDI cohort: We aim to interview all participants in the intervention arm who received a diagnosis of HIV during the course of the DCP study and who are willing to be interviewed, up to a total of 36 per country.

For this study, IDIs will be recorded using the TASCAM DR-05 digital recorder. This digital recording device was selected for use in this study because it has a high quality microphone that captures high quality sound even in settings with a lot of ambient background noise.

For this study, the interviews will be conducted in the participant's preferred language. The Qualitative study team will then transcribe and translate the audio recorded data into English for analysis within 14 days of the interview. Staff will use either Express Scribe or VideoLAN Client (VLC) software for this process. QRCs/QRAs will share responsibility for coding all transcriptions of recorded IDIs. Coding will be conducted in Dedoose using a codebook developed iteratively by the team. Analysis will follow a modified framework analysis of key code excerpts exported from Dedoose after coding.

7.3.2. COSTING EVALUATIONS

Costing of CAB-LA implementation will be conducted in each region and location type (Antenatal Clinic, Outpatient Department, Community) at Baseline/Screening and at two injection visits during follow-up. Costing exercises will employ an activity-based approach and include site visits and employ rigorous costing methods using standardized micro-costing tools. We will also conduct time-and-motion activities using self-administered tools with on-site staff to estimate the amount of time spent implementing CAB-LA. We will interview study staff to identify resources used to implement the intervention and estimate time spent providing oversight and management of implementation activities.

Implementation Research Design: The secondary objective of the extension is an implementation evaluation of the intervention focused on the initial implementation of patient-centered CAB-LA using the RE-AIM framework. The domains of interest and key outcomes are summarized in the table below. Throughout, study weeks are used to refer to weeks in CAB-LA extension (eg, "week 0" is date of re-consent for the CAB-LA extension, "week 48" is 48 weeks after re-consent for the CAB-LA extension).

Table 4. RE-AIM Evaluation Framework

Domain /Outcome	Definition	Measurement	Timing
**Reach**<br>(Participant level)	Proportion of eligible participants who initiate CAB-LA weeks 0-48.<br>Characteristics (including prior PrEP and PEP use) and attitudes (knowledge, acceptability and feasibility) of participants using CAB-LA	Demographics, CAB-LA dispensing logs<br>Knowledge, Acceptability and Feasibility (Survey and IDI). Evaluation includes focus on equitable access by gender, education and SES.	CAB LA dispensing throughout follow-up<br>Surveys and IDI: Week 0, 24 and week 48
**Effectiveness**<br>(Participant level)	Impact of adding CAB-LA to Dynamic choice prevention on biomedical covered time<br>(comparison of follow-up during the CAB-LA extension vs. follow-up time in Phase A trials prior to CAB-LA extension).	CAB-LA dispensing logs, PrEP and PEP use as defined for primary endpoint in extension.	Throughout follow-up
**Adoption**<br>(Provider and staff level in different settings)	% of staff and providers invited that participate in the interventions, where participation is defined as<br>i) participation in CAB-LA trainings; and ii) offering CAB-LA to participants. We will further assess the characteristics of participant vs. non-participant staff.	Training attendance rosters; CAB-LA dispensing logs	Throughout follow-up
**Implementation**<br>(Provider and systems level)	Fidelity: Proportion of providers offering CAB-LA on appropriate schedule, appropriate provision of oral bridging<br>Costing: Incremental cost of adding CAB-LA to dynamic choice prevention	CAB-LA and oral bridging dispensing logs<br>Time in motion and program costing<br>Adaptation logs	Throughout follow-up

	Adaptations: Type, timing and reasons they were made		
**Maintenance** (adapted from standard RE-AIM to accommodate shorter follow-up) (Participant level)	Persistence on CAB-LA among participants who initiate CAB-LA<br>Reasons for discontinuation of biomedical prevention or switching to other biomedical prevention option	CAB-LA dispensing logs<br>Participant survey on reasons for discontinuation and switching	Throughout follow up

As a tertiary objective, we will also assess provider and staff Knowledge, Acceptability and Awareness via provider and staff surveys (Likert scales) and IDI at weeks 0, 24, and 48.

8. STUDY PROCEDURES

8.1. Schedule of Evaluations

For all participants, from the time of reconsenting at the start of study CAB-LA availability (Week 0), there will be study visits every 24 weeks for the duration of their participation.
At the time of writing of this protocol version, the standard of care for both Kenya and Uganda is to offer biomedical prevention as part of the HIV prevention package that includes provision of PrEP for persons at risk of HIV acquisition, such as persons with partners living with HIV or of unknown HIV status, or those with a history of exchange/transactional sex, recent STIs, or recurrent use of PEP (more than 3 times a year). PEP is offered primarily after occupational exposures or in cases of sexual assault. For participants consenting in the extension, in the control arm, they will continue to be seen by government staff in the HIV clinic or other service location, like a drop-in centre, and receive prevention interventions according to standard of care guidelines recommended by the country's Ministry of Health. There will be no change to their HIV prevention healthcare.
Control participants will be seen for a study visit every 24 weeks for HIV testing (RNA and rapid), weight, and a survey on which prevention method(s) they have been using and self-reported risk. If control participants report using medications such as PrEP or PEP at the interim visits, they will be asked to provide hair, blood, or urine samples in order to measure HIV prevention medication levels.
For intervention participants who do not choose CAB-LA, there will be study visits every 12 weeks; however, they can decide to begin CAB-LA at any time during the initial 48-week extension. Participants will not be able to initiate or restart CAB-LA in the additional extension period between Week 48 and Week 96; only those who are taking CAB-LA at the time of the Week 48 visit may continue. Intervention participants that choose CAB-LA will have study visits that align with their injection visits (every 4 weeks for the first two months and then every 8 weeks thereafter), but can return to an alternate DCP intervention if they decide to stop CAB-LA.
For participants that select CAB-LA, the extension visits will also include (Table 6):

- A separate qualitative interview (At time of CAB-LA start, Week 24 and Week 48)
- Safety Labs:
  - HBsAg at baseline
  - ALT at Weeks 0 and 24; optionally at Weeks 8 and 48
- Pregnancy tests
- In addition to the Week 24 and 48 extension visits, injection visits will occur in clinic every 4 weeks for the first two months and then every 8 weeks thereafter
- HIV-RNA Cepheid test at baseline, Week 24, Week 48, Week 72, Week 96
- Plasma will be stored at each visit for future use to evaluate use of alternate HIV assays and cabotegravir PK. Small hair samples will be stored at visit weeks 8, 24, 32, and 48 to assess cabotegravir PK.
- Participants that stop CAB LA prior to the end of the extension time period will continue to be followed in the study but *not* be actively followed beyond the 96 week followup time of the study.
  - Oral PrEP (TDF/3TC) will be recommended to cover tail
  - Participants will switch back to Q12 week follow-up

Table 5. Schedule of Evaluations Dynamic Choice Prevention Extension – Baseline to Week 48

	Screening/ Baseline	Week 12	Week 24	Week 36	Week 48[4]
**DCP Intervention (For persons not receiving CAB-LA)[1]**					
Study visit	X	X	X	X	X
DCP intervention, including:	X	X	X	X	X
- Offer PrEP, PEP, prevention options[2]	X	X	X	X	
- HIV testing options	X	X	X	X	X
- Visit location choice	X	X	X	X	X
- Structured assessment of barriers	X	X	X	X	X
- GBV screening	X		X		X
HIV self-test (alternative to rapid)		X	X	X	
Rapid (blood-based) HIV test	X	X	X	X	X
STI symptom screen	X	X	X	X	X
Pregnancy testing (optional)	X	X	X	X	X
**DCP (including those persons on CAB-LA) and SOC**					
Measure height (baseline only) and weight	X		X		X
Assess self-reported PrEP/PEP use and HIV risk over prior 6 months	X		X		X
Complete Week 24 or 48 Visit eCRF			X		X
Hair sample collection if any PrEP or PEP doses taken in last 30 days			X		X
HIV RNA			X		X
**SOC Only**					
Study Visit	X		X		X
Referral to standard prevention options	X		X		X
Rapid (blood-based) HIV test	X		X		X
**Suspected seroconversion[3]**					

[1]Late visits for routine assessments will not be considered Protocol Violations, allowing flexibility during the COVID-19 pandemic
[2] This assessment will be done on all intervention participants at baseline
[3] See Figure 2
[4] Week 48 visits may occur between 40 and 56 weeks after Baseline

If CAB-LA is not available in country at the 48-week endpoint, all participants in the intervention arm will have the opportunity to conitinue on the study until week 96 or until CAB-LA is available in country, whichever occurs first, according to the schedules of evaluation described in Tables 5a and 6. Participants who are currently on CAB-LA at the Week 48 visit may continue on CAB-LA through Week 96. CAB-LA may not be initiated or restarted during the additional 48-week extension to Week 96. Prior to conducting procedures for visits after Week 48, participants in the intervention arm will be reconsented to extend their participation up to Week 96.

Table 5a. Schedule of Evaluations for Extension of Intervention Arm – Week 60 to Week 96

	Week 60	Week 72	Week 84	Week 96[3]
**DCP Intervention (For persons not receiving CAB-LA)[1]**				
Study visit	X	X	X	X
DCP intervention, including:	X	X	X	X
- Offer PrEP, PEP, prevention options[2,*]	X	X	X	X
- HIV testing options	X	X	X	X
- Visit location choice	X	X	X	X
- Structured assessment of barriers	X	X	X	X
- GBV screening		X		X

HIV self-test (alternative to rapid)	X	X	X	
Rapid (blood-based) HIV test	X	X	X	X
STI symptom screen	X	X	X	X
Pregnancy testing (optional)	X	X	X	X
Measure weight		X		X
Assess self-reported PrEP/PEP use and HIV risk over prior 6 months		X		X
Complete Week 72 or 96 Visit eCRF		X		X
HIV RNA		X		X
**Suspected seroconversion**[2]				

[1]Late visits for routine assessments will not be considered Protocol Violations, allowing flexibility during the COVID-19 pandemic or for other outbreaks.
[2] See Figure 2
[3] Week 96 visits may occur between 88 and 104 weeks after Baseline
*Participants will not be given the option to initiate or restart CAB-LA after the Week 48 visit; only other prevention options will be available.

Table 6. Schedule of Evaluations for CAB-LA users (weeks indexed by date of initial CAB LA injection) – Baseline to Week 96

	CAB Screening/ Baseline	CAB Week 4	CAB Week 8	CAB Week 16	CAB Week 24	CAB Week 32	CAB Week 40	CAB Week 48[7]	CAB Week 56	CAB Week 64	CAB Week 72	CAB Week 80	CAB Week 88	CAB Week 96[7]
**Measure weight**	X				X			X			X			X
**CAB LA injection**	X	X	X	X	X	X	X	X	X	X	X	X	X	
**STI Symptom Screen**	X	X	X	X	X	X	X	X	X	X	X	X	X	X
**Suspected Seroconversion**	X	X	X	X	X	X	X	X	X	X	X	X	X	X
**HIV Testing**														
-Rapid test (Country std. algorithm)	X	X	X	X	X	X	X	X	X	X	X	X	X	X
-HIV RNA[6]	X				X			X			X			X
-Stored plasma (2cc) for future HIV testing	X	X	X	X	X	X	X	X	X	X	X	X	X	X
**CAB PK**														
-Hair sample (if CAB-LA received in last 120 days)			X		X	X		X						
-Stored plasma (from 2 cc sample above)		X	X	X	X	X	X	X						
**Safety Labs**														
-Pregnancy testing[1]	X	X	X	X	X	X	X	X	X	X	X	X	X	X
-ALT Monitoring[4, 5]	X		X*		X			X*			X*			X*
**Adverse Event Monitoring**	X	X	X	X	X	X	X	X	X	X	X	X	X	X
**GBV Screening**	X				X			X			X			X
**Survey and IDI**														
-Participant CAB-LA Survey and IDI[2]	X				X			X						
-Provider and Clinic Staff DCP-LA Survey and IDI	X				X			X						
**Costing[3]**	X	X				X								
**For participants who stop CAB LA before end of extension study**														
-For 48 weeks from last injection: Recommend oral PrEP (TDF/3TC) to cover tail														
-Switch back to Q12 week visits - HIV RNA and pregnancy testing Q12 weeks														

[1]For women who become pregnant, repeat pregnancy tests are not needed. Women who become pregnant during the study will be followed to delivery for assessment of mother and infant status

[2] Participant survey/IDI will be conducted at start of CAB-LA and Weeks 24 and 48 from time of CAB-LA start or discontinuation.

[3]Costing will occur between weeks 24-48.

[4]ALT Monitoring will occur at baseline, Week 24 for all participants. *Per provider discretion, ALT monitoring will occur at Weeks 8, 48, 72 and 96.

[5]Bilirubin and additional labs indicated will be tested in the case of ALT increase.

[6]HIV RNA will be drawn at CAB-LA reinduction, as described in Table 6.

[7] Week 48 visits may occur between 40 and 56 weeks after Baseline; Week 96 visits may occur between 88 and 104 weeks after Baseline; only participants on CAB-LA at the Week 48 visit may take CAB-LA between weeks 48 and 96.

9. INFORMED CONSENT

9.1. Informed Consent Process

All participants in the DCP trials will be offered participation in the CAB-LA Extension. Consent forms will be translated from the original English to the language(s) spoken in the community. The consent form will be read to participants in their local language. Participants who agree to take part and sign the consent form will continue in the study. In this consent, women will be informed about the risks and benefits of CAB-LA during pregnancy and breastfeeding. In the case that participants become pregnant while using CAB-LA, they will be asked for an additional re-consent to follow-up for ascertainment of pregnancy and infant outcomes.

Participants will remain in the randomization arm they were assigned to at their initial DCP visit in Phase A. Participants in the SOC arm will consent to return for two additional follow-up visits at weeks 24 and 48 following re-consent for the CAB-LA extension. Intervention participants that do not choose CAB-LA will consent to continue the quarterly study visits and Intervention participants that choose CAB-LA will have study visits that align with their injection visits (every 4 weeks for the first two months and then every 8-weeks thereafter). Intervention participants will have flexibility to switch between PrEP, PEP, and CAB-LA. Unlimited switches between biomedical prevention options, including between CAB-LA and oral, will continue to be permitted during the extension, both to reflect participant choice and to bridge planned missed doses of CAB-LA. Clients and providers will discuss options for prevention and risk during regular visits and interim visits, in addition to continuous provider trainings on offering choice and refreshers for clients. Changes in prevention choices will be documented in study eCRF forms, which will include the CAB-LA option. DCP intervention participants that choose the CAB-LA option will be informed about the visit schedule, side effects, and risks and benefits, before consenting. If CAB-LA becomes available via the Ministry of Health during the trial, persons in the control arm will be eligible as per standard of care.

9.2. Assent Process

Participants aged 15-17 years old who meet the definition of 'mature or emancipated minors' as set by the Uganda National Council for Science and Technology and Kenya MoH can participate in these studies and will be consented on the adult participant consent forms. Participants aged 15-17 that do not meet the definition of 'mature or emancipated minors' may participate with parental co-signature. All participants will be asked to re-consent for the CAB-LA extension.

9.3. Documentation of Informed Consent

Documentation of written informed consent and verbal consent will be recorded on informed consent logs for all study activities. The logs will contain the participant's study ID, clinic ID, initials, name, language of consent used, and whether the individual signed the document or provided a fingerprint in the case of written consents, or verbally affirmed their consent in the case of verbal consents.

9.4. Stored Samples and Associated Data Considerations

Plasma samples will be collected and stored for retrospective HIV testing and PK.
Hair samples collected from participants on PrEP, CAB-LA or PEP in the extension will be collected and stored for later measurements of HIV-prevention drug levels, which will be conducted at the UCSF Hair Analytical Lab in the United States.
Blood samples from persons who acquire HIV in Phase A prevention trials will be stored for additional testing for HIV diagnostics (if indicated) and HIV drug resistance. Testing will be performed in-country when available (e.g. standard genotyping for HIV drug resistance). Specialized research assays for diagnostic and resistance testing (e.g. next-generation sequencing) will be performed at the University of Pittsburgh Parikh Lab in the United States.
Blood and urine samples will be stored for testing CAB-LA/PrEP/PEP drug levels performed at the UCSF Hair Analytical Lab in the United States.

Specimens may be held up to five years in order to complete HIV diagnostics, resistance, or PK tests as outlined in this protocol. The study may hold specimens to test in batches at later time since these tests are being performed solely for research, not for clinical care, or for a biobank for future use. Specimens will be destroyed after tests are performed.
All other biological samples collected during the study will be tested shortly after collection and any remaining specimen discarded.

10. SAFETY ASSESMENT OVERVIEW

10.1. Cabotegravir (GSK1265744A) Injectable Suspension- for intramuscular use: Product information

a. CAB-LA will be supplied by the ViiV Healthcare Limited Pharmaceutical Company

b. **Indications and Usage.** CAB-LA is indicated in at-risk adults and adolescents weighing at least 35 kg for pre-exposure prophylaxis (PrEP) to reduce the risk of sexually acquired HIV-1 infection. Individuals must have a negative HIV-1 test prior to initiating CAB-LA. CAB-LA will not be used as a supplement for PEP.

10.2. Administration Overview

CAB-LA will be offered as direct to inject only (no oral lead-in)
Injection: Single-dose vial containing 600 mg/3 mL (200 mg/mL) of cabotegravir is a white to light pink, free-flowing, extended-release injectable suspension
CAB-LA will be administered by trained healthcare staff by gluteal intramuscular injection only. Body Mass Index will be considered to ensure appropriate needle length is sufficient to reach the gluteal muscle.
Study participants will be tested for HIV infection prior to initiating CAB-LA, and with each subsequent injection of CAB-LA, including HIV RNA testing (Cepheid).
Prior to starting CAB-LA, healthcare staff will counsel study participants about the importance of adherence to scheduled dosing visits to help reduce the risk of acquiring HIV infection and development of resistance.

10.3. Pharmacy

a. The study sponsor, UCSF, will receive CAB-LA directly from ViiV Healthcare and distribute to the country pharmacies per country study drug shipping procedures

b. In-country pharmacists will receive and log the CAB-LA per country procedure and detailed in the CAB-LA Pharmacy SOPs

c. As described in 6c below, CAB-LA will stored at 2°C to 30°C (36°F to 86F) at clinic sites; proper storage and handling will be monitored by in-country pharmacy teams and validated by accountability logs and other source documents
d. Details on these procedures are outlined in the Standard Operating Procedures.
    i. CABPHARM-01 Study Drug Ordering and Acquisition
    ii. CABPHARM-02 Study Drug Accountability
    iii. CABPHARM-03 Study Drug Storage
    iv. CABPHARM-04 Dispensing and Administration of Study Drug
    v. CABPHARM-05 Disposition and Destruction of Study Drug
    vi. CABPHARM-06 Communication and Dissemination of Information to the Study Pharmacist

10.4. Injection Dosing

a. The initial injection dose of CAB-LA is a single 600-mg (3-mL) intramuscular injection of CAB-LA given 4-weeks apart for 2 consecutive times

b. After the 2 initiation injection doses given consecutively 4-weeks apart, the continuation injection dose of CAB-

LA will be a single 600-mg (3-mL) intramuscular injection of CAB-LA, every 8-weeks.

c. There is a +/- 7 day window for before or after the date the participant is scheduled to receive their next injection

10.5. Missed Doses

Study staff will provide adherence support to CAB-LA participants. When doses are missed, study staff will follow the applicable guidelines. Providers will work pro-actively and prospectively identify periods during which participants are likely to miss doses (eg travel). Participants will be provided with oral bridging prevention medication (TDF-FTC) to cover these periods. Upon return to clinic for planned or unplanned missed doses, CAB-LA dosing will be as per protocol below.

a. If a scheduled injection visit is missed or delayed by more than 7 days, the study clinician will determine when resumption is appropriate.

b. Recommended Dosing after a missed visit is detailed in **Table 7**.

Table 7. Missed Dosage Recommendations

Time since Last Injection	Recommendation
**If second injection is missed and time since first injection is:**	
Less than or equal to 8-weeks	Administer 600-mg (3-mL) gluteal intramuscular injection of CAB-LA as soon as possible, then continue to follow the every 8-weeks injection dosing schedule.
Greater than 8-weeks	Restart with 600-mg (3-mL) gluteal intramuscular injection of CAB-LA, followed by a second 600-mg (3-mL) initiation injection 4-weeks later. Then continue to follow the every 8-weeks injection dosing schedule thereafter.
**If third or subsequent injection is missed and time since prior injection is:**	
Less than or equal to 12 weeks	Administer 600-mg (3-mL) intramuscular injection of CAB-LA as soon as possible, then continue with the every 8-weeks injection dosing schedule.
Greater than 12-weeks	Restart with 600-mg (3-mL) gluteal intramuscular injection of CAB-LA, followed by the second 600-mg (3-mL) initiation injection dose 4-weeks later. Then continue with the every 8-weeks injection dosing schedule thereafter.

10.6. Contraindications. CAB-LA is contraindicated in individuals:

a. With unknown or positive HIV-1 status

d. With previous hypersensitivity reaction to cabotegravir

e. Receiving the following co-administered drugs for which significant decreases in cabotegravir plasma concentrations may occur due to uridine diphosphate glucuronosyltransferase:

i. Anticonvulsants: Carbamazepine, oxcarbazepine, phenobarbital, phenytoin

ii. Antimycobacterials: Rifampin, rifapentine

10.7. Drug Interactions

a. **Use of Other Antiretroviral Drugs after Discontinuation of CAB-LA.** Residual concentrations of cabotegravir may remain in the systemic circulation of individuals for prolonged periods (up to 12 months or longer). These residual concentrations are not expected to affect the exposures of antiretroviral drugs that are initiated after discontinuation of CAB-LA

b. **Potential for Other Drugs to Affect CAB-LA.** Cabotegravir is primarily metabolized by UGT1A1 with some contribution from UGT1A9. Drugs that are strong inducers of UGT1A1 or 1A9 are expected to decrease cabotegravir plasma concentrations; therefore, coadministration of CAB-LA with these drugs is contraindicated

c. **Established and Other Potentially Significant Drug Interactions.** Information regarding potential drug interactions with cabotegravir is provided in **Table 8** below.

Table 8. Drug Interactions with CAB-LA

Concomitant Drug Class: Drug Name	Effect on Concentration	Clinical Comment
**Anticonvulsants:**<br>Carbamazepine<br>Oxcarbazepine<br>Phenobarbital<br>Phenytoin	↓ Cabotegravir	Coadministration is contraindicated with CAB-LA due to potential forsignificant decreases in plasma concentration of CAB-LA.
**Antimycobacterials:** Rifampin<br>Rifapentine	↓ Cabotegravir	
**Antimycobacterial:**<br>Rifabutin	↓ Cabotegravir	When rifabutin is started before or concomitantly with the first initiation injection of CAB-LA, the recommended dosing of CAB-LA is one 600-mg<br>(3-mL) injection, followed 2 weeks later by a second 600-mg (3-mL) initiation injection and monthly thereafter while on rifabutin. When rifabutin is started at the time of the second initiation injection or later, the recommended dosing schedule of CAB-LA is 600 mg (3 mL) monthly while on rifabutin. After stopping rifabutin, the recommended dosing schedule of CAB-LA is 600 mg (3 mL) every 8 weeks.

d. **Drugs without Clinically Significant Interactions with Cabotegravir.** The following drugs can be co-administered with cabotegravir (non-antiretrovirals) or given after discontinuation of cabotegravir (antiretrovirals and non-antiretrovirals) without a dose adjustment: etravirine, midazolam, oral contraceptives containing levonorgestrel and ethinyl estradiol, and rilpivirine.

10.8. Drug Overdosage

There is no known specific treatment for overdose with CAB-LA. If overdose occurs, the participant will be treated appropriately, and further CAB-LA continuation determined by the Investigator.

11. HUMAN SUBJECTS PROTECTIONS

11.1. Vulnerable Participants

11.1.1. PREGNANT WOMEN AND FETUSES

a. CAB-LA will be used during pregnancy if the expected benefit justifies the potential risk to the fetus. Factors to consider include the long acting nature of CAB-LA, the actual gestational age, adherence, the risk of seroconversion and available data around the safety of study drugs and alternative PrEP regimens in pregnancy and breastfeeding.
b. In the HPTN 084 trial evaluating cabotegravir long-acting (LA) for pre-exposure prophylaxis (PrEP) in women in sub–Saharan Africa, no birth defects were reported of the 63 women that became pregnant while taking CAB-LA. Given the need for more data on pregnancy and breastfeeding while taking CAB-LA, participants will be monitored closely for side effects during pregnancy and breastfeeding during the study and up to 8 weeks post-partum.
c. Pregnant participants will sign a consent to be monitored for up to 8-weeks postpartum to ascertain the pregnancy and infant outcomes
d. Participants will be registered in the Antiretroviral Pregnancy Registry to contribute to the global scientific community's understanding of CAB-LA use in pregnancy and breastfeeding

e. It is recommended that participants of child bearing potential taking CAB LA should use an effective contraceptive method during the study, through any additional extension phase and for 52 weeks after the last injection if transitioning off CAB-LA, to avoid pregnancy. Participants that are currently on a contraceptive method at CAB-LA start will be informed of the risks and benefits of CAB-LA during pregnancy, if they chose to stop using contraceptives. Participants not on a contraceptive method when starting CAB-LA will be informed about the risks and benefits to CAB-LA during pregnancy if they become pregnant. CAB-LA will not be given to women who are pregnant at time of initial injection; women who become pregnant after starting CAB-LA will be allowed to remain on CAB-LA during pregnancy, as deemed appropriate by their provider, and will be re-consented for additional follow-up of pregnancy and infant outcomes.
f. It is not known if cabotegravir is present in human breast milk, affects human milk production, or has effects on the breastfed infant. Participants who are currently breastfeeding will be informed of the risks and benefits in the consenting process, and unknown effects on infants of exposure to CAB-LA through breastmilk.
g. No dosage adjustment of CAB-LA is necessary for participants with mild (creatinine clearance 60 to <90 mL/min) or moderate renal impairment (creatinine clearance 30 to <60 mL/min). In participants with severe renal impairment (creatinine clearance 15 to <30 mL/min) or end-stage renal disease (creatinine clearance<15 mL/min), increased monitoring for adverse effects will be provided
h. No dosage adjustment of CAB-LA is necessary for participants with mild or moderate hepatic impairment

11.1.2 CHILDREN

Youth aged 15 years and up are among the populations most in need of improved HIV outcomes and are a group targeted for participation this study. The Uganda National Council for Science and Technology (UNCST) has determined that individuals aged ≥14 years are able to provide independent consent for research if they have a sexually transmitted infection. In Uganda and Kenya, children of the age represented in this study can obtain HIV testing services and HIV care without parental consent if considered independent or an emancipated minor. Participants aged 15-17 taking part in HIV prevention procedures (CAB-LA Extension) and those taking part in the surveys who are not considered mature or independent minors will be required to sign an assent form, with separate parental consent. Information discussed in study visits and test results, including for HIV or STI testing, will not be disclosed to parent/guardians.
As with adults, participants <18 years will be given sufficient time to read or be read the consent form in their language and to ask any questions. None of the diagnostic tests or procedures provided in the study are more harmful to youth as compared to other groups.

11.1.3. ILLITERATE PARTICIPANTS

They study will enroll participants who may be economically or educationally disadvantaged. Participants in rural populations and those who are educationally disadvantaged are among those most at risk in their country for poor HIV care outcomes, and it is important to provide opportunities to participate in research to all people independent of their literacy. If a potential study participant is unable to read or write, his or her fingerprint will substitute for a signature, and a signature from a witness to the informed consent procedures will be obtained. Participants taking part in activities for which a verbal consent is required will have the consent text read to them in their language. As with other groups, illiterate participants will be provided sufficient time to be read the consent form or ask questions in order to understand study procedures.

12. RISKS

Privacy: The primary risk to study participation is breach of privacy. Care will be taken to protect the privacy of participants and parents/guardians. However, there is a risk that others may inadvertently see patients' medical information, and thus their privacy may be compromised.

Psychological Discomfort: There is the possibility of some psychological discomfort that may arise from questions and sensitive issues assessed in the stigma, sexual behavior, and youth life-skills and behaviors surveys and the in-depth qualitative interviews. We will train all study staff involved with surveys and qualitative interviews to recognize signs and symptoms of psychological discomfort and in the use of strategies to minimize such discomfort. This will include reminders that an individual may always choose to not answer any specific question that makes

them uncomfortable or to stop surveys and interviews at any time. Referral resources for significant issues will also be made available to study staff should subject referrals to care be desired.

Venipuncture and fingerprick sample collection: Study sampling during testing activities will be done by venipuncture or fingerprick, depending on the assay. The total volume of blood taken per participant, including blood taken for standard health facility evaluations, is too small to for participants to experience any serious side-effects, although a mild and transient feeling of discomfort or bruising may occur at the site of sampling and bruising.

Ensuring Necessary Medical or Professional Intervention in the Event of Adverse Effects: Procedures will be put in place for the referral and care, free of charge, for necessary medical or professional intervention in the event of the unlikely occurrence of severe adverse effects. Care will be provided at local Ministry of Health care clinics or nearby referral hospitals as necessary.

Potential Side Effects of CAB-LA
Most Common Side Effects:
- Pain, tenderness, hardened mass or lump, swelling, bruising, redness, itching, warmth, abscess, discoloration, or loss of sensation at the injection site
- stomach pain
- vomiting
- diarrhea
- muscle pain
- headache
- rash
- fever
- loss of appetite
- tiredness
- drowsiness
- sleep problems
- back pain
- nausea
- upper respiratory infection

Other side effects of cabotegravir may include:
a. Allergic reactions:
    - generally ill feeling
    - blisters
    - tiredness
    - muscle or joint aches
    - trouble breathing
    - fever
    - blisters or sores in the mouth
    - redness or swelling of eves
    - swelling of the mouth, face lips or tongues
    b. Liver Changes
c. Depression or Mood Changes
d. Hypersensitivity to Cabotegravir

Potential Risks associated with Pregnancy and Breastfeeding on CAB-LA:

There is very little information on the safety of CAB-LA during pregnancy and breastfeeding Participants will need to weigh the risks to their pregnancy and infant to the benefits of their HIV prevention based on the information provided below.
If participants become pregnant during the study and choose to take CAB-LA during pregnancy, staff will help participants understand that we have limited information on the safety of this drug during pregnancy. Of the 63 women who became pregnant while taking CAB-LA in Sub-Saharan African trials, there were no birth defects.

However, it is always possible there is some side effect we are unaware of as we learn more about CAB-LA in pregnancy. To ensure CAB-LA is as safe as possible for the mother and baby, we will monitor participants closely during pregnancy and post-partum. Participants will also be required to sign an informed consent that agrees to 8-weeks of monitoring after delivery as part of the safety assessment.
If participants are breastfeeding when they start this study or breastfeed after becoming pregnant on the study and choose to begin CAB-LA, staff will explain that we have limited information on the safety of this drug during breastfeeding. In studies of another drug, called dolutegravir, which is very similar to CAB LA no nursing babies had any problems. These babies were breastfed by mothers who had HIV and who were taking dolutegravir as treatment. Small amounts of dolutegravir were found in the breastmilk. It is possible some CAB-LA may pass to a baby through breastmilk.
CAB LA has been detected in the systemic circulation for up to 12 months or longer after an injection and the consequences of this level of exposure to the fetus are currently unknown. Consideration should be given to the potential for fetal exposure during pregnancy, even after the injections have been stopped.

- The safety of exposure of CAB- LA to infants through breastfeeding has not been established.
- Due to the nature of CAB-LA injections, CAB-LA may be present in breastmilk for up to, or beyond, a year after the final injection.
- The clinical consequences of exposure of the infant to CAB-LA are unknown.

While not required, to prevent any risk to the participant's pregnancy or to the infant, it is recommended that participants of child-bearing potential taking CAB-LA should use an effective contraceptive method during the study through any additional extension phase and for 52 weeks after the last injection if transitioning off CAB-LA, to avoid pregnancy.

13. SOCIAL IMPACT EVENTS

Individuals enrolled in this study may experience personal problems resulting from the study participation. Such problems are termed social impact events. Although study sites will make every effort to protect participant privacy and confidentiality, it is possible that participants' involvement in the study could become known to others, and that participants may experience stigmatization, discrimination or, in rare cases, intimate partner violence as a result of being perceived as being HIV-infected or at risk for HIV infection. For example, participants could be treated unfairly, could have problems being accepted by their families and/or communities, or could experience abuse by their partner. Problems may also occur in circumstances in which study participation is not disclosed, such as impact on employment related to time taken for study visits.

In the event that a participant reports a social impact event, every effort will be made by study staff to provide appropriate assistance, and/or referrals to appropriate resources. Social impact events that are judged by the investigators to be serious, unexpected, or more severe or frequent than anticipated, will be reported to the relevant IRBs.

14. BENEFITS

There will be no direct benefit to participants from participating in this study. The enhanced measures to improve oral PrEP, CAB-LA and PEP delivery, if assigned to the group that receives it, may help participants stay on medications, receive care, or reduce the likelihood of acquiring HIV infection, but this cannot be guaranteed. Participation in the study may benefit the community, scientists and doctors who work on providing HIV care in health centers such as in this study.

15. COMPENSATION

Participants will not receive compensation or payment in this study for taking part in research activities, except those participating in in-depth interviews.

16. PARTICIPANT PRIVACY AND CONFIDENTIALITY

Care will be taken to protect the privacy of participants and parents/guardians. Study participants will be identified only by their unique identification number on study documents except those maintained for contact information or consent documents. Participant study documents will be kept in individual files in secure filing cabinets in the study facilities. Testing results and sample and results transport documentation will be maintained in the local labs but will likewise not contain participant identifiers and will be accessed by study personnel only. After the study is over, all files containing personal identifiers such as participants' names, names of parents, guardians or relatives, and phone numbers and home locations will be destroyed.

In order to ensure data security and integrity, the following measures will be implemented:

- All members of the study team will be educated in the study protocol prior to the onset of the study.
- Detailed Standard Operating Procedures (SOPs) will be written for all project activities and be provided to relevant team members.
- Team members will be thoroughly trained on the SOP's.
- Where applicable, team members will receive additional training on the use of tablet computers.
- All data transcribed from paper will be double data entered or verified.
- All electronic data will be backed up regularly.
- All data will be transferred to the main Data Center in Kampala to the secure server. This sever is backed up on a daily basis and a monthly backup is stored off-site.
- All computers, including the tablets, will be password protected.
- All computers, including tablets, will be locked in a secure room each night.
- Log books and CRF's will be locked in a secure room each night.

17. STUDY DISCONTINUATION

The study may be discontinued at any time by the IRB, NIAID, or other government entities as part of their duties to ensure that research participants are protected.

18. COMMUNITY ADVISORY BOARD, STAKEHOLDERS, AND COMMUNICATION OF RESULTS

We will utilize a multilayered set of boards. These include: a) **stakeholder advisory board** consisting of representatives from the Uganda and Kenya Ministries of Health, IDRC and KEMRI research organizations, and PEPFAR implementing partners at the national and regional level, who will meet after Phase A is complete and prior to initiation of Phase B, and again at the conclusion of Phase B; b) formal **external scientific advisory board** meeting annually that includes experts in HIV prevention and treatment; experts in PrEP and youth in Uganda and Kenya; and experts in economics/development in East Africa; and c) **in-country advisory board** meeting annually with representation of MoH leads from HIV, non-communicable disease, the World Bank, PEPFAR implementing partners and HIV+ persons. Finally, **local community advisory boards** will provide invaluable input and communication with biannual meetings. We will have ongoing communication with the Kenya and Uganda National AIDS program, and with the PEPFAR implementing partners. Results of this study will be shared with the community, stakeholders, and at scientific meetings, with subsequent submission to publication.

19. ADMINISTRATIVE PROCEDURES

19.1. Regulatory Oversight

The proposed extension will be reviewed and approved by the IRBs of all the participating institutions in the U.S., Uganda and Kenya. This includes the UCSF Committee on Human Research (CHR), the Makerere University School of Medicine - Research and Ethics Committee (SOMREC), the Uganda National Council of Science and Technology (UNCST), the Kenya Medical Research Institute (KEMRI). As described in Section 18.0, this study will employ four levels of data and participant safety monitoring including Study Steering Committee oversight, US and host country Institutional Review Board (IRB) review, Data Safety Monitoring Board (DSMB) Review, and External Clinical Site Monitoring. **Table 9** describes event reporting guidelines to individual IRBs overseeing this study.

CAB-LA was very well tolerated in the registration studies. For this extension, SAEs will be captured in the intervention arm and Grade 3 and 4 AEs and non-serious ADRs leading to withdrawal will be captured for participants on CAB-LA. Grade 3 and 4 AEs will be graded and recorded using the NIAID Adverse Event Grading of Severity scale (https://rsc.niaid.nih.gov/clinical-research-sites/daids-adverse-event-grading-tables). Clinical Adverse Events not identified elsewhere are described in **Table 10.** The Clinical Management Team (CMT) will be notified of SAEs as soon as possible and will be reported per regulatory guidelines as stated in **Table 9** below.

Table 9. Reporting Guidelines

Institution	***Contact information***	***Type of Event***	***Reporting Timeframe***	***Reporting Method***
**UCSF IRB**	UCSF Committee for Human Research<br>3333 California Street, Suite 315<br>University of California<br>San Francisco, CA 94118<br>Campus Mailbox: 0962<br>Facsimile: (415) 502-134	SAEs/ Grade 3 or higher AEs that change the study risks or benefits, OR<br>necessitates modification to the IRB-approved consent document(s), and/or the IRB-approved application/protocol	Within 10-working days of awareness	Via online reporting system
**NIAID**	Melanie Bacon, Program Officer, NIH	SAEs or Harm determined to be possibly, probably or definitely related to study intervention<br><br>Participants administered CAB-LA:<br><br>All pregnancies	At the time of reporting to IRBs<br><br>One-week of awareness (Pregnancies)	Email
**ViiV**	GSK Medical Information, Safety Services & Vendor Management Group<br><br>oax37649@gsk.com	Participants administered CAB-LA:<br><br>All SAEs and Significant Safety Issues<br><br>All pregnancies<br><br>All Liver Events Grade 3 and higher	Within 72 hours of awareness (SAEs and Significant Safety Issues)<br><br>One-week of awareness (Pregnancies and Liver Events)	All SAEs and Significant Safety Issues: Email notification with SEARCH SAPPHIRE AE CRF, Country-specific SAE Reporting Forms, and attachment with supplemental information<br><br>Pregnancies: Email notification with Pregnancy Reporting CRF<br><br>Liver Events (Grade 3+): Email notification with Liver Reporting CRF
**KEMRI-SERU**	The Scientific and Ethics Review Unit<br>Through The Deputy Director CMR at<br>seru@kemri.org.	All reportable SAEs, SUSARs, and Harm	Notify/ report to SERU secretariat within 48 hours upon learning of the event and submit the full report within 5 working days of awareness	Via online reporting system to SERU secretariat
**PPB**	Pharmacy and Poisons Board	All reportable Grade 3 or higher SAEs, SUSARs, and Harm	Report to PPB within 7 working days of awareness	Via online reporting system to PPB ECCT (Expert Committee on Clinical Trials)
**MU-SOMREC**	Executive Secretary<br>Faculty of Medicine, Research and Ethics Committee<br>P.O. Box 7072<br>Kampala	All serious and unexpected events irrespective of relationship	Fatal and life threatening events within 3 working days of awareness and all	Via Regulatory Manager

			other SAEs within 7 calendar days	
**NDA**	National Drug Authority	All serious and unexpected events irrespective of relationship	Within 7 days of awareness	Via online reporting

19.2. Clinical Site Monitoring

Site monitors under contract to the University of California, San Francisco will visit participating clinical research sites to review participants records, including consent forms, CRFs, and laboratory records to ensure protection of study participants, compliance with IRB approved protocol/amendments, and accuracy and completeness of records. The monitors will inspect sites' regulatory files to ensure that local regulatory requirements, in addition to U.S. Federal regulations, are being followed.

19.3. Study Implementation

No study activities will commence prior to approval of US and host country IRBs and all other required local and international institutions. This study is funded and supported by the National Institutes of Health.

19.4. ClinicalTrials.gov

This protocol will be registered in ClinicalTrials.gov.

19.5. Adverse Event Procedures and Reporting Requirements

For persons receiving CAB-LA, Grade 3 and 4 AEs will be monitored in addition to SAEs.

Grade 3
Any grade 3 or higher clinical or laboratory AE observed prior to their first injection of active CAB will prompt consultation with the clinical officer (CO) prior to any injectable dosing.

At any time, participants who develop a Grade 3 AE or toxicity that is not specifically addressed in the Table below and is judged to be related to CAB-LA by the Investigator, CAB-LA use will be temporarily discontinued. In general, the investigator will re-evaluate the participant until resolution of the toxicity. For Grade 3 AEs deemed related to CAB-LA, will be permanently discontinued if improvement to severity ≤Grade 2 cannot be documented within 4 weeks of awareness. If CAB-LA is resumed after holding for a Grade 3 AE and the same Grade 3 AE recurs without alternative explanation, CAB-LA will be permanently discontinued. For Grade 3 AEs deemed unrelated to CAB-LA, the participant may continue with appropriate clinical management by the site, per local standards of care.

Grade 4

Any grade 4 or higher clinical or laboratory AE observed prior to first injection will prompt evaluation and may requiring holding of dosage, as determined by the CO and CMT. Participants who develop a Grade 4 AE or toxicity that is not specifically addressed in **Table 11** (regardless of relationship to CAB-LA) will have the CAB-LA injections temporarily discontinued. CAB-LA use will not be resumed if the Grade 4 AE is considered by the Investigator to be related to the injections. If CAB-LA use is resumed and the same Grade 4 AE recurs at Grade 4 level at any time, CAB-LA will be permanently discontinued, unless an alternative explanation for the recurrence is clearly documented.

Pregnancy

Any pregnancy that occurs during in participants taking CAB-LA must be reported using the Pregnancy Reporting Case Report form to NIAID and ViiV Healthcare within **one week** of learning of its occurrence. Pregnancies that are

associated with SAEs will be reported within 72 hours using the Adverse Event CRF, Country-specific SAE Reporting form, and supplemental information attached.
The pregnancy must be followed up to determine outcome (including premature termination) and status of mother and child(ren), which must also be reported to NIAID and ViiV Healthcare using the Pregnancy Outcomes case report form. The pregnancy outcome should also be reported to ViiV Healthcare in accordance with the terms of the contract.
Pregnancy complications and elective terminations for medical reasons must be reported as an adverse event or serious adverse event. Spontaneous abortions must be reported as a serious adverse event in accordance with SAE reporting timelines.
Any serious adverse event occurring in association with a pregnancy brought to the investigator's attention after the participant has completed the study and considered by the investigator as possibly related to the investigational product, must be promptly reported to NIAID and ViiV Healthcare.

Table 10. DAIDS Table for Grading the Severity of Adult and Pediatric Adverse Events

PARAMETER	GRADE 1 MILD	GRADE 2 MODERATE	GRADE 3 SEVERE	GRADE 4 POTENTIALLY LIFE-THREATENING
**Clinical** adverse event **NOT** identified elsewhere in the grading table	Mild symptoms causing no or minimal interference with usual social & functional activities with intervention not indicated	Moderate symptoms causing greater than minimal interference with usual social & functional activities with intervention indicated	Severe symptoms causing inability to perform usual social & functional activities with intervention or hospitalization indicated	Potentially life-threatening symptoms causing inability to perform basic self-care functions with intervention indicated to prevent permanent impairment, persistent disability, or death

20. TOXICITY MANAGEMENT

20.1 Participant Discontinuation

General Criteria for Discontinuation of CAB-LA
Participants may voluntarily discontinue CAB-LA for any reason at any time. Investigators will permanently discontinue participants from CAB-LA per protocol for any of the specific criteria below. Investigators also may permanently discontinue participants for reasons not shown here (e.g., to protect participants' safety and/or if participants are unable or unwilling to comply with CAB-LA use procedures).
The criteria for permanent discontinuation of CAB-LA use for an individual participant are:

- CAB-LA -related toxicity requiring permanent discontinuation per the guidelines above and below
- Request by participant to permanently terminate use of CAB-LA
- Clinical reasons determined by the Investigator
- Acquires HIV infection

CAB-LA will be temporarily withheld from a participant for any of the following reasons:

- Report of use of prohibited concomitant medications. CAB-LA injections may resume when the participant reports that he/she is no longer taking the prohibited medication, provided other reasons for temporary hold/permanent discontinuation do not apply.

- The participant is unable or unwilling to comply with required study procedures such as HIV testing and routine laboratory assessments, or otherwise might be put at undue risk to their safety and well-being by continuing CAB-LA use, according to the judgment of the Investigator.
- The participant has one or more reactive HIV test results or expresses a concern about having acute HIV infection.

20.2. Guidance on Toxicity Management for Specified Toxicities:

Table 11: Nausea, Vomiting, and Diarrhea

CONDITION AND SEVERITY	IMMEDIATE ACTION	FOLLOW-UP AND MANAGEMENT
	**Nausea, Vomiting, and Diarrhea**	
Grade 1 and 2	Continue CAB-LA	Will be treated symptomatically with hydration, oral antiemetic therapies or antiemetic suppositories at the discretion of the Investigator. The Investigator will order any clinically relevant laboratory analyses (per judgment of the Investigator).
Grade ≥ 3	Discontinue CAB-LA temporarily	Participants with Grade 3 nausea, vomiting, or diarrhea, for which an alternative etiology is not established, will discontinue CAB-LA temporarily until Grade 2 or lower and be treated symptomatically. Should condition(s) not improve to Grade ≤ 2 within 7 days, the Investigator will consider temporary discontinuation or progressing to permanent discontinuation of CAB-LA.

Liver chemistry monitoring
Liver chemistry monitoring is at the discretion of the provider and will occur at baseline and Week 24. Additional ALT measurements will be taken and Weeks 8 and 48 per provider discretion. Cabotegravir will be stopped if certain protocol specified liver event stopping criteria are met, or hepatotoxicity is suspected.

Liver Event Stopping Criteria+
CAB must be stopped if any of the following Liver Event Stopping Criteria are met, or if the Investigator believes that it is in the best interests of the participant:

- ALT >= 3xULN AND bilirubin >= 2xULN
- ALT >=5xULN for more than 2 weeks
- ALT >=8xULN
- ALT >=3xULN (or >=3x baseline ALT) with symptoms of worsening of acute hepatitis or hypersensitivity such as fatigue, nausea, vomiting, right upper quadrant pain or tenderness, fever, rash or eosinophilia

Consideration should also be given to stopping CAB in any participant with aminotransferase elevations > 3x ULN who cannot be monitored every 1-2 weeks.

When a Liver Event Stopping Criterion is met, the treating physician must do the following:

- **Immediately stop CAB-LA**
- If ALT >= 3xULN **and** bilirubin >=2xULN, report as a serious adverse event (SAE) within **72 hours** using the SAE case report form.
- For **all** events meeting Liver Event Stopping Criteria, perform repeat liver chemistries and liver event follow up assessments (ideally within 24 hours of knowledge of the liver chemistry elevations) as described in the Follow-Up Assessment below and report to UCSF, NIAID, and ViiV Healthcare using the Liver Event Case Report form within one week of first becoming aware of the event.
- A specialist or hepatology consultation is recommended if available.
- Monitor participants 2 times a week until liver chemistries resolve, stabilise or return to within baseline values.

Follow up Assessment following ANY Liver Stopping Event
Make every attempt to carry out liver event follow-up assessments as described below for the laboratory tests that are available locally.

- Viral hepatitis serology, including:
- Hepatitis A immunoglobulin M (IgM) antibody;
- HBsAg and hepatitis B core antibody;
- Hepatitis C RNA;
- Hepatitis E IgM antibody.
- Cytomegalovirus IgM antibody.
- Epstein-Barr viral capsid antigen IgM antibody (or if unavailable, obtain heterophile antibody or monospot testing).
- Syphilis screening.
- Drugs of abuse screen, including alcohol. Record alcohol use on the liver event case report form if using.
- Serum acetaminophen/paracetamol levels
- Serum creatinine phosphokinase (CPK) and lactate dehydrogenase (LDH).
- Fractionate bilirubin, if total bilirubin >=1.5xULN.
- Obtain complete blood count with differential to assess eosinophilia.
- Anti-nuclear antibody, anti-smooth muscle antibody, Type 1 anti-liver kidney microsomal antibodies, and quantitative total immunoglobulin G (or gamma globulins).
- Gamma glutamyl transferase (GGT), glutamate dehydrogenase (GLDH), and serum albumin
- International normalised ratio (INR)
- Liver imaging (ultrasound, magnetic resonance, or computerised tomography) and /or liver biopsy to evaluate liver disease. [A Liver Imaging and/or Liver Biopsy Case Report Form is available if required].
- Obtain history and record the appearance or worsening of clinical symptoms of liver injury, or hypersensitivity, fatigue, decreased appetite, nausea, vomiting, abdominal pain, jaundice, fever rash as relevant on the AE/SAE report form.
- Obtain history and record use of concomitant medications including acetaminophen, herbal remedies, other over the counter medications or putative hepatotoxins.

Liver-Safety- CAB-LA Restart
"Drug restart" refers to resuming study treatment in which there is a clear cause other than Drug Induced Liver Injury (DILI). For long acting agents, a restart may actually be a continuation of therapy rather than a true restart due to the timeframe between dose administration relative to decision making on allowing a restart.

Participants who meet Liver Event Stopping Criteria should NOT restart CAB-LA unless there is a compelling alternative cause for the liver chemistry abnormalities.

"Drug restart" with CAB-LA in a participant who has a Liver Stopping Event may be permitted when liver chemistries improve to within 1.5x baseline and ALT < 3 xULN, provided the criteria set out in the Liver Safety Restart Guidelines are met. This should take into account when the next scheduled injection of CAB LA is due. If the restart criteria are not met by the time window for the next injection, consideration should be made to transitioning participants onto an alternative regimen.

If restart is **not allowed or not granted** by the Study's Governing Body or Principal Investigator, permanently discontinue study treatment. Participants may continue participation in the study for any protocol specified follow up assessments.

Liver Safety Restart Guidelines
If a causal relationship between the liver event and CAB-LA cannot be ruled out, then CAB-LA must be permanently discontinued and the participant not restarted.

Note: Any decision regarding potential restart should take into account when the next scheduled injection of CAB LA is due. If the restart criteria below are not met by the time window for the next injection(s), consideration should be made to transitioning participants onto an alternative regimen.

CAB-LA Restart Following Transient Resolving Liver Events Not Related to CAB-LA
Restart can be considered when liver chemistries improve to within 1.5x baseline and ALT<3xULN where:

- Liver chemistries have a clear underlying cause other than drug-induced liver injury (e.g. biliary obstruction, pancreatic events, hypotension, acute viral hepatitis). Furthermore, there should be no evidence of alcoholic hepatitis or hypersensitivity, and the drug is not associated with HLA markers of liver injury.
- The participant is receiving compelling benefit and the benefit of drug restart exceeds risk. The long acting nature of CAB LA should be taken into consideration.
- Approval from the Principal Investigator and Ethics Committee or Institutional Review Board for the drug restart has been obtained.
- The participant has been provided with a clear description of the possible benefits and risks of drug restart, including the possibility of recurrent, more severe liver injury or death and the long acting nature of CAB injections.
- The participant has also provided signed informed consent specifically for the restart. Documentation of informed consent must be recorded in the study file.
- Following drug restart, the participant will return to the clinic once a week for liver chemistry tests for one month or for as long as clinically indicated and then laboratory monitoring may resume as per protocol. If protocol defined stopping criteria for liver chemistry elevations are met, study drug must be permanently stopped.

Hypersensitivity
Hypersensitivity reactions have been reported in association with other integrase inhibitors and were characterized by rash, constitutional findings and sometimes organ dysfunction, including liver injury. Administration of CAB oral lead-in was used in clinical studies to help identify patients who may be at risk of a hypersensitivity reaction. While there have been no confirmed occurrence of a severe hypersensitivity reactions attributable to cabotegravir in clinical trials to date, investigators should remain vigilant regarding their occurrence and should discontinue CAB-LA and other suspect agents immediately if signs or symptoms of hypersensitivity reactions develop (including, but not limited to, severe rash, or rash accompanied by fever, general malaise, fatigue, muscle or joint aches, blisters, oral lesions, conjunctivitis, facial oedema, hepatitis, eosinophilia or angioedema. Clinical status, including laboratory parameters with liver transaminases, should be monitored and appropriate therapy initiated.

All rash and suspected hypersensitivity events should be assessed with special attention to systemic symptoms, laboratory abnormalities, or mucosal involvement. Close clinical follow-up, including follow-up of laboratory abnormalities, and appropriate medical intervention, including referral to dermatologist as appropriate, should be instituted for these events. The prolonged release characteristics of CAB LA should be taken into consideration when these products are discontinued.

Participants should be instructed to contact the Investigator as soon as possible if they develop a rash or allergic symptoms on study.

Suicidal Ideation
There have been reports of depression and suicidal ideation and behaviours (particularly in patients with a pre-existing history of depression or psychiatric illness) in some people living with HIV being treated with integrase inhibitors. Depression has also been reported in individuals receiving cabotegravir for PrEP.

Participants should be monitored appropriately for any unusual changes in behaviour, before and during the course of the study. Participants with depressive symptoms or suicidal ideation and behaviours on CAB-LA should be promptly evaluated. It is recommended that the investigator consider mental health consultation or referral for participants who experience signs of suicidal ideation or behaviour.

Confirmation of suspected HIV infection
In cases of suspected HIV-infection, clinic staff are trained and use a seroconversion SOP for reference to proceed as depicted in Figure 2. For all cases, the clinical management team is notified immediately, as simultaneous confirmatory testing, additional sample collection (including stored samples for further HIV testing and PK), counseling, and linkage to ART care are initiated.)

Figure 2. HIV Seroconversion Algorithm and Sample Collection

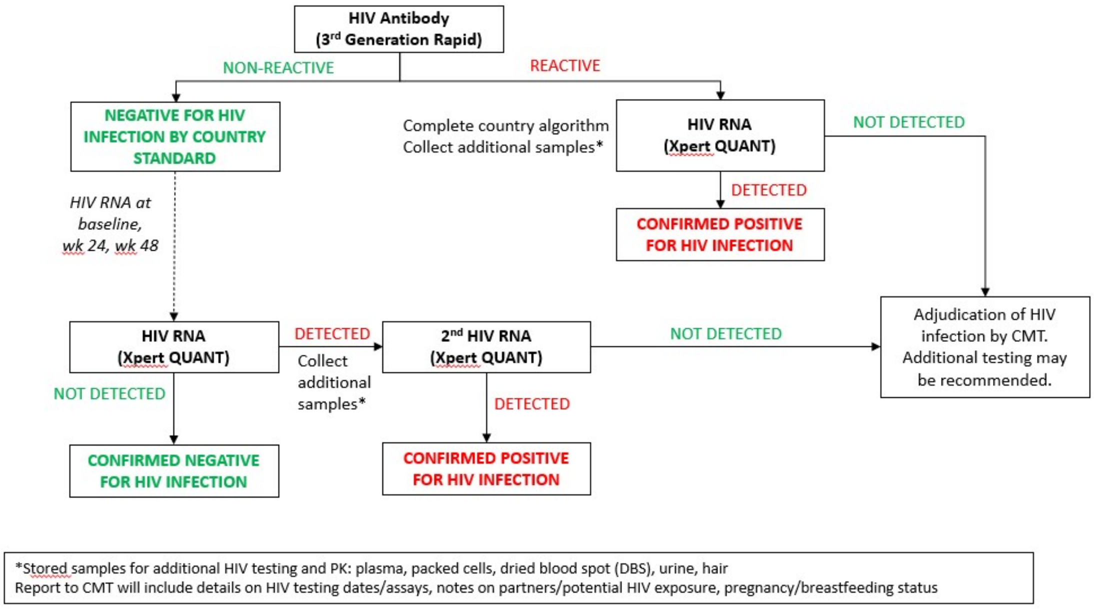

For persons receiving CAB-LA, the following approach will be used for guidance on testing and administration of CAB-LA injections (Figure 3).

Figure 3. Algorithm for participants receiving CAB-LA Injections

Figure 3: Real-time HIV testing for CAB LA Extension:
Rapid HIV test per country standard algorithm (all visits) + HIV RNA at baseline, week 24, week 48

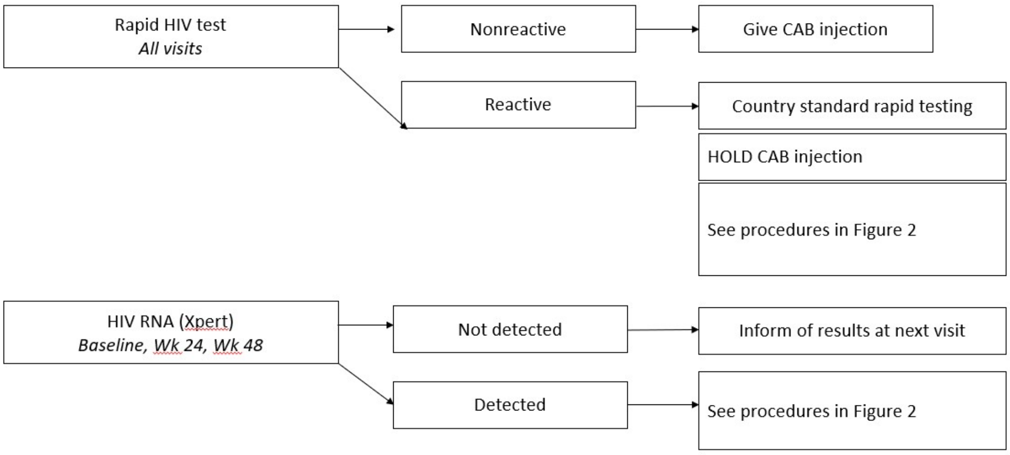

21. ANALYSES

21.1. Overview

This section provides an overview of the statistical consideration.
The primary study endpoint in this extension will be biomedical covered time defined as the proportion of follow-up time that the participant is protected from HIV infection by any PEP or PrEP (inclusive of CAB-LA). Follow-up will be over the 48 weeks following re-consent for the CAB-LA extension. At risk covered time will be a secondary outcome, defined as the proportion of at-risk follow-up time that the participant is protected from HIV infection by any PEP or PrEP (inclusive of CAB-LA). We will also conduct sensitivity analyses, varying the definitions of coverage and risk. For example, we will also examine covered time from CAB-LA only (i.e., exclusive of oral PrEP or PEP), and we will conduct a sensitivity analysis in which we treat time after loss to follow-up among persons not known to have moved out of the study region and time after study withdrawal as presumed to be non-covered.
For our primary objective in this extension, we will compare covered time during the 48 week CAB-LA extension between the intervention and control arms. For improved efficiency, Targeted Maximum Likelihood Estimation (TMLE) will be used to adjust for differences in baseline participant characteristics between the two trial arms, accounting for the stratified randomization design. In secondary analyses, we will further use TMLE to i) adjust for measured differences between arms at time of start of the CAB-LA extension period; and, ii) adjust for baseline and time-varying factors that are predictive of the outcome and that differ between persons that do vs. do not have their follow-up time censored. The primary analysis will pool over the trials, accounting for clustering inherent in the community trial; secondary analyses will stratify by trial site. We will test the null hypothesis that covered time is the same between arms with a two-sided test at the 5% significance level.
Our secondary objective to evaluate implementation will follow the RE-AIM framework and corresponding measures (defined in Table 4). Analyses assessing the primary measures of Reach, Adoption, Implementation and Maintenance will be based on standard descriptive statistics. Analyses to evaluate Effectiveness will compare covered time over the 48 week CAB-LA extension period to covered time prior to the CAB-LA extension among intervention participants. We will adjust for measured changes in participant characteristics between these two follow-up periods, using a TMLE designed for serial measures. Measures of control participants will also be used to adjust for temporal trends. We will test the null hypothesis that coverage of the DCP model with CAB-LA is the same as coverage of the DCP model without CAB-LA. We will again use a two-sided test at the 5% significance level. Primary analyses will again pool over trials, while secondary analyses will be stratified by trial. In addition, we will evaluate marginal and adjusted predictors of CAB-LA uptake and persistence. Our tertiary objective to evaluate changes in knowledge, awareness, and acceptability/satisfaction will be based on applying TMLE to survey data, accounting for repeated measures.

Additional descriptive analyses will include HIV incidence and safety. HIV seroconversions will be measured in each arm and incidence rates will be calculated using person time at risk. Safety Grade 3 and Higher AEs and SAEs will be measured for participants using CAB-LA and incidence rates will be calculated during person time exposed. These analyses will be complemented with costing evaluations as well as participant and provider qualitative data from IDI.
All analyses of the community-based trial will account for clustering.

21.2. Pharmacokinetic Analyses

We will perform pharmacokinetic (PK) analyses of cabotegravir concentrations in plasma and small hair samples with two objectives. 1) To examine hair levels of CAB as predictors of effectiveness; 2) To examine factors associated with CAB PK variability and study correlations between CAB hair and plasma levels at the end of dosing intervals and during the PK tail. In the phase 3 trials of CAB LA, plasma concentrations of CAB did not fully explain CAB breakthrough infections (especially since failures occurred despite on-time injections) and therefore PK metrics, such as hair, that reflect longer-term exposure, may provide important insights regarding CAB exposure in addition to plasma. Drug concentrations of cabotegravir will be analyzed via liquid chromatography tandem mass spectrometry (LS-MS/MS) at the Hair Analytical Laboratory at UCSF. Plasma samples will be collected prior to each injection, while hair samples will be collected at weeks 8, 24, 32, and 48 after CAB LA start.
First, we will analyze hair concentrations of cabotegravir in plasma and small hair samples as predictors of effectiveness. We will take the proximal 2 cm of hair from the scalp and segment the hair into two 1.0 cm segments (reflecting the prior two months of drug exposure in 1 month intervals), analyzing CAB levels in each segment. We will analyze hair levels from each collection visit from all participants who acquire HIV infection after starting CAB LA and three randomly selected controls who did not acquire HIV to compare exposure metrics from those for with and without HIV acquisition. We use sampling fraction weights to properly combine data from participants who acquire HIV (sampled with certainty) and controls in order to obtain accurate estimates for the entire study population.
Second, to examine factors associated with PK variability, we will perform univariate linear mixed effects regression using hair levels of CAB assessed as continuous outcomes with candidate predictor variables that may influence exposure, such as age, sex and body mass index (BMI), as well as plasma levels, and delays in injection visits. We will then build multivariate models to assess how associations with hair levels change when controlled for other factors. We will examine scatterplots and Spearman correlation coefficients between hair and plasma concentrations of CAB, including at the end of the dosing interval (using the hair concentration in the proximal 0.5 cm of hair since hair is collected before injections) and during the PK tail for participants who elect to stop CAB LA or who acquire HIV and start an oral ART regimen.

21.3. Analyses of In-Depth Interviews

Qualitative in-depth interviews will be conducted among purposively-selected samples of study participants and providers, to ascertain the perceptions of and experiences with CAB-LA use and delivery. Data will analyzed using a framework analysis approach, wherein transcribed and translated data are inductively coded by a cross-regional team of trained qualitative researchers, the initial sets of findings are consolidated and grouped into conceptual categories, and a framework matrix is used to further condense findings tied most salient to the research questions.

21.4. Costing

Costs will be estimated as the sum of the product of resources (e.g. staff minutes) times unit costs (e.g. compensation levels). “Economic” costs (the true value of resources consumed or “opportunity costs”) will be assessed by identifying the value of subsidized or donated resources with information from databases (e.g. wage rates) and donors. We will use prices prevailing at the time of study onset and will convert any locally incurred costs using the Uganda/Kenya Shilling / US dollar exchange rate at that time. Costs of CAB-LA implementation will be compared to the costs of oral PrEP implementation estimated during the primary trial to estimate the incremental cost of CAB-LA implementation.

21.5. Power and Sample size considerations

For each ongoing trial, we assume 90% of participants consent to the extension (reflecting the high retention in the ongoing trials seen to date). Of those, we assume 90% are retained through the extension. In the individually randomized ANC and OPD trials, we anticipate >80% power to detect a 11% or greater absolute increase in prevention coverage between arms with 162 participants/arm (assuming σ =0.35). In the cluster-randomized community-based trial, we anticipate >80% power to detect a 15% or greater absolute increase in prevention coverage between arms with ~20 participants in each of the 16 clusters (assuming mean prevention coverage in the standard of care arm of 10%, σ =0.35, and a matched-pair coefficient of variation km=0.25). These calculations are expected to be conservative given the ongoing enrollment and primary approach to conduct a pooled analysis as well as the increased precision from stratified randomization and through covariate adjustment with TMLE. Minimum detectable effect sizes varying these assumptions (including allowing for lower retention in either the ongoing or extension studies) are shown in Figure 4.

Figure 4. Minimum detectable effect sizes for the primary hypothesis

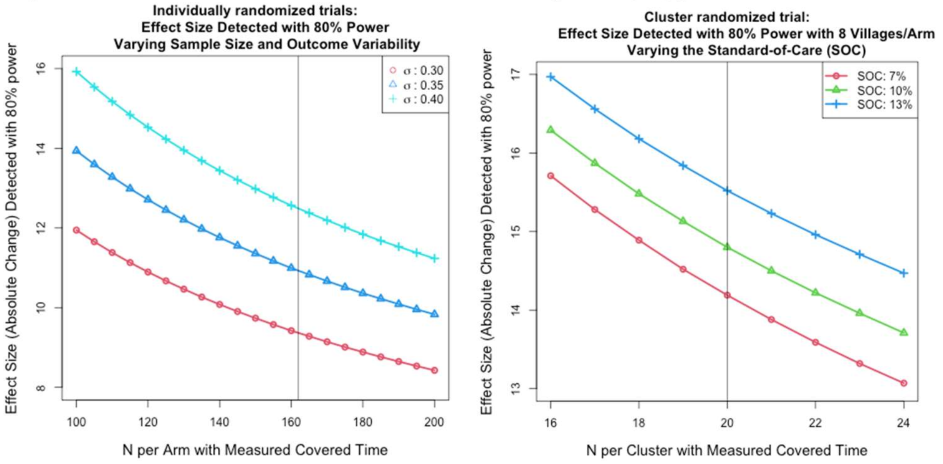

22. DATA HANDLING AND RECORD KEEPING

This is an implementation study; the primary risks for a client would be any loss of confidentiality, or risk from blood draw in measurement of the HIV outcome. For risk mitigation, all participants undergo informed consent in their native language, staff are fully trained on confidentiality. The SEARCH Sapphire team has SOPs and procedures for data collection and security. Because CAB-LA is not yet approved in Uganda or Kenya, we will be recording Grade 3 and 4 AEs and reporting to these as required to regulatory authorities.

22.1. Data Management Responsibilities

Data management for this study will be overseen by the Infectious Diseases Research Collaboration (IDRC) Data Management Center located in the main IDRC research complex in Nakasero, Kampala, Uganda. The Data Management Center (DMC) is also responsible for providing IT support to all staff members.

<u>Data Management System</u>
For any study components using paper data collection, IDRC has designed and developed a custom Data Management System that is used to manage the data, using Microsoft SQL Server as the backend for all data storage. MS Access is used for double data entry and SAS programs are used for comparing 1st and 2nd entry and generating discrepancy reports. In addition, the DMC has developed custom Visual Basic programming for direct survey and data entry of data in the field. Data Transformation Services/ SQL Server Integration Services (DTS/SSIS) packages are used to automatically import/export any new data and stored procedures written in T-SQL are used to automatically generate new data queries on a daily basis. There is a web interface to the whole system written in ASP.NET to allow users to view the data, view reports, and even modify the data they are authorized to access. With regular backups and a full audit trail, the whole system is regulatory compliant (21 CFR Part 11).

22.2. Essential/Source Documents and Access to Source Data/Documents

Data is collected as a mixture of paper Case Report Forms (CRFs) and logs and electronic data capture from health facility databases and field data tablets. Data is entered locally and uploaded to the DMC after quality assurance measures have taken place. Only study investigators and staff affiliated with the study will have access to source data.

External data sharing will be made through a data request proposal procedure coordinated by the PIs and the IDRC Data Department. A standardized data request sheet will be completed by outside investigators and reviewed by the senior management and/or the Principal Investigator. Data and associated documentation will be available to users under a data-sharing agreement that provides for: (1) a commitment to using the data only for research purposes and not to identify any individual participant; (2) a commitment to securing the data using appropriate computer technology; and (3) a commitment to destroying or returning the data after analyses are completed. User registration is required to access or download files. As part of the registration process, users must agree to the conditions of use governing access to the public release data, including restrictions against attempting to identify study participants, destruction of the data after analyses are completed, reporting responsibilities, restrictions on redistribution of the data to third parties, and proper acknowledgement of the data resource.

22.3. Quality Control and Quality Assurance

The data management system is designed to collect, transfer and store data for Data Management Center studies. Data from any paper records will be 100% double entered into the system via Microsoft Access Databases or Web-based data entry screens. Locally collected data is compressed, password-protected, and then securely uploaded to a cloud based server (Network Solutions, Inc.) using secure-FTP via the FileZilla application. Cloud-based uploads are downloaded daily to the Kampala DMC. After the data has been input into the Data Management Center server, it is electronically verified and is then written to the SQL database. Edit checks or queries are run nightly and the results are posted to a secure clinical trials website so that the sites can electronically address problems with the data the next day. The site corrects data via the website and the database is updated automatically.

In order to ensure data security and integrity, the following measures will be implemented:

- All members of the study team will be educated in the study protocol prior to the onset of the study.
- Detailed Standard Operating Procedures (SOPs) will be written for all project activities and be provided to relevant team members.
- Team members will be thoroughly trained on the SOP's.
- Where applicable, team members will receive additional training on the use of tablet computers.
- All data transcribed from paper will be double data entered or verified.
- All electronic data will be backed up regularly.
- All data will be transferred to the main Data Center in Kampala to the secure server. This sever is backed up on a daily basis and a monthly backup is stored off-site.
- All computers, including the tablets, will be password protected.
- All computers, including tablets, will be locked in a secure room each night.
- Any Log Books and CRF's will be locked in a secure room each night.

23. PUBLICATION POLICY

The findings from this study may be published in a medical journal. No individual identities will be used in any reports or publications resulting from the study. The researchers will publish results of the study in accordance with NIH, UCSF, UNCST, KEMRI, and Makerere University guidelines.

24. REFERENCES

1. Global AIDS Update 2019: Communities at the centre. UNAIDS, 2019 2019/07/16/. Report No.
2. Havlir DV, Balzer LB, Charlebois ED, Clark TD, Kwarisiima D, Ayieko J, Kabami J, Sang N, Liegler T, Chamie G, Camlin CS, Jain V, Kadede K, Atukunda M, Ruel T, Shade SB, Ssemmondo E, Byonanebye DM, Mwangwa F, Owaraganise A, Olilo W, Black D, Snyman K, Burger R, Getahun M, Achando J, Awuonda B, Nakato H, Kironde J, Okiror S, Thirumurthy H, Koss C, Brown L, Marquez C, Schwab J, Lavoy G, Plenty A, Mugoma Wafula E, Omanya P, Chen YH, Rooney JF, Bacon M, van der Laan M, Cohen CR, Bukusi E,

Kamya MR, Petersen M. HIV Testing and Treatment with the Use of a Community Health Approach in Rural Africa. N Engl J Med. 2019;381(3):219-229. PubMed PMID: 31314966.

3. Iwuji CC, Orne-Gliemann J, Larmarange J, Balestre E, Thiebaut R, Tanser F, Okesola N, Makowa T, Dreyer J, Herbst K, McGrath N, Barnighausen T, Boyer S, De Oliveira T, Rekacewicz C, Bazin B, Newell ML, Pillay D, Dabis F, Group ATS. Universal test and treat and the HIV epidemic in rural South Africa: a phase 4, open-label, community cluster randomised trial. The lancet HIV. 2018;5(3):e116-e125. PubMed PMID: 29199100.

4. Hayes RJ, Donnell D, Floyd S, Mandla N, Bwalya J, Sabapathy K, Yang B, Phiri M, Schaap A, Eshleman SH, Piwowar-Manning E, Kosloff B, James A, Skalland T, Wilson E, Emel L, Macleod D, Dunbar R, Simwinga M, Makola N, Bond V, Hoddinott G, Moore A, Griffith S, Deshmane Sista N, Vermund SH, El-Sadr W, Burns DN, Hargreaves JR, Hauck K, Fraser C, Shanaube K, Bock P, Beyers N, Ayles H, Fidler S, Team HS. Effect of Universal Testing and Treatment on HIV Incidence - HPTN 071 (PopART). N Engl J Med. 2019;381(3):207-218. PubMed PMID: 31314965; PubMed Central PMCID: PMCPMC6587177.

5. Makhema J, Wirth KE, Pretorius Holme M, Gaolathe T, Mmalane M, Kadima E, Chakalisa U, Bennett K, Leidner J, Manyake K, Mbikiwa AM, Simon SV, Letlhogile R, Mukokomani K, van Widenfelt E, Moyo S, Lebelonyane R, Alwano MG, Powis KM, Dryden-Peterson SL, Kgathi C, Novitsky V, Moore J, Bachanas P, Abrams W, Block L, El-Halabi S, Marukutira T, Mills LA, Sexton C, Raizes E, Gaseitsiwe S, Bussmann H, Okui L, John O, Shapiro RL, Pals S, Michael H, Roland M, DeGruttola V, Lei Q, Wang R, Tchetgen Tchetgen E, Essex M, Lockman S. Universal Testing, Expanded Treatment, and Incidence of HIV Infection in Botswana. N Engl J Med. 2019;381(3):230-242. PubMed PMID: 31314967.

6. 90-90-90. An ambitious treatment target to help end the AIDS epidemic. UNAIDS. 2014.

7. UGANDA POPULATION-BASED HIV IMPACT ASSESSMENT. UPHIA 2016–2017. Summary Sheet: Preliminary Findings. August 2017. Available at: https://www.afro.who.int/sites/default/files/2017-08/UPHIA%20Uganda%20factsheet.pdf. 2017.

8. Sovershaeva E, Shamu T, Wilsgaard T, Bandason T, Flaegstad T, Katzenstein D, Ferrand RA, Odland J. Patterns of detectable viraemia among children and adults with HIV infection taking antiretroviral therapy in Zimbabwe. International journal of infectious diseases : IJID : official publication of the International Society for Infectious Diseases. 2019;78:65-71. PubMed PMID: 30391420.

9. ZIMBABWE POPULATION-BASED HIV IMPACT ASSESSMENT. ZIMPHIA 2015–2016. Summary Sheet: Preliminary Findings. December 2016. Accessed at: https://phia.icap.columbia.edu/wp-content/uploads/2016/11/ZIMBABWE-Factsheet.FIN_.pdf.

10. Petersen M, Balzer L, Kwarsiima D, Sang N, Chamie G, Ayieko J, Kabami J, Owaraganise A, Liegler T, Mwangwa F, Kadede K, Jain V, Plenty A, Brown L, Lavoy G, Schwab J, Black D, van der Laan M, Bukusi EA, Cohen CR, Clark TD, Charlebois E, Kamya M, Havlir D. Association of Implementation of a Universal Testing and Treatment Intervention With HIV Diagnosis, Receipt of Antiretroviral Therapy, and Viral Suppression in East Africa. JAMA. 2017;317(21):2196-2206. PubMed PMID: 28586888; PubMed Central PMCID: PMCPMC5734234.

11. Grobler A, Cawood C, Khanyile D, Puren A, Kharsany ABM. Progress of UNAIDS 90-90-90 targets in a district in KwaZulu-Natal, South Africa, with high HIV burden, in the HIPSS study: a household-based complex multilevel community survey. The lancet HIV. 2017;4(11):e505-e513. PubMed PMID: 28779855.

12. Huerga H, Van Cutsem G, Ben Farhat J, Puren A, Bouhenia M, Wiesner L, Dlamini L, Maman D, Ellman T, Etard JF. Progress towards the UNAIDS 90-90-90 goals by age and gender in a rural area of KwaZulu-Natal, South Africa: a household-based community cross-sectional survey. BMC Public Health. 2018;18(1):303. PubMed PMID: 29499668; PubMed Central PMCID: PMCPMC5833029.

13. Floyd S, Ayles H, Schaap A, Shanaube K, MacLeod D, Phiri M, Griffith S, Bock P, Beyers N, Fidler S, Hayes R, Team HS. Towards 90-90: Findings after two years of the HPTN 071 (PopART) cluster-randomized trial of a universal testing-and-treatment intervention in Zambia. PLoS ONE. 2018;13(8):e0197904. PubMed PMID: 30096139; PubMed Central PMCID: PMCPMC6086421.

14. Larmarange J, Diallo MH, McGrath N, Iwuji C, Plazy M, Thiebaut R, Tanser F, Barnighausen T, Pillay D, Dabis F, Orne-Gliemann J, Group ATS. The impact of population dynamics on the population HIV care cascade: results from the ANRS 12249 Treatment as Prevention trial in rural KwaZulu-Natal (South Africa). Journal of the International AIDS Society. 2018;21 Suppl 4:e25128. PubMed PMID: 30027600; PubMed Central PMCID: PMCPMC6053480.

15. Edwards JK, Arimi P, Ssengooba F, Mulholland G, Markiewicz M, Bukusi EA, Orikiiriza JT, Virkud A, Weir S. The HIV care continuum among resident and non-resident populations found in venues in East Africa cross-border areas. Journal of the International AIDS Society. 2019;22(1):e25226. PubMed PMID: 30675984; PubMed Central PMCID: PMCPMC6344908.

16. Puryear SB, Balzer LB, Ayieko J, Kwarisiima D, Hahn JA, Charlebois ED, Clark TD, Cohen CR, Bukusi EA, Kamya MR, Petersen ML, Havlir DV, Chamie G. Associations between alcohol use and HIV care cascade outcomes among adults undergoing population-based HIV testing in East Africa. Aids. 2019. PubMed PMID: 31725431.

17. Velloza J, Kemp CG, Aunon FM, Ramaiya MK, Creegan E, Simoni JM. Alcohol Use and Antiretroviral Therapy Non-Adherence Among Adults Living with HIV/AIDS in Sub-Saharan Africa: A Systematic Review and Meta-Analysis. AIDS and behavior. 2019. PubMed PMID: 31673913.

18. San Francisco HIV Epidemiology: Annual Report 2017. San Francisco Department of Public Health, Population Health Division. HIV Epidemiology Section. 2018.

19. Grulich AE, Guy R, Amin J, Jin F, Selvey C, Holden J, Schmidt HA, Zablotska I, Price K, Whittaker B, Chant K, Cooper C, McGill S, Telfer B, Yeung B, Levitt G, Ogilvie EE, Dharan NJ, Hammoud MA, Vaccher S, Watchirs-Smith L, McNulty A, Smith DJ, Allen DM, Baker D, Bloch M, Bopage RI, Brown K, Carr A, Carmody CJ, Collins KL, Finlayson R, Foster R, Jackson EY, Lewis DA, Lusk J, O'Connor CC, Ryder N, Vlahakis E, Read P, Cooper DA, Expanded Pr EPIiCNSWrg. Population-level effectiveness of rapid, targeted, high-coverage roll-out of HIV pre-exposure prophylaxis in men who have sex with men: the EPIC-NSW prospective cohort study. The lancet HIV. 2018;5(11):e629-e637. PubMed PMID: 30343026.

20. https://www.prepwatch.org/resource/global-prep-tracker/. Last accessed April 2019. 2019.

21. Hodges-Mameletzis I, Dalal S, Msimanga-Radebe B, Rodolph M, Baggaley R. Going global: the adoption of the World Health Organization's enabling recommendation on oral pre-exposure prophylaxis for HIV. Sex Health. 2018;15(6):489-500. PubMed PMID: 30496718.

22. Koss CA, Ayieko J, Mwangwa F, Owaraganise A, Kwarisiima D, Balzer LB, Plenty A, Sang N, Kabami J, Ruel TD, Black D, Camlin CS, Cohen CR, Bukusi EA, Clark TD, Charlebois ED, Petersen ML, Kamya MR, Havlir DV, study S. Early Adopters of HIV Preexposure Prophylaxis in a Population-based Combination Prevention Study in Rural Kenya and Uganda. Clin Infect Dis. 2018. PubMed PMID: 29741594.

23. Eakle R, Venter F, Rees H. Pre-exposure prophylaxis (PrEP) in an era of stalled HIV prevention: Can it change the game? Retrovirology. 2018;15(1):29. PubMed PMID: 29609619; PubMed Central PMCID: PMCPMC5879931.

24. Haberer JE. Current concepts for PrEP adherence in the PrEP revolution: from clinical trials to routine practice. Current opinion in HIV and AIDS. 2016;11(1):10-17. PubMed PMID: 26633638; PubMed Central PMCID: PMCPMC4801217.

25. Lindberg L, Santelli J, Desai S. Understanding the Decline in Adolescent Fertility in the United States, 2007-2012. J Adolesc Health. 2016;59(5):577-583. PubMed PMID: 27595471; PubMed Central PMCID: PMCPMC5498007.

26. WHO. Family Planning Evidence Brief – Expanding contraceptive choice WHO/RHR/17.14 Rev.1. Available at: https://www.who.int/reproductivehealth/publications/family_planning/expanding-contraceptive-choice/en/. 2017.

27. Nel A, van Niekerk N, Kapiga S, Bekker LG, Gama C, Gill K, Kamali A, Kotze P, Louw C, Mabude Z, Miti N, Kusemererwa S, Tempelman H, Carstens H, Devlin B, Isaacs M, Malherbe M, Mans W, Nuttall J, Russell

M, Ntshele S, Smit M, Solai L, Spence P, Steytler J, Windle K, Borremans M, Resseler S, Van Roey J, Parys W, Vangeneugden T, Van Baelen B, Rosenberg Z, Ring Study T. Safety and Efficacy of a Dapivirine Vaginal Ring for HIV Prevention in Women. N Engl J Med. 2016;375(22):2133-2143. PubMed PMID: 27959766.

28. Baeten JM, Palanee-Phillips T, Brown ER, Schwartz K, Soto-Torres LE, Govender V, Mgodi NM, Matovu Kiweewa F, Nair G, Mhlanga F, Siva S, Bekker LG, Jeenarain N, Gaffoor Z, Martinson F, Makanani B, Pather A, Naidoo L, Husnik M, Richardson BA, Parikh UM, Mellors JW, Marzinke MA, Hendrix CW, van der Straten A, Ramjee G, Chirenje ZM, Nakabiito C, Taha TE, Jones J, Mayo A, Scheckter R, Berthiaume J, Livant E, Jacobson C, Ndase P, White R, Patterson K, Germuga D, Galaska B, Bunge K, Singh D, Szydlo DW, Montgomery ET, Mensch BS, Torjesen K, Grossman CI, Chakhtoura N, Nel A, Rosenberg Z, McGowan I, Hillier S, Team M-AS. Use of a Vaginal Ring Containing Dapivirine for HIV-1 Prevention in Women. N Engl J Med. 2016;375(22):2121-2132. PubMed PMID: 26900902; PubMed Central PMCID: PMCPMC4993693.

29. Delany-Moretlwe S, Hughes JP, Bock P, Ouma SG, Hunidzarira P, Kalonji D, Kayange N, Makhema J, Mandima P, Mathew C, Spooner E, Mpendo J, Mukwekwerere P, Mgodi N, Ntege PN, Nair G, Nakabiito C, Nuwagaba-Biribonwoha H, Panchia R, Singh N, Siziba B, Farrior J, Rose S, Anderson PL, Eshleman SH, Marzinke MA, Hendrix CW, Beigel-Orme S, Hosek S, Tolley E, Sista N, Adeyeye A, Rooney JF, Rinehart A, Spreen WR, Smith K, Hanscom B, Cohen MS, Hosseinipour MC; **HPTN 084 study group. Cabotegravir for the prevention of HIV-1 in women: results from HPTN 084, a phase 3, randomised clinical trial.** *Lancet*. 2022 May 7;399(10337):1779-1789. PMID: 35378077.

30. Landovitz RJ, Li S, Eron JJ Jr, Grinsztejn B, Dawood H, Liu AY, Magnus M, Hosseinipour MC, Panchia R, Cottle L, Chau G, Richardson P, Marzinke MA, Eshleman SH, Kofron R, Adeyeye A, Burns D, Rinehart AR, Margolis D, Cohen MS, McCauley M, Hendrix CW. **Tail-phase safety, tolerability, and pharmacokinetics of long-acting injectable cabotegravir in HIV-uninfected adults: a secondary analysis of the HPTN 077 trial.** Lancet HIV. 2020 Jul;7(7):e472-e481. doi: 10.1016/S2352-3018(20)30106-5. Epub 2020 Jun 1. PMID: 32497491.

31. Landovitz RJ, Donnell D, Clement ME, Hanscom B, Cottle L, Coelho L, Cabello R, Chariyalertsak S, Dunne EF, Frank I, Gallardo-Cartagena JA, Gaur AH, Gonzales P, Tran HV, Hinojosa JC, Kallas EG, Kelley CF, Losso MH, Madruga JV, Middelkoop K, Phanuphak N, Santos B, Sued O, Valencia Huamaní J, Overton ET, Swaminathan S, Del Rio C, Gulick RM, Richardson P, Sullivan P, Piwowar-Manning E, Marzinke M, Hendrix C, Li M, Wang Z, Marrazzo J, Daar E, Asmelash A, Brown TT, Anderson P, Eshleman SH, Bryan M, Blanchette C, Lucas J, Psaros C, Safren S, Sugarman J, Scott H, Eron JJ, Fields SD, Sista ND, Gomez-Feliciano K, Jennings A, Kofron RM, Holtz TH, Shin K, Rooney JF, Smith KY, Spreen W, Margolis D, Rinehart A, Adeyeye A, Cohen MS, McCauley M, Grinsztejn B; HPTN 083 Study Team. **Cabotegravir for HIV Prevention in Cisgender Men and Transgender Women.** *N Engl J Med.* 2021 Aug 12;385(7):595-608. PMID: 34379922.

32. Eshleman SH, Fogel JM, Piwowar-Manning E, Chau G, Cummings V, Agyei Y, Richardson P, Sullivan P, Haines CD, Bushman LR, Petropoulos C, Persaud D, Kofron R, Hendrix CW, Anderson PL, Farrior J, Mellors J, Adeyeye A, Rinehart A, St Clair M, Ford S, Rooney JF, Mathew CA, Hunidzarira P, Spooner E, Mpendo J, Nair G, Cohen MS, Hughes JP, Hosseinipour M, Hanscom B, Delany-Moretlwe S, Marzinke MA. **Characterization of Human Immunodeficiency Virus (HIV) Infections in Women Who Received Injectable Cabotegravir or Tenofovir Disoproxil Fumarate/Emtricitabine for HIV Prevention: HPTN 084.** *J Infect Dis.* 2022 Mar 18:jiab576. Epub ahead of print. PMID: 35301540.

33. Marzinke MA, Grinsztejn B, Fogel JM, Piwowar-Manning E, Li M, Weng L, et al. **Characterization of HIV infection in cisgender men and transgender women who have sex with men receiving injectable cabotegravir for HIV prevention: HPTN 083.** *J Infect Dis.* 2021 Nov 16;224(9):1581-1592. PMID: **33740057.**

34. Markowitz M, Frank I, Grant RM, Mayer KH, Elion R, Goldstein D, et al**. Safety and tolerability of long-acting cabotegravir injections in HIV-uninfected men (ECLAIR): a multicentre, double-blind, randomised, placebo-controlled, Bhase 2a trial.** *Lancet HIV.* 2017 Aug;4(8):e331-e40. PMID: 28546090.

35. Eshleman S, Fogel JM, Halvas EK, Mellors JW, Piwowar-Manning EM, Rinehart AR, et al. **CAB-LA PrEP: Early Detection of HIV Infection May Reduce In STI Resistance Risk.** *Virtual Abstract presented at Retroviruses and Opportunistic Infections Confernce*, *February 12-16, 2022.*

36. Delany-Moretlwe S, Hughes J, Guo X, Hanscom B, Hendrix CW, Farrior J, et al. **Evaluation of CAB-LA Safety and PK in Pregnant Women in the Blinded Phase of HPTN 084. Abstract 700.** *Virtual Conference presented at Retroviruses and Opportunistic Infections Confernce, February 12-16, 2022.*

37. Blair CS, Li S, Chau G, Cottle L, Richardson P, Marzinke MA, Eshleman SH, Adeyeye A, Rinehart AR, Margolis D, McCauley M, Hendrix CW, Landovitz RJ; **HPTN 077 Study Team. Brief Report: Hormonal Contraception Use and Cabotegravir Pharmacokinetics in HIV-Uninfected Women Enrolled in HPTN 077.** *J Acquir Immune Defic Syndr.* 2020 Sep 1;85(1):93-97. PMID: 32452972.

38. National AIDS and STI Control Programme, Kenya Medical Research Institute. **Guidelines for Conducting Adolescent HIV Sexual and Reproductive Health Research in Kenya;** 2015.

39. Uganda National Council for Science and Technology. **National Guidelines for Researchinvolving Humans as Research Participants;** 2014.

40. Delany-Moretlwe S, Hughes JP, Bock P, Dadabhai S, Hunidzarira P, Innes S, Kalonji D, Makhema J, Mandima P, Mathew C, Mpendo J, Mukwekwerere P, Mgodi N, Nahira Ntege P, Nakabiito C, Nuwagaba-Biribonwoha H, Panchia R, Angria F, Singh N, Siziba B, Spooner E, Farrior J, Rose S, Berhanu R, Agyei Y, Eshleman SH, Marzinke MA, Piowar-Manning E, Beigel-Orme S, Hosek S, Adeyeye A, Rooney J, Rinehart A, Hanscom B, Cohen MS, Hosseinipour MC. Long Acting Injectable Cabotegravir: Updated Efficacy and Safety Results from HPTN 084. AIDS 2022 July 29 - Aug 2; 24th Intl AIDS Conference; 2022. Accessed August 1, 2022. https://www.natap.org/2022/IAC/IAC_22.htm

APPENDIX B: CONSORT 2010 CHECKLIST OF INFORMATION TO INCLUDE WHEN REPORTING A RANDOMISED TRIAL*

Section/Topic	Item No	Checklist item	Reported on page No
**Title and abstract**			
	1a	Identification as a randomised trial in the title	1
	1b	Structured summary of trial design, methods, results, and conclusions (for specific guidance see CONSORT for abstracts)	3-4
**Introduction**			
Background and objectives	2a	Scientific background and explanation of rationale	5-6
	2b	Specific objectives or hypotheses	6
**Methods**			
Trial design	3a	Description of trial design (such as parallel, factorial) including allocation ratio	7
	3b	Important changes to methods after trial commencement (such as eligibility criteria), with reasons	NA
Participants	4a	Eligibility criteria for participants	6
	4b	Settings and locations where the data were collected	6
Interventions	5	The interventions for each group with sufficient details to allow replication, including how and when they were actually administered	7-9
Outcomes	6a	Completely defined pre-specified primary and secondary outcome measures, including how and when they were assessed	9-10 (details in SAP)
	6b	Any changes to trial outcomes after the trial commenced, with reasons	NA
Sample size	7a	How sample size was determined	10 (details in SAP)
	7b	When applicable, explanation of any interim analyses and stopping guidelines	NA
Randomisation:			
Sequence generation	8a	Method used to generate the random allocation sequence	7 (details in ref #5-7)
	8b	Type of randomisation; details of any restriction (such as blocking and block size)	7 (details in ref #5-7)
Allocation concealment mechanism	9	Mechanism used to implement the random allocation sequence (such as sequentially numbered containers), describing any steps taken to conceal the sequence until interventions were assigned	7 (details in ref #5-7)
Implementation	10	Who generated the random allocation sequence, who enrolled participants, and who assigned participants to interventions	7 (details in ref #5-7)
Blinding	11a	If done, who was blinded after assignment to interventions (for example, participants, care providers, those assessing outcomes) and how	7 (details in ref #5-7; SAP)
	11b	If relevant, description of the similarity of interventions	NA
Statistical methods	12a	Statistical methods used to compare groups for primary and secondary outcomes	10-11 (details in SAP)
	12b	Methods for additional analyses, such as subgroup analyses and adjusted analyses	10-11 (details in SAP)
**Results**			
Participant flow (a diagram is strongly recommended)	13a	For each group, the numbers of participants who were randomly assigned, received intended treatment, and were analysed for the primary outcome	Figure 1
	13b	For each group, losses and exclusions after randomisation, together with reasons	Figure 1
Recruitment	14a	Dates defining the periods of recruitment and follow-up	11-12
	14b	Why the trial ended or was stopped	NA
Baseline data	15	A table showing baseline demographic and clinical characteristics for each group	Table 1
Numbers analysed	16	For each group, number of participants (denominator) included in each analysis and whether the analysis was by original assigned groups	11-12 & Table 2
Outcomes and estimation	17a	For each primary and secondary outcome, results for each group, and the estimated effect size and its precision (such as 95% confidence interval)	12-15 & Figure 3 & Supp Materials

	17b	For binary outcomes, presentation of both absolute and relative effect sizes is recommended	NA
Ancillary analyses	18	Results of any other analyses performed, including subgroup analyses and adjusted analyses, distinguishing pre-specified from exploratory	12-15 & Supp Materials
Harms	19	All important harms or unintended effects in each group (for specific guidance see CONSORT for harms)	NA
**Discussion**			
Limitations	20	Trial limitations, addressing sources of potential bias, imprecision, and, if relevant, multiplicity of analyses	18
Generalisability	21	Generalisability (external validity, applicability) of the trial findings	14-19
Interpretation	22	Interpretation consistent with results, balancing benefits and harms, and considering other relevant evidence	14-19
**Other information**			
Registration	23	Registration number and name of trial registry	abstract; 11
Protocol	24	Where the full trial protocol can be accessed, if available	10
Funding	25	Sources of funding and other support (such as supply of drugs), role of funders	9-10, 20

Citation: Schulz KF, Altman DG, Moher D, for the CONSORT Group. CONSORT 2010 Statement: updated guidelines for reporting parallel group randomised trials. BMC Medicine. 2010;8:18.
© 2010 Schulz et al. This is an Open Access article distributed under the terms of the Creative Commons Attribution License (http://creativecommons.org/licenses/by/2.0), which permits unrestricted use, distribution, and reproduction in any medium, provided the original work is properly cited.

*We strongly recommend reading this statement in conjunction with the CONSORT 2010 Explanation and Elaboration for important clarifications on all the items. If relevant, we also recommend reading CONSORT extensions for cluster randomised trials, non-inferiority and equivalence trials, non-pharmacological treatments, herbal interventions, and pragmatic trials. Additional extensions are forthcoming: for those and for up-to-date references relevant to this checklist, see www.consort-statement.org.

APPENDIX C: STATISTICAL ANALYSIS PLAN

Sustainable East Africa Research in Community Health
(SEARCH) Collaboration

Statistical Analysis Plan for the
SEARCH "Dynamic Choice HIV Prevention" Extension Study including the Option of Long Acting Cabotegravir Injectable Suspension (CAB-LA) in rural Kenya and Uganda

Laura B. Balzer, PhD, Study Statistician[1]
Diane V. Havlir, MD, co-Principal Investigator[2]
Moses R. Kamya, MD, PhD co-Principal Investigator [3-4]
Maya L. Petersen, MD, PhD co-Principal Investigator[1]
for the SEARCH Collaboration[5]

This document provides the statistical analytic plan (SAP) for the evaluation of the effect of the SEARCH Dynamic Choice HIV Prevention intervention, including the option of CAB-LA, on biomedical prevention coverage (primary outcome) as well as HIV incidence and biomedical prevention coverage during periods of self-reported HIV risk (secondary outcomes) in a randomized trial extension study in rural Kenya and Uganda (Clinicaltrials.gov: NCT05549726). The SAP was locked prior to unblinding and effect estimation.

December 15, 2023
v1.1

[1] Division of Biostatistics, University of California Berkeley, Berkeley, USA
[2] Division of HIV, Infectious Diseases, and Global Medicine, University of California San Francisco, San Francisco, USA
[3] Infectious Diseases Research Collaboration, Kampala, Uganda
[4] Department of Medicine, Makerere University College of Health Sciences, Kampala, Uganda
[5] www.searchendaids.com

1. OVERVIEW OF TTHE SEARCH CAB-LA EXTENSION STUDY

This study is testing the hypothesis that a "Dynamic Choice HIV Prevention" intervention that offers participants CAB-LA, oral Pre-Exposure Prophylaxis (PrEP), or post-exposure prophylaxis (PEP) and the ability to switch between products over time using a patient-centered delivery approach will achieve higher prevention coverage for males and females ≥15 years of age and at risk for HIV infection acquisition, as compared to the country standard-of-care (SoC). The current SoC for biomedical HIV prevention includes oral PrEP and PEP. This study is being conducted in rural western Uganda and rural western Kenya. The follow-up is over 48 weeks. Additional details of the study design, arms, and procedures can be found in the Study Protocol.
The primary objective is to evaluate the effect of the Dynamic Choice HIV Prevention intervention versus the SoC on biomedical HIV prevention coverage, defined as the proportion of follow-up time covered by either PrEP (oral PrEP or CAB-LA) or PEP. Secondary, pre-specified endpoints compared between arms and included in this SAP are coverage during periods of self-reported risk of HIV acquisition ("at-risk coverage") and HIV incidence. We additionally describe product use over time between arms. Analysis plans for other endpoints (e.g., implementation metrics and the change in endpoints before and after adding CAB-LA to the intervention) are available elsewhere. Power calculations and history of changes are given in the Appendix.

2. POPULATION AND CHARACTERISTICS

The study population is persons who were enrolled in the 3 randomized Dynamic Choice HIV Prevention trials that recruited from antenatal clinics, outpatient departments, and the community.[1–3] These trials were designed to evaluate the impact of the Dynamic Choice HIV Prevention intervention (offering choice between oral PrEP and

PEP as biomedical HIV prevention options) versus the SoC on biomedical HIV prevention coverage over 48 weeks (**Figure 1**).

Figure 1: Schematic of the CAB-LA extension study with abbreviations of DCP for Dynamic Choice HIV Prevention intervention and SOC for standard-of-care.

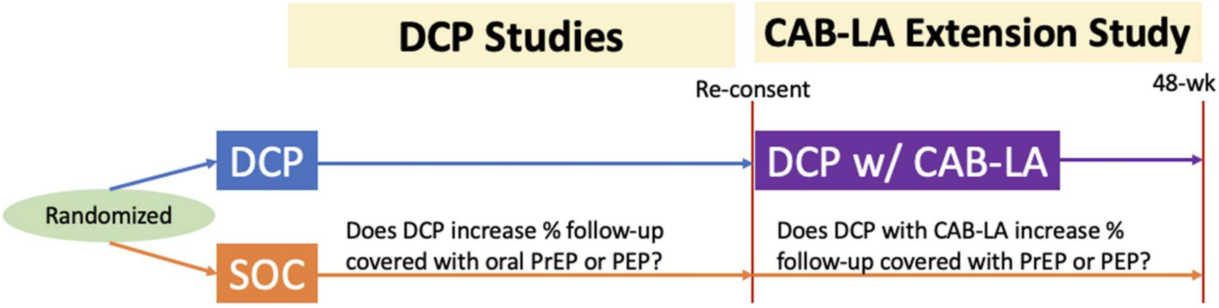

The Dynamic Choice HIV Prevention trials enrolled participants aged ≥15 years with current or anticipated risk to acquire HIV. Inclusion criteria for the CAB-LA extension study are
1) Prior enrollment in a SEARCH Dynamic Choice HIV Prevention trial,
2) HIV-negative by country-standard rapid HIV testing algorithm, and
3) Residence in the study region.
In the extension, participants maintained their original randomization arm and are not blinded to the treatment arm. The primary statistician (LBB) will remain blinded to the treatment arm until the official unblinding of the study when the primary and secondary effect analyses, described in this SAP, are conducted. Additional details, including randomization procedures and eligibility for CAB-LA access among intervention arm participants, are in the Study Protocol.
To characterize participant flow from original randomization through measurement of the primary endpoint of the extension, we will provide a CONSORT diagram. To describe the study population, we will summarize participant characteristics at start of the extension, overall and by arm. These characteristics will include recruitment setting, country, sex, age, marital status, occupation, pregnancy (women only), circumcision (men only), and alcohol use (having 1+ alcoholic drink per week).

3. ENDPOINT DEFINITION AND MEASUREMENT

3.1. Biomedical HIV Prevention Coverage

The primary endpoint of this study is biomedical HIV prevention coverage, defined as the proportion of follow-up time covered by either PrEP (TDF/3TC or CAB-LA) or PEP. Thereby, this endpoint has a minimum of 0% (no use) and a maximum of 100% (full coverage). For oral PrEP and PEP, coverage is defined using self-report via a structured survey administered at 24 and 48 weeks of follow-up. Specifically, for each month of the prior 6 months, participants retrospectively report any use of oral PrEP (i.e., pill ingestion) and any use of PEP. For CAB-LA, coverage is defined through injection logs. Coverage from CAB-LA begins 3 days after an injection and continues for 67 days.

3.2 At-Risk Coverage

A secondary endpoint of this study is at-risk coverage, defined as the proportion of follow-up time that a participant reports being at risk of HIV acquisition during which they are covered by a biomedical prevention option. Product use over time will be defined analogously to the primary outcome. Self-reported risk of HIV acquisition will be assessed retrospectively via the structured survey administered at 24 and 48 weeks of follow-up.

3.3 Incident HIV Acquisition

A secondary endpoint of this study is incident HIV acquisition. HIV rapid antibody testing and HIV RNA testing (Cepheid GeneXpert Diagnostic System, Sunnyvale, California) will be performed at baseline, week-24, and week-48. HIV testing is also available at the Ministry of Health clinics at all study sites. All participants with detectable HIV RNA and negative rapid antibody testing will have repeat antibody testing for confirmation of HIV diagnosis. All possible incident HIV infections will be reviewed by two HIV clinical experts blinded to the trial arm to determine if the participant meets the definition of confirmed incident HIV infection.

4. EVALUATION OF THE EFFECT OF THE SEARCH DYNAMIC CHOICE HIV PREVENTION INTERVENTION WITH CAB-LA

4.1. Primary Analysis: Effect on Biomedical HIV Prevention Coverage

The primary endpoint of biomedical HIV prevention coverage will be compared between arms with **targeted minimum loss-based estimation (TMLE) to improve efficiency over the unadjusted effect estimator**.[4–12] Briefly, TMLE combines estimates of the outcome regression (i.e., the conditional expectation of the outcome, given the intervention and covariates) and the known propensity score (i.e., the conditional probability of the intervention, given the covariates) to obtain doubly-robust inference for the estimand of interest.
The primary analysis will adjust for recruitment setting (antenatal clinic, outpatient department, or community) in the outcome regression and adaptively adjust for additional baseline covariates to optimize precision, while preserving Type-I error control. Specifically, we will use **Adaptive Pre-specification** to select the candidate TMLE that maximizes empirical efficiency.[8,10,12] Using 10-fold cross-validation, we will select from the following pre-specified candidates:

- Outcome regression estimators: generalized linear models (GLMs) adjusting for a single covariate (beyond setting), main terms GLM, LASSO, or no further adjustment.
- Propensity score estimators: GLMs adjusting for a single covariate, main terms GLM, LASSO, or no adjustment
- Candidate variables: sex, age, alcohol use, mobility, or nothing

The primary analysis will estimate the marginal effect on the absolute scale (i.e., the difference in the average of the arm-specific outcomes). Secondary comparisons will be on the ratio scale. Standard error estimation will be based on the estimated influence curve, accounting for clustering from the community-based setting.[9,10,13–15] Statistical inference will follow from the Central Limit Theorem.[4] Specifically, we will create Wald-type 95% confidence intervals and test the **null hypothesis** of no difference in biomedical HIV prevention coverage with a two-sided test at the 5% significance level. We will also report point estimates and 95% confidence intervals for the arm-specific estimates.
In **sensitivity analyses**, we will assess the robustness of our findings by only adjusting for setting in the outcome regression as well as implementing the unadjusted effect estimator. We may conduct an additional sensitivity analysis adjusting for, instead of excluding, participants with zero follow-up. Additionally, we will evaluate the effect on coverage when restricting follow-up time to periods included in the structured survey administered to assess use of oral PrEP or PEP.
In additional sensitivity analyses, we will use data from the initial trials through the extension and apply TMLE to adjust for differences between participants who did vs. did not continue in the extension and participants who did vs. did not have their endpoint ascertained. See Section 6 for details.
We will repeat these analyses within **subgroups** defined by sex and age group (15-24 years vs. 25+ years) and apply the Bonferroni correction for multiple testing. To further understand effect heterogeneity, we may conduct variable importance measures (i.e., unadjusted and adjusted predictor analyses) with TMLE.
Prior to locking this SAP, we will conduct treatment-blind simulations to verify Type-I error control for all endpoints.[12]

4.2. Secondary Endpoint Analysis: Effect on At-Risk Coverage

We will implement analogous analyses to assess the SEARCH effect on coverage during periods of self-reported HIV risk. We will use the same approach for effect estimation, statistical inference, and multiple testing. We will conduct analogous subgroup and sensitivity analyses.

4.3. Secondary Endpoint Analysis: Effect on HIV Incidence

A key secondary endpoint is incident HIV infection. For each participant, we will calculate person-time-at-risk (PT) as the difference between enrollment into the extension and

- Last negative HIV test for persons who did not acquire HIV
- Imputed HIV infection date, defined as the midpoint between last negative and first positive test, for persons who did acquire HIV.

We will then compare the HIV incidence rate, defined as the sum of incident infections divided by the sum of PT, between randomized arms with the unadjusted effect estimator. Due to the anticipated rarity of the outcome, we will not apply TMLE for additional precision gains.
Our approach to inference will be implemented analogously to the primary endpoint.
As before, the primary comparison will be on the absolute scale (i.e., the difference in the incidence rates between arms) with secondary comparisons on the relative scale. Again, standard error estimation will be based on the estimated influence curve, accounting for clustering and inference based on the Central Limit Theorem. We will test the null hypothesis of no difference in HIV incidence rates with a two-sided test at the 5% significance level. In sensitivity analyses, we will, instead, use the non-parametric bootstrap for standard error estimation and exact tests for inference.
We will repeat these analyses within groups defined by sex and age group. However, we will not conduct hypothesis testing for these groups.

5. ADDITIONAL DESCRIPTIVE ANALYSES

We will summarize participant follow-up, overall and by arm. Follow-up in the CAB-LA extension study starts at enrollment and ends at the earliest of HIV diagnosis, death, withdrawal, or December 31, 2023. Follow-up time is censored during periods without data on use of biomedical HIV prevention.
Overall and by arm, we will report the number and proportion of participants who withdrew, died, or had other adverse events. We may also report on outcomes among women who become pregnant after initiating CAB-LA.
We will summarize data on biomedical HIV prevention product use by arm. These summaries will exclude participants without any follow-up data on biomedical prevention use. We will use heatmaps to depict participant-level use of prevention products over months of follow-up in the extension. We will also provide descriptive statistics about product use over time, including the month prior to the extension study, at baseline in the extension study, and ever use during the extension study. When calculating these descriptive statistics, missing values at baseline product use will be imputed from the first follow-up month.

6. SENSITIVITY ANALYSES

In this extension study, participants will maintain their original randomization arm. As a result, there is a legitimate concern that arms will be imbalanced at the start of the extension study. We will address this concern by conducting the following sensitivity analyses for the effect on biomedical HIV prevention coverage (primary endpoint) and at-risk coverage (secondary endpoint). These analyses will incorporate data from all persons who participated in the 3 Dynamic Choice HIV Prevention trials – regardless if they continued in the extension and/or had their endpoint ascertained in the extension.
This sensitivity analysis is informed by the Causal Roadmap[16] and aims address possible bias due to selection and missing data. Specifically, we can frame the analysis as asking, “What is the effect of the SEARCH Dynamic Choice HIV Prevention intervention (including the option of CAB-LA) if all participants had continued in the extension and had their endpoint ascertained?” While the treatment arm is randomized, we need to make the following missing data assumption: within values of adjustment variables, participants with their endpoint measured in the extension (and, thus, continued in the extension) are representative of participants who do not. Here, our

adjustment set includes variables measured at recruitment into the initial trials (hereafter "baseline"): age, sex, country, recruitment site, alcohol use, and mobility. Importantly, our adjustment set also includes trial arm as well as post-intervention variables measured prior to the extension start: biomedical HIV prevention coverage, at-risk coverage, follow-up time, and follow-up time with self-reported risk of HIV acquisition. We additionally need that there is a positive probability of endpoint ascertainment within all possible values of these adjustment variables. Under these assumptions, our statistical estimand is given by the Longitudinal G-computation formula:[17]

$$\mathbb{E}\{\mathbb{E}[\mathbb{E}(Y|\Delta = 1, L1, A = 1, L0)|A = 1, L0]\} - \mathbb{E}\{\mathbb{E}[\mathbb{E}(Y|\Delta = 1, L1, A = 0, L0)|A = 0, L0]\}$$

where Y is the endpoint, Δ is an indicator of having that endpoint measured and, thus, continuing in the extension, $L1$ are the time-varying covariates, A is an indicator of being randomized to the intervention arm, and $L0$ are the baseline covariates.

For estimation and inference, we will use longitudinal TMLE as implemented in the *ltmle* package.[4,14,18] In the main analysis, we will employ Super Learner, which is an ensemble machine learning method, to flexibly adjust and to improve efficiency.[19] Within Super Learner, we will combine predictions from main terms logistic regression (GLM), generalized additive models (GAM), multivariate adaptive splines (MARS), and the mean. The main analysis will also bound the estimated propensity scores (for measurement) from below at 1%. We will use the same approach for statistical inference and multiple testing as the primary analysis.

We will further examine the sensitivity to our assumptions and analytic choices by implementing TMLE as follows:

- TMLE with Super Learner (as described above) but bounding the estimated propensity scores from below at 2%
- TMLE with main terms logistic regressions (instead of Super Learner)
- TMLE with Super Learner but only adjusting for baseline variables
- TMLE with main terms logistic regressions and only adjusting for baseline variables

We will implement these analyses for biomedical HIV prevention coverage (primary endpoint) and at-risk coverage, overall and within the predefined subgroups.

7. POWER CALCULATIONS

We assume 70% of participants in the 3 Dynamic Choice HIV Prevention trials enroll in the extension. Of those, we assume 90% have measures of biomedical HIV prevention coverage (the primary endpoint). Then using standard formulas for continuous outcomes with clustering,[20,21] we anticipate at least 80% power to detect at least a 7.5% absolute increase in prevention coverage with 485 participants/arm, an outcome standard deviation of 0.35, and a design effect of 1.3. As shown in Figure 2, these calculations are fairly robust to alternative assumptions, including lower outcome measurement and higher design effects. We further expect these calculations to be conservative given the precision gains due to covariate adjustment with TMLE in the primary analysis.

Figure 2: Minimal effect size (difference) detected with 80% power with a two-sample t-test (via power.t.test in R) after deflating the sample size by the design effect

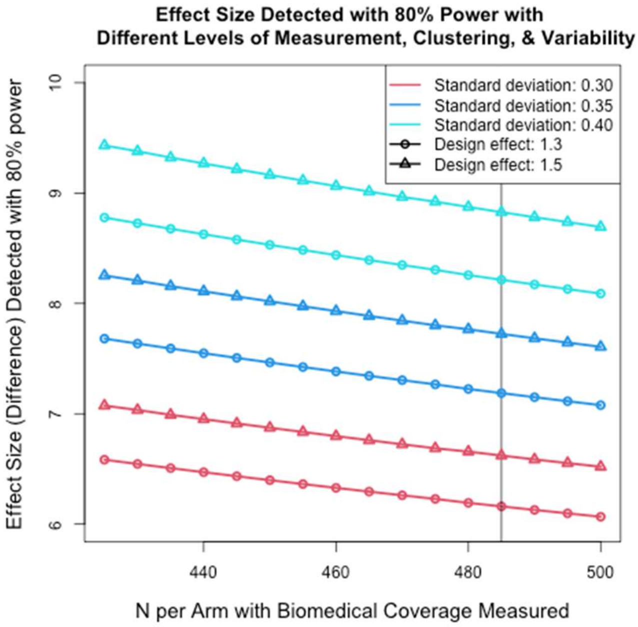

8. HISTORY OF CHANGES

- Version 1.0: This version includes updated power calculations (as compared to the Study Protocol v7.0) to reflect enrollment into the extension and that the primary analysis pools over and adjusts for (versus stratifies on) recruitment setting.
- Version 1.1 (current version): This version includes additional details about the pre-specified sensitivity analyses (Section 5.0).

9. REFERENCES

1. Kabami J, Koss CA, Sunday H, et al. Randomized trial of dynamic choice HIV prevention at antenatal and postnatal care clinics in rural Uganda and Kenya. *JAIDS*. 2023;In Press.
2. Koss CA, Ayieko J, Kabami J, et al. Dynamic choice HIV prevention intervention at outpatient departments in rural Kenya and Uganda: a randomized trial. *AIDS*. Published online October 20, 2023. doi:10.1097/QAD.0000000000003763
3. Kakande ER, Ayieko J, Sunday H, et al. A community-based dynamic choice model for HIV prevention improves PrEP and PEP coverage in rural Uganda and Kenya: a cluster randomized trial. *J Int AIDS Soc*. 2023;26(12):e26195. doi:10.1002/jia2.26195
4. van der Laan M, Rose S. *Targeted Learning: Causal Inference for Observational and Experimental Data*. Springer; 2011.
5. Moore KL, van der Laan MJ. Covariate Adjustment in Randomized Trials with Binary Outcomes: Targeted Maximum Likelihood Estimation. *Statistics in Medicine*. 2009;28(1):39-64. doi:10.1002/sim.3445
6. Rosenblum M, Laan MJ van der. Simple, Efficient Estimators of Treatment Effects in Randomized Trials Using Generalized Linear Models to Leverage Baseline Variables. *The International Journal of Biostatistics*. 2010;6(1):Article 13. doi:10.2202/1557-4679.1138
7. Colantuoni E, Rosenblum M. Leveraging Prognostic Baseline Variables to Gain Precision in Randomized Trials. *Stat Med*. 2015;34(18):2602-2617.
8. Balzer L, van der Laan MJ, Petersen M, SEARCH Collaboration. Adaptive Pre-specification in Randomized

Trials With and Without Pair-Matching. *Statistics in Medicine*. 2016;35(10):4528-4545.

9. Havlir DV, Balzer LB, Charlebois ED, et al. HIV Testing and Treatment with the Use of a Community Health Approach in Rural Africa. *New England Journal of Medicine*. 2019;381(3):219-229.

10. Balzer LB, van der Laan M, Ayieko J, et al. Two-Stage TMLE to Reduce Bias and Improve Efficiency in Cluster Randomized Trials. *Biostatistics*. 2021;kxab043.

11. Benkeser D, Díaz I, Luedtke A, Segal J, Scharfstein D, Rosenblum M. Improving precision and power in randomized trials for COVID-19 treatments using covariate adjustment, for binary, ordinal, and time-to-event outcomes. *Biometrics*. 2021;77(4):1467-1481. doi:10.1111/biom.13377

12. Balzer LB, Cai E, Garraza LG, Amaranath P. Adaptive Selection of the Optimal Strategy to Improve Precision and Power in Randomized Trials. *Biometrics*. 2023;In Press.

13. Benitez A, Petersen ML, van der Laan MJ, et al. Defining and estimating effects in cluster randomized trials: A methods comparison. *Stat Med*. 2023;42(19):3443-3466. doi:10.1002/sim.9813

14. Petersen ML, Schwab J, Gruber S, Blaser N, Schomaker M, Laan MJ van der. Targeted Maximum Likelihood Estimation for Dynamic and Static Longitudinal Marginal Structural Working Models. *Journal of Causal Inference*. 2014;2(2). doi:10.1515/jci-2013-0007

15. Schnitzer ME, Laan MJ van der, Moodie EE, Platt RW. Effect of breastfeeding on gastrointestinal infection in infants: a targeted maximum likelihood approach for clustered longitudinal data. *Annals of Applied Statistics*. 2014;8(2):703-725.

16. Petersen ML, Laan MJ van der. Causal Models and Learning from Data: Integrating Causal Modeling and Statistical Estimation. *Epidemiology*. 2014;25(3):418-426.

17. Robins JM. A new approach to causal inference in mortality studies with sustained exposure periods–application to control of the healthy worker survivor effect. *Mathematical Modelling*. 1986;7:1393-1512. doi:10.1016/0270-0255(86)90088-6

18. Lendle SD, Schwab J, Petersen ML, van der Laan MJ. ltmle: An R Package Implementing Targeted Minimum Loss-based Estimation for Longitudinal Data. *Journal of Statistical Software*. 2017;81(1):1-21.

19. van der Laan MJ, Polley EC, Hubbard AE. Super learner. *Stat Appl Genet Mol Biol*. 2007;6:Article25. doi:10.2202/1544-6115.1309

20. Hayes RJ, Moulton LH. *Cluster Randomised Trials*. Chapman & Hall/CRC; 2009.

21. Eldridge SM, Ashby D, Kerry S. Sample size for cluster randomized trials: effect of coefficient of variation of cluster size and analysis method. *International Journal of Epidemiology*. 2006;35(5):1292-1300. doi:10.1093/ije/dyl129

APPENDIX D: TABLES AND FIGURES

TABLE S1. STUDY DYNAMIC CHOICE HIV PREVENTION INTERVENTION AND STANDARD OF CARE

Study Arm	Description
Standard of Care	• In the Standard of Care arm, there was self or community health worker referral for PrEP at the adjacent Minister of Health HIV clinic.<br>• Daily Oral PrEP was accessed according to country guidelines at the health facility. This included a 1-month medication supply at initiation and a 1- to 3-month supply at follow-up visits, with standard rapid blood-based HIV testing.<br>• PEP was not routinely available except in cases of gender-based violence or occupational exposure.<br>• CAB-LA was not available<br>• HIV self-testing, community-based visits, access to mobile health hotline, provider training in patient-centered care and offering structured choices of product over time were not included in Standard of Care.
Dynamic Choice HIV Prevention Intervention	• In the Dynamic Choice HIV Prevention intervention, there was self or community health worker referral for HIV prevention services at the adjacent Minister of Health HIV clinic.<br>• Participants choosing oral PrEP had the option of a 3 month medication supply provided at the initial visit.<br>• All participants had access to call or text a mobile health hot line 7 days a week.<br>• Training was provided for health center leadership, staff and providers, including case studies and discussion on the concept of dynamic choice HIV prevention, participant agency on decision making, development of personalized plans to overcome barriers to adherence and care, and education on the profile of each prevention option product.<br>• The study participant visits were integrated into antenatal clinics, outpatient department clinics, and in the community.<br>• Providers did a text or mobile phone check in with participants one week after starting HIV prevention option.<br>• Persons using the oral PrEP or PEP option could use HIV self -testing and were eligible for home delivery of product. For PEP, participants were provided “pill in pocket” to allow initiation immediately following exposure, and were instructed to call the mobile health hot line if they had questions, exposure, or initiated PEP.<br>• All CAB-LA injections were provided by health providers. There were no home visits for CAB LA injections.

TABLE S2. NUMBER AND PROPORTION WHO USED CAB-LA AT STUDY START, OVERALL AND BY BASELINE CHARACTERISTICS*

Overall	250/487 (51%)
Age group	
Age 15-24 years	73/139 (53%)
Age ≥ 25 years	177/348 (51%)
Sex	
Male	82/129 (64%)
Female[†]	168/358 (47%)
Country	
Kenyan	151/245 (62%)
Ugandan	99/242 (41%)
Recruitment setting	
Community	101/180 (56%)
Antenatal Clinic	70/165 (42%)
Outpatient Department	79/142 (56%)
Marital status	
Married or cohabitating	209/394 (53%)
Single (never married)	28/65 (43%)
Divorced, separated, or widowed	13/28 (46%)
Occupation	
Farmer	103/201 (51%)
Shopkeeper or market vendor	25/52 (48%)
Student	10/23 (43%)
Manual labor or construction	12/23 (52%)
Transportation	10/13 (77%)
Bar, hotel, or restaurant worker	5/13 (38%)
Fisher or fishmonger	5/9 (56%)
Alcohol use[‡]	
Yes	48/84 (57%)
No	202/403 (50%)
Circumcised[§]	
Yes	49/76 (64%)
No	33/53 (62%)
Pregnant[†,¶]	
Yes	0/32 (0%)
No	168/326 (52%)

[*]All metrics are number with the baseline characteristic using CAB-LA at the start of the extension study divided by the group size (%).

[†]Women who were pregnant were not eligible to initiate CAB-LA.

[‡]Alcohol use was defined as reporting drinking 1 or more alcoholic beverages per week.

[§]Summary statistics are for male participants.

[¶]Summary statistics are for female participants.

TABLE S3. PRIOR PRODUCT USE ON AMONG PERSONS USING CAB-LA*

	Started CAB-LA at Study Start	Used CAB-LA During the Study
Oral PrEP	142/250 (57%)	152/274 (55%)
PEP	3/250 (1%)	4/274 (1%)
No Product	105/250 (42%)	118/274 (43%)

* Prior refers to the month before the start of the extension study.

TABLE S4. SENSITIVITY ANALYSES FOR BIOMEDICAL HIV PREVENTION COVERAGE

The following sensitivity analyses were designed to assess the robustness of the primary endpoint findings when accounting for overall and by-arm differences between participants who did vs. did not enroll in the extension and participants who did vs. did not have their endpoint ascertained in the extension. See the Statistical Analysis Plan for details.

TABLE S4A. RESULTS FROM THE PRIMARY ANALYSIS AND THE MAIN SENSITIVITY ANALYSIS FOR BIOMEDICAL HIV PREVENTION COVERAGE, INCLUDING ARM-SPECIFIC ESTIMATES AND EFFECT ESTIMATES.

	Primary Analysis			Main Sensitivity Analysis		
	**Intervention (95%CI)**	**SoC (95%CI)**	**Difference (95%CI); p-value**	**Intervention (95%CI)**	**SoC (95%CI)**	**Difference (95%CI); p-value**
**Overall**	69.7% (64.9,74.5%)	13.3% (10.2,16.3%)	56.4% (50.8,62.1%); p<0.0001	71.0% (66.7,75.3%)	10.5% (7.8,13.2%)	60.5% (55.8,65.2%); p<0.0001
**Women**	68.3% (63.5,73.2%)	15.5% (12.0,19.1%)	52.8% (46.8,58.8%); p<0.0001	70.1% (65.1,75.1%)	13.4% (10.3,16.4%)	56.7% (51.3,62.1%); p<0.0001
**Men**	73.1% (65.5,80.7%)	7.5% (3.2,11.9%)	65.6% (56.9,74.4%); p<0.0001	70.5% (64.6,76.5%)	6.3% (3.1,9.4%)	64.3% (57.8,70.8%); p<0.0001
**15-24 years**	71.2% (64.9,77.6%)	12.2% (7.4,16.9%)	59.1% (51.2,67.0%); p<0.0001	73.4% (65.2,81.7%)	8.4% (5.1,11.8%)	65.0% (56.3,73.7%); p<0.0001
**25+ years**	69.2% (63.2,75.1%)	13.7% (10.1,17.4%)	55.4% (48.4,62.4%); p<0.0001	66.2% (61.0,71.4%)	12.3% (8.9,15.6%)	53.9% (48.1,59.8%); p<0.0001

TABLE S4B. ADDITIONAL SENSITIVITY ANALYSES FOR BIOMEDICAL HIV PREVENTION COVERAGE, SHOWN AS POINT ESTIMATE OF THE EFFECT WITH 95% CONFIDENCE INTERVALS AND RESULTING P-VALUE.

	**Primary Analysis[a]**	**TMLE with SL[b]**	**TMLE with SL & Bounding[c]**	**TMLE with GLMs[d]**	**TMLE with SL; baseline[e]**	**TMLE with GLMs; baseline[f]**
**Overall**	56.4% (50.8,62.1%); p<0.0001	60.5% (55.8,65.2%); p<0.0001	56.4% (49.4,63.4%); p<0.0001	56.4% (49.4,63.4%); p<0.0001	55.9% (50.7,61.1%); p<0.0001	55.8% (50.6,61.0%); p<0.0001
**Women**	52.8% (46.8,58.8%); p<0.0001	56.7% (51.3,62.1%); p<0.0001	54.0% (46.0,61.9%); p<0.0001	54.0% (46.0,61.9%); p<0.0001	52.7% (47.0,58.4%); p<0.0001	52.5% (46.8,58.2%); p<0.0001
**Men**	65.6% (56.9,74.4%); p<0.0001	64.3% (57.8,70.8%); p<0.0001	63.7% (53.5,73.8%); p<0.0001	63.7% (53.5,73.8%); p<0.0001	64.8% (56.4,73.2%); p<0.0001	64.5% (55.9,73.1%); p<0.0001
**15-24 years**	59.1% (51.2,67.0%); p<0.0001	65.0% (56.3,73.7%); p<0.0001	56.0% (43.2,68.9%); p<0.0001	57.1% (42.9,71.2%); p<0.0001	57.5% (49.8,65.2%); p<0.0001	57.3% (49.3,65.4%); p<0.0001
**25+ years**	55.4% (48.4,62.4%); p<0.0001	53.9% (48.1,59.8%); p<0.0001	52.7% (44.7,60.6%); p<0.0001	52.7% (44.7,60.6%); p<0.0001	54.9% (48.4,61.4%); p<0.0001	54.6% (48.1,61.2%); p<0.0001

[a]Primary analysis: TMLE with Adaptive Pre-specification; this analysis is restricted to participants who continued in the extension and had their endpoint ascertained.
[b]TMLE with Super Learner (SL); this is the main sensitivity analysis with additional results in Table S4a.
[c]TMLE with SL and bounding of the estimated propensity scores in [0.2, 1].
[d]TMLE with main terms logistic regressions (GLMs).

[e]TMLE with SL, but only adjusting for baseline characteristics.
[e]TMLE with GLMs, but only adjusting for baseline characteristics.

TABLE S5. CHARACTERISTICS OF STUDY PARTICIPANTS WITH INCIDENT HIV INFECTIONS

	Age in Years	Gender	Study Arm	Country	Date of First Positive HIV Test	Type of HIV Test for First Positive Test	Study Week of First Positive HIV Test	HIV RNA Copies/mL	Reported Use of Oral PrEP, PEP or CAB-LA in 3 months Prior to Seroconversion	Risk Behavior Reported
SP21412030	35	Female	SoC	Uganda	2/1/23	Rapid antibody	4	131,000	None	Partner with unknown HIV status
SP31571035-3	43	Male	SoC	Uganda	6/27/23	Rapid antibody	24	436,000	None	Multiple partners of unknown HIV status
SP31571034-1	42	Male	SoC	Uganda	6/29/23	Rapid antibody	24	Not detected	None	Multiple partners of unknown HIV status
SP35571045-1	33	Female	SoC	Kenya	7/6/23	HIV RNA[1]	24	113,000	None	Inheritor of unknown HIV status[3]
SP25012190	30	Female	SoC	Kenya	8/17/23	Rapid antibody	24	232,000	Inconsistent oral PrEP	Partner with known HIV
SP11007002[2]	23	Female	SoC	Uganda	11/5/23	Rapid antibody	38	1,110,000	None	Multiple partners of unknown HIV status
SP11004204	35	Female	SoC	Uganda	11/17/23	HIV RNA[1]	48	287	None	Husband with unknown HIV status

[1]Subsequent confirmation of HIV diagnosis by HIV antibody tests [2] 7-month-old infant of participant found to have HIV infection by HIV PCR testing on 11/6/23 [3]Participant had sexual relationship by “inheritor” according to a Luo “widow cleansing” tradition after death of a husband (Perry, JIAS, 2014).

TABLE S6. SENSITIVITY ANALYSES FOR BIOMEDICAL HIV PREVENTION COVERAGE DURING PERIODS OF SELF-REPORTED HIV RISK

The following sensitivity analyses were designed to assess the robustness of the secondary endpoint findings when accounting for overall and by-arm differences between participants who did vs. did not enroll in the extension and participants who did vs. did not have their endpoint ascertained in the extension. See the Statistical Analysis Plan for details.

TABLE S6A. RESULTS FROM THE SECONDARY ENDPOINT ANALYSIS AND THE MAIN SENSITIVITY ANALYSIS FOR BIOMEDICAL HIV PREVENTION COVERAGE DURING PERIODS OF SELF-REPORTED HIV RISK, INCLUDING ARM-SPECIFIC ESTIMATES AND EFFECT ESTIMATES.

	Secondary Endpoint Analysis			Main Sensitivity Analysis		
	**Intervention (95%CI)**	**SoC (95%CI)**	**Difference (95%CI); p-value**	**Intervention (95%CI)**	**SoC (95%CI)**	**Difference (95%CI); p-value**
**Overall**	76.5% (71.1,81.8%)	16.2% (12.7,19.7%)	60.2% (53.8,66.6%); p<0.0001	77.8% (73.3,82.4%)	13.6% (10.3,17.0%)	64.2% (59.0,69.4%); p<0.0001
**Women**	75.9% (70.6,81.2%)	18.7% (14.7,22.8%)	57.2% (50.5,63.9%); p<0.0001	76.5% (71.6,81.3%)	15.9% (12.2,19.6%)	60.5% (54.8,66.2%); p<0.0001
**Men**	78.0% (69.2,86.8%)	9.6% (4.7,14.6%)	68.3% (58.2,78.5%); p<0.0001	75.8% (70.1,81.5%)	7.3% (3.5,11.0%)	68.6% (62.0,75.2%); p<0.0001
**15-24 years**	78.1% (71.4,84.9%)	14.8% (9.4,20.3%)	63.3% (54.6,72.1%); p<0.0001	78.8% (73.0,84.7%)	10.1% (5.5,14.7%)	68.7% (61.7,75.7%); p<0.0001
**25+ years**	75.9% (69.6,82.3%)	16.8% (12.5,21.1%)	59.1% (51.5,66.8%); p<0.0001	73.9% (68.6,79.1%)	15.3% (11.0,19.6%)	58.5% (52.0,65.0%); p<0.0001

TABLE S4B. ADDITIONAL SENSITIVITY ANALYSES FOR BIOMEDICAL HIV PREVENTION COVERAGE DURING PERIODS OF SELF-REPORTED HIV RISK, SHOWN AS POINT ESTIMATE OF THE EFFECT WITH 95% CONFIDENCE INTERVALS AND RESULTING P-VALUE.

	**Secondary Endpoint Analysis[a]**	**TMLE with SL[b]**	**TMLE with SL & Bounding[c]**	**TMLE with GLMs[d]**	**TMLE with SL; baseline[e]**	**TMLE with GLMs; baseline[f]**
**Overall**	60.2% (53.8,66.6%); p<0.0001	64.2% (59.0,69.4%); p<0.0001	58.9% (51.8,66.0%); p<0.0001	58.9% (51.8,66.0%); p<0.0001	59.6% (53.6,65.6%); p<0.0001	59.6% (53.6,65.6%); p<0.0001
**Women**	57.2% (50.5,63.9%); p<0.0001	60.5% (54.8,66.2%); p<0.0001	55.8% (47.8,63.9%); p<0.0001	55.8% (47.8,63.9%); p<0.0001	56.3% (50.0,62.6%); p<0.0001	56.3% (50.0,62.6%); p<0.0001
**Men**	68.3% (58.2,78.5%); p<0.0001	68.6% (62.0,75.2%); p<0.0001	67.6% (57.4,77.8%); p<0.0001	67.6% (57.4,77.8%); p<0.0001	68.0% (58.2,77.8%); p<0.0001	68.0% (58.2,77.8%); p<0.0001
**15-24 years**	63.3% (54.6,72.1%); p<0.0001	68.7% (61.7,75.7%); p<0.0001	60.6% (50.2,71.0%); p<0.0001	61.2% (50.2,72.2%); p<0.0001	61.6% (53.5,69.6%); p<0.0001	61.6% (53.5,69.6%); p<0.0001
**25+ years**	59.1% (51.5,66.8%); p<0.0001	58.5% (52.0,65.0%); p<0.0001	53.5% (44.7,62.3%); p<0.0001	53.5% (44.7,62.3%); p<0.0001	58.4% (51.1,65.7%); p<0.0001	58.4% (51.1,65.7%); p<0.0001

[a]Secondary endpoint analysis: TMLE with Adaptive Pre-specification; this analysis is restricted to participants who continued in the extension and had their endpoint ascertained

[b]TMLE with Super Learner (SL); this is the main sensitivity analysis with additional results in Table S6a.

[c]TMLE with SL and bounding of the estimated propensity scores in [0.2, 1].
[d]TMLE with main terms logistic regressions (GLMs).
[e]TMLE with SL, but only adjusting for baseline characteristics
[e]TMLE with GLMs, but only adjusting for baseline characteristics

FIGURE S1. STUDY DESIGN

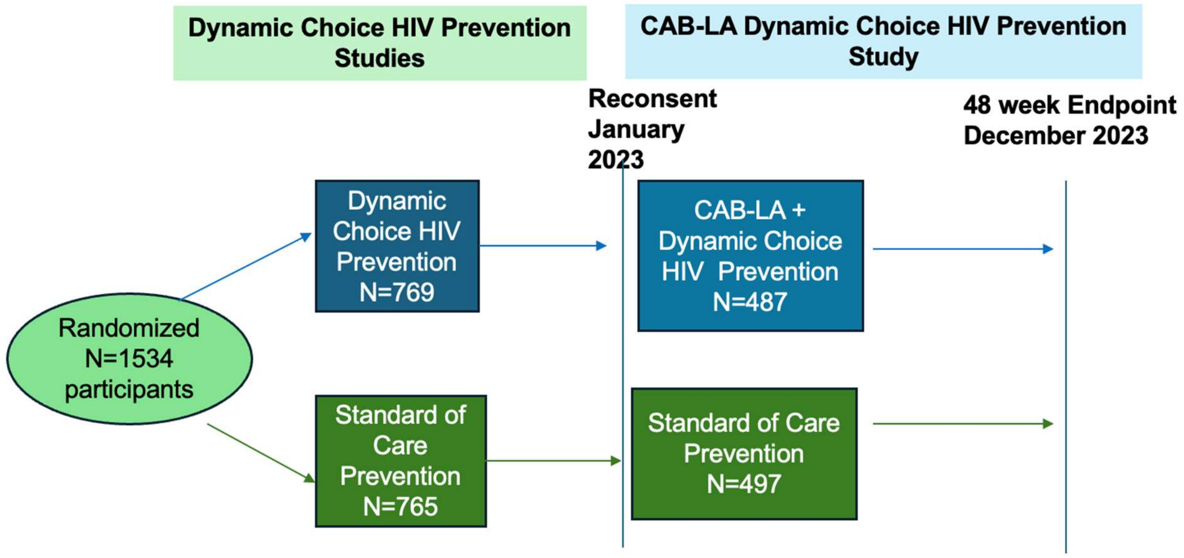

FIGURE S2. EFFECT OF DYNAMIC CHOICE HIV PREVENTION VERSUS STANDARD OF CARE ON HIV BIOMEDICAL PREVENTION COVERAGE DURING PERIODS OF SELF-REPORTED HIV RISK*

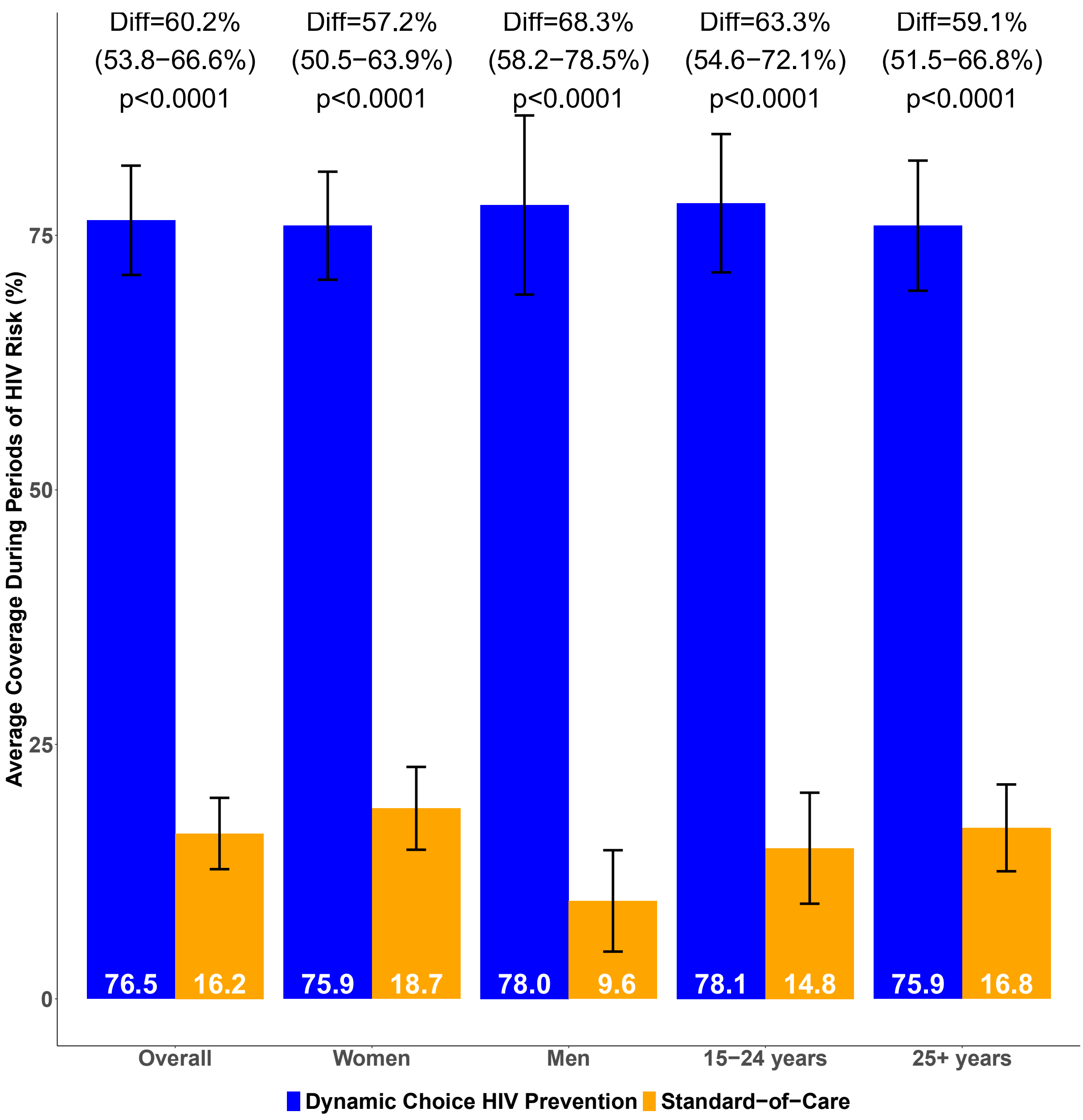

*Proportion of follow-up time that a participant reported being at risk of HIV acquisition during which they were covered by a biomedical prevention option, in each trial arm and their difference. Diff=absolute difference in mean biomedical prevention coverage during periods at risk between arms. 95% confidence intervals are shown in parentheses. p-values use Bonferroni adjustment for multiple testing.
